# Enriching Spiritual Care in Medical Residents Through Cultural Humility and Courage

**DOI:** 10.15766/mep_2374-8265.11423

**Published:** 2024-07-26

**Authors:** Benjamin Drumright, Yasmin Sacro, Abraham Nussbaum

**Affiliations:** 1 Third-Year Resident, Department of Internal Medicine, University of Colorado School of Medicine; 2 Program Director, Primary Care Residency Track, University of Colorado at Denver Health; Associate Professor of Medicine, University of Colorado School of Medicine; 3 Chief Education Officer, Office of Education, Denver Health; Professor of Psychiatry, University of Colorado School of Medicine

**Keywords:** Courage, Cultural Humility, Meaning, Spiritual Care, Case-Based Learning, Communication Skills, Cultural Competence, Internal Medicine, Spirituality, Diversity, Equity, Inclusion

## Abstract

**Introduction:**

While many patients desire spiritual care, it is infrequently provided by physicians. When a model of cultural humility and courage is employed, resident physicians can be introduced to the spiritual care of patients.

**Methods:**

We developed this 90-minute, onetime session to speak directly to resident physicians about the relationships between medicine and spirituality and the nature of spiritual care. In the session, we facilitated residents in reflecting on their current posture toward spiritual care while addressing its evidence, obstacles, and timing. We also discussed the need for cultural humility and courage as we followed spiritual care to its root: guiding a person in finding meaning in their current circumstances.

**Results:**

We presented this interactive session to 35 internal medicine residents from all four training years. All residents responded to an embedded pre- and postsurvey question modeled after four attitudes towards spiritual care: rejecting, guarded, pragmatic, and embracing. Out of 22 residents who did not report embracing spiritual care in the presession survey, 10 (45%) reported a more positive attitude toward spiritual care on their postcourse surveys. Twenty-seven residents in attendance (77%) also provided feedback about presentation quality, with a mean rating of 4.7 out of 5 indicating overall satisfaction.

**Discussion:**

A single well-received session on spiritual care for medical residents models the integration of relevant spiritual care curricula into residency training. The resulting module can be modified for physicians of any specialty or seniority and complemented by other skill-based spiritual care curricula.

## Educational Objectives

By the end of this activity, learners will be able to:

1. Define spirituality and the spiritual care of patients.
2. Evaluate evidence in support of spiritual care by physicians.
3. Name common obstacles to spiritual care provision by physicians.
4. Identify moments when spiritual care by physicians is most appropriate.
5. Discuss cultural humility and courage as part of spiritual care.
6. Reflect on their current posture toward spiritual care using the four attitudes framework.

## Introduction

While many patients desire spiritual care, providing such care can be intimidating for physicians. Medicine struggles to guide physicians, especially trainees, in understanding the spiritual life of patients and the provision of spiritual care in part due to a collective ambivalence. On one hand, medical governing bodies require its provision for patients as an aspect of cultural competency. On the other hand, spirituality can be viewed as an obstacle to both the empiricism and the efficiency of modern health care. This ambivalence often leads physicians to make spiritual care a low-priority task, and most physicians simply do not actively engage in spiritual care.^1^ While most medical schools incorporate spiritual care into their curricula, it is less frequently experienced in clinical training.^2^ The momentum is further lost in the crucible of residency, where training in spiritual care is uncommon.

We developed this 90-minute, onetime session to speak directly to resident physicians about the relationship between medicine and spirituality and the nature of spiritual care. We utilized an interactive lecture, intentional self-reflection, and frequent group discussions. We chose this approach to allow adult learners to engage deeply with the content while being able to manage their own self-disclosure.

There are currently no publications regarding spiritual care in *MedEdPORTAL* directed toward a resident physician audience. Our session supplements introductory materials for medical students already available in *MedEdPORTAL* that emphasize gathering a spiritual history, though none are prerequisites for our training.^3–5^ This session also complements a case-based module from the Tanenbaum Center for Interreligious Understanding that addresses some of medicine's most contentious topics related to patient spirituality.^6^ Our session focuses more on personal reflection while addressing the evidence for, obstacles to, and timing and definition of spiritual care.

To welcome learners of all spiritual backgrounds, we name the root of spiritual care as guiding a person in finding meaning in their current circumstances. We then empower residents by demonstrating that their training and life experiences have equipped them to be a worthy guide for patients. Although this approach simplifies spiritual care, we believe it models cultural humility and requires courage. Our intent is to inspire interested residents to the imperfect pursuit of these key virtues if motivated to engage with patients in spiritual care.

## Methods

We developed a 90-minute session with a presentation entitled Spiritual Care 201: Cultural Humility & Courage in Spiritual Care. The learning objectives provided resident physicians with sufficient means to reflect on the relationship of spirituality to their physician identity and the skills to participate in spiritual care.

A physician facilitated the entire 90-minute session, with the majority of time spent on didactic overviews of key concepts. We dedicated approximately 30 minutes of the session to short, guided reflections. We included three simple cases to bring practicality and elicit preliminary thoughts from the audience while transitioning to new concepts. We delivered the session during ambulatory didactic time to an audience of about nine residents on average.

Not all residents entered the session with equivalent prior knowledge of spiritual care. Some had not participated in any curricula related to spirituality. Spiritual care experience in medical school would have provided helpful context for this session but was not a prerequisite. The session briefly mentioned common introductory topics such as spiritual screening questions and the chaplaincy, though a detailed discussion was outside the session's scope.

Audiovisual requirements included a computer with projector and PowerPoint set up for the included presentation (Appendix A). We used an internet connection for exploring the Pew Research Center website.^7^ We developed a facilitator guide (Appendix B) with in-depth exposition of each presentation slide. Befitting our target audience of residents, we did not assign any prework.

At the beginning of the session, we distributed a half-sheet of reflection questions for residents to follow along and take notes on, if desired (Appendix C). We began and ended the session with a multiple-choice survey question: “What attitude fits your current posture toward spiritual care?” Possible responses were adapted from the four attitudes toward spiritual care identified by Appleby, Wilson, and Swinton, including rejecting, guarded, pragmatic, and embracing.^8^ The first intent of this question was to evaluate the session's influence on resident physician attitudes. Its second intent was to facilitate honest self-reflection in residents about biases for or against spiritual care prior to receiving any information. We embedded an online-generated QR code in the slides for data collection but have removed it for publication here. After the session, we emailed residents an institutionally standardized postcourse survey (Appendix D) for ambulatory didactics regarding the quality of the presentation.

## Results

Over the course of 6 months and four sessions, we had 35 internal medicine residents from all four training years participate in the educational session. All residents responded to the embedded pre- and postsurveys about their attitude toward spiritual care. Twenty-seven residents filled out the emailed postcourse survey with more general questions about presentation quality.

Presurvey data about current attitudes toward spiritual care revealed that one resident (3%) described themselves as rejecting, four (11%) as guarded, 17 (49%) as pragmatic, and 13 (37%) as embracing (Figure). Postsurvey data about current attitudes toward spiritual care revealed that no residents (0%) described themselves as rejecting, two (6%) as guarded, 10 (29%) as pragmatic, and 23 (66%) as embracing (Figure). Out of 22 residents who did not report embracing spiritual care in the presurvey, there were 10 (45%) with a more positive attitude toward spiritual care in their postsurvey.

**Figure.**
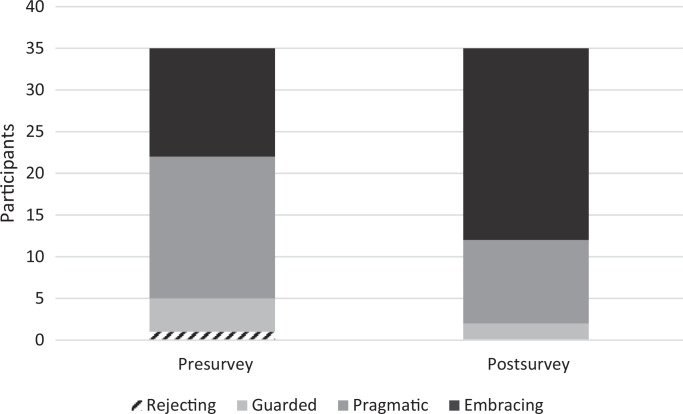
Participants’ attitude toward spiritual care pre- and postsession (*N* = 35).

Twenty-seven residents responded to the emailed postcourse survey about presentation quality, where responses were indicated based on a 5-point Likert scale (1 = *strongly disagree,* 5 = *strongly agree*). Residents rated the session with mean values of 4.7 for being well organized, 4.6 for being relevant, 4.8 for meeting learning objectives, and 4.7 for being satisfying overall (Table).

**Table.**
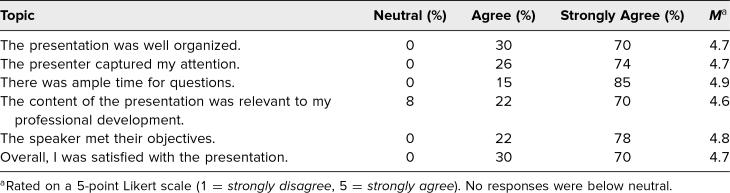
Postcourse Survey Regarding Presentation Quality (*N* = 27)

Some of the more detailed qualitative responses included the following:

• “This was a great session on an important topic that is not discussed frequently.”
• “Great presentation on an important topic. Appreciated the thoughtful and humble approach.”
• “Very thoughtful and thought provoking. Lots of great evidence and quotes. Wondering about more concrete options for engaging with this and tool sets.”

## Discussion

Discussing spiritual care with patients is a neglected and undertaught skill. Training in spiritual care during the uniquely challenging and formational season of residency is critical for physicians to be empowered to offer spiritual care throughout their careers. In this low-resource seminar, we sought to provide resident physicians with the means to reflect on the relationship of spirituality to their physician identity and the skills to participate in spiritual care. The session serves as a model for how relevant spiritual care curricula can be easily integrated into residency. Ideally, it would not be delivered at the beginning of the academic year in order to allow first-year residents to gain life experiences as a doctor on which to reflect. Our exploration of the foundations of spiritual care can also be made relevant to physicians of any specialty or seniority with minimal modification of the included cases. The session can accommodate larger groups, though additional facilitators may be helpful to divide larger group discussions.

We sought to train without endorsing any specific spirituality, to model cultural humility, and to allow all residents to evaluate the advantages and costs of spiritual care for themselves, while hoping that some might be inspired to incorporate spiritual care into their practice. The crux of this session is that the root of spiritual care is guiding a patient in finding meaning in their current circumstances. This is not a novel idea but is an integration of more lengthy definitions in the literature. However, what is unique about this spirituality session is examining spiritual care through the lenses of cultural humility and courage. One of the strengths of these approaches is their simplicity. Physicians with minimal prior exposure to spiritual care can walk away from the session able to apply these profound concepts.

Overall, the session was well received. Responses suggest that residents found the session met its objectives and was well organized, relevant, and satisfying. Importantly, there was no evidence that our approach was disagreeable to any resident, as no portions of any evaluations were below neutral. We also found that many residents left the session with more open attitudes toward spiritual care than they had before the learning event.

The main limitation is in the assessment of measurable outcomes in participant behaviors. The embedded pre- and postsurveys inquired about attitudes towards spiritual care but did not evaluate spiritual care skills or knowledge retention. We relied on a postcourse survey asking participants if the session met its stated objectives rather than asking them to demonstrate their knowledge. These intentional choices reflected our fundamental aim: to inspire resident physicians to pursue cultural humility and develop courageous interest in spiritual care. We structured the session so concepts of humility and courage, rather than knowledge or skill acquisition, would be the climactic experience integrating the learning objectives. To address this aim, we focused our assessment on shifts in overall attitude. We used the accessible framework of four attitudes toward spiritual care identified by Appleby, Wilson, and Swinton^8^ to evaluate changes in inspiration. In advanced versions of the course designed to build upon this learning event, posttests for knowledge retention and skill acquisition could be included.

Several other limitations are noteworthy. First, the reproducibility of the session relies on prerequisite knowledge. Although participants need no prior spiritual care training, we assume comprehension of basic clinical ethics, motivational interviewing, and some common terms by a resident physician audience. We have added definitions of each attitude toward spiritual care to the facilitator guide to aid in standardization. Second, our evaluation assesses only reactions to the session. We have not measured level of engagement with the self-reflection questions and are unable to say whether the session leads to long-term changes in approach to care among resident physicians. Lastly, the emphasis on capturing and scrutinizing the essence of spiritual care is in tension with the need for more practical examples. One resident expressed a desire for more concrete options and tools for engaging. A simple solution would be to consider applying the concepts discussed in this session to one or both modules by the Tanenbaum Center for Interreligious Understanding available in *MedEdPORTAL*.^5,6^ Alternatively, a future direction could include a second session of case-based practice exercises to apply concepts in spiritual care scenarios. This would also create the opportunity for assessment of acquisition and application of spiritual care knowledge.

## Appendices


Cultural Humility and Courage in Spiritual Care.pptxFacilitator Guide for Spiritual Care Session.docxSpiritual Care Reflection Questions.docxSpiritual Care Surveys.docx
*All appendices are peer reviewed as integral parts of the Original Publication.*
