# Supplementary material for: Enriching Spiritual Care in Medical Residents Through Cultural Humility and Courage
Source: MedEdPORTAL. 2024 Jul 26;20:11423. doi: 10.15766/mep_2374-8265.11423 (PMC11272909; doi:10.15766/mep_2374-8265.11423)
Supplement: Supplementary file 1 — Cultural Humility and Courage in Spiritual Care.pptxFacilitator Guide for Spiritual Care Session.docxSpiritual Care Reflection Questions.docxSpiritual Care Surveys.docx [file mep_2374-8265.11423-s001.zip › A. Cultural Humility and Courage in Spiritual Care.pptx]

## Slide 1
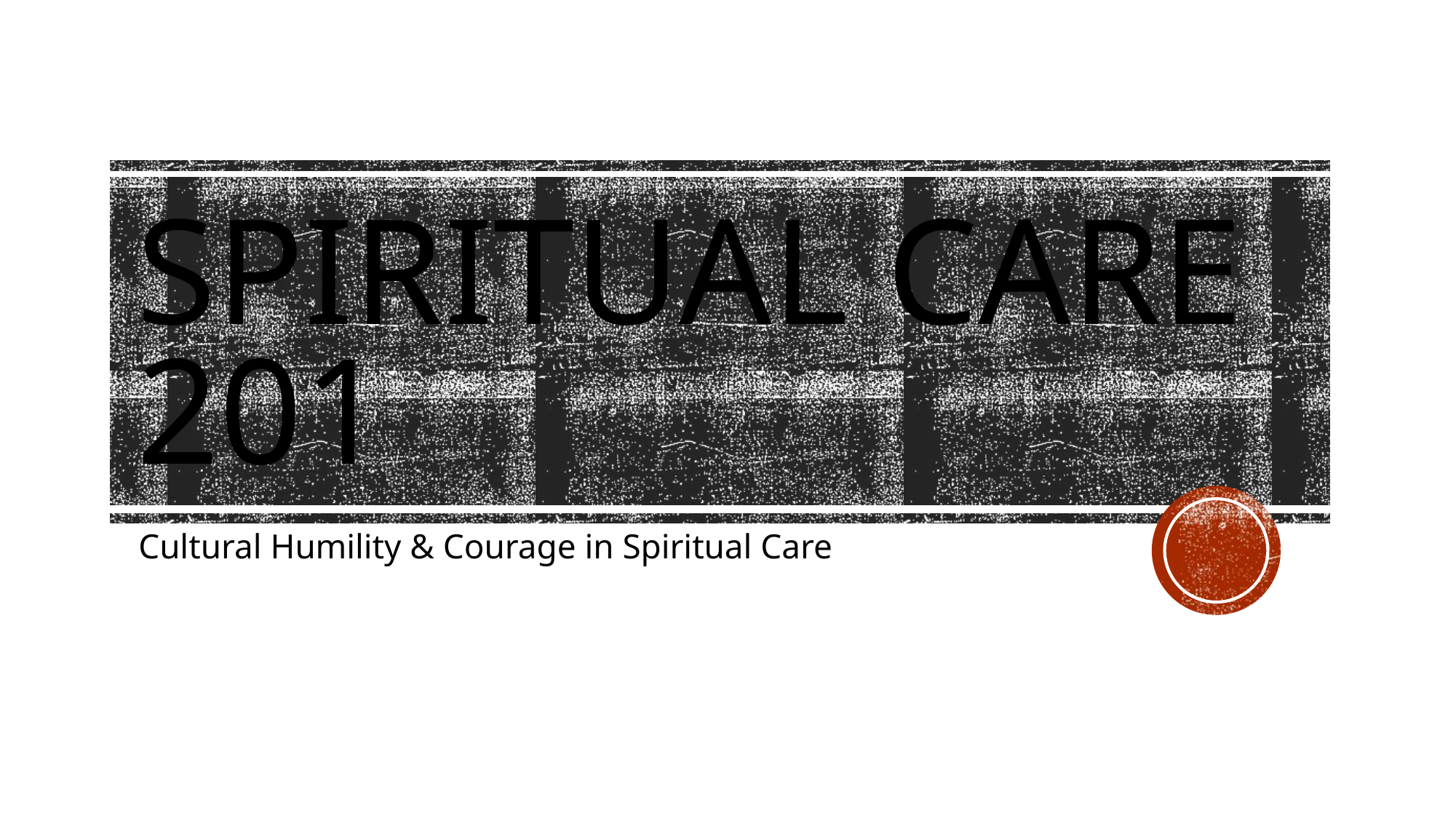

# Spiritual Care 201
Cultural Humility & Courage in Spiritual Care

## Slide 2
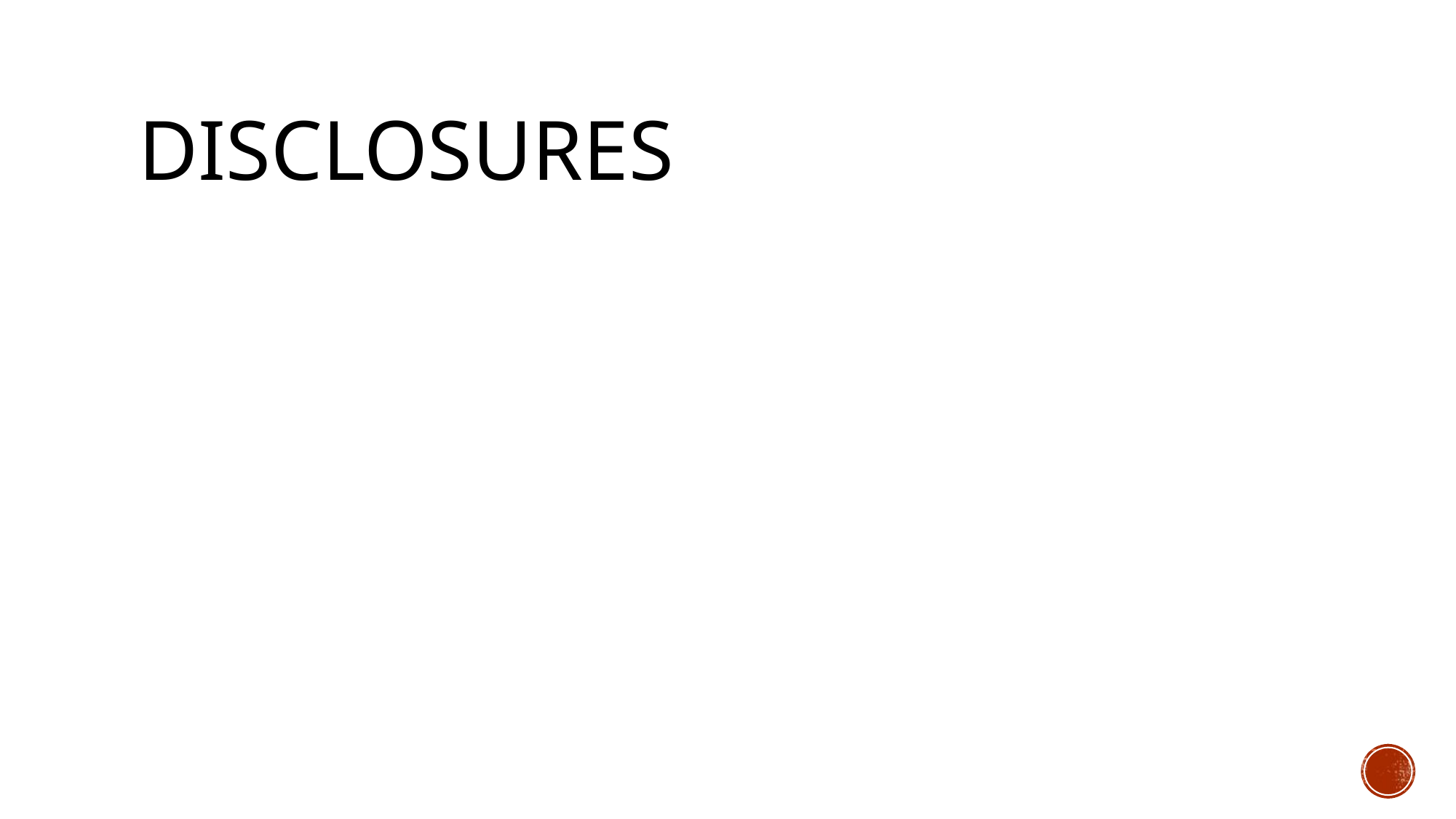

# Disclosures

## Slide 3
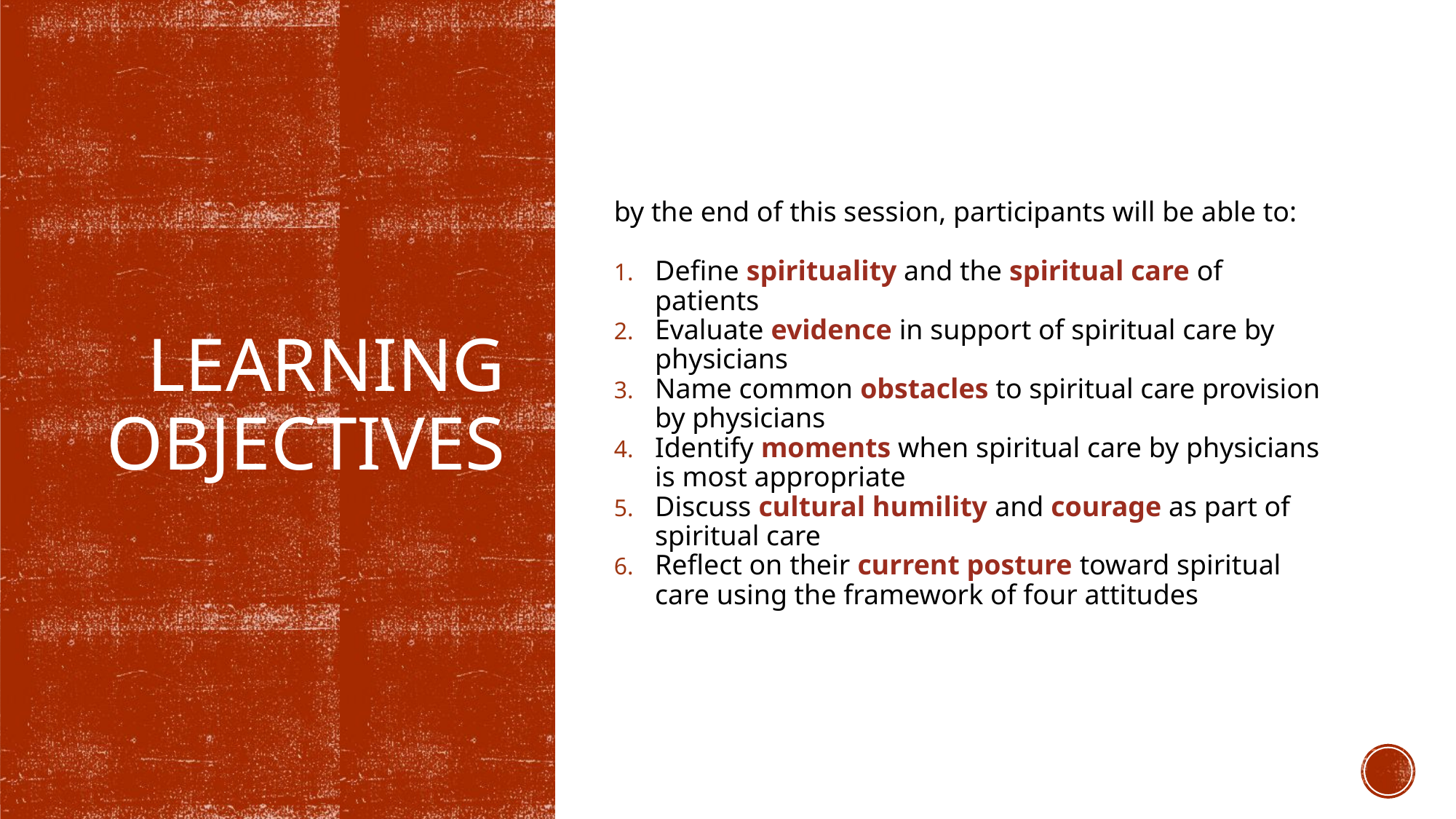

by the end of this session, participants will be able to:
Define spirituality and the spiritual care of patients
Evaluate evidence in support of spiritual care by physicians
Name common obstacles to spiritual care provision by physicians
Identify moments when spiritual care by physicians is most appropriate
Discuss cultural humility and courage as part of spiritual care
Reflect on their current posture toward spiritual care using the framework of four attitudes
# Learning Objectives

## Slide 4
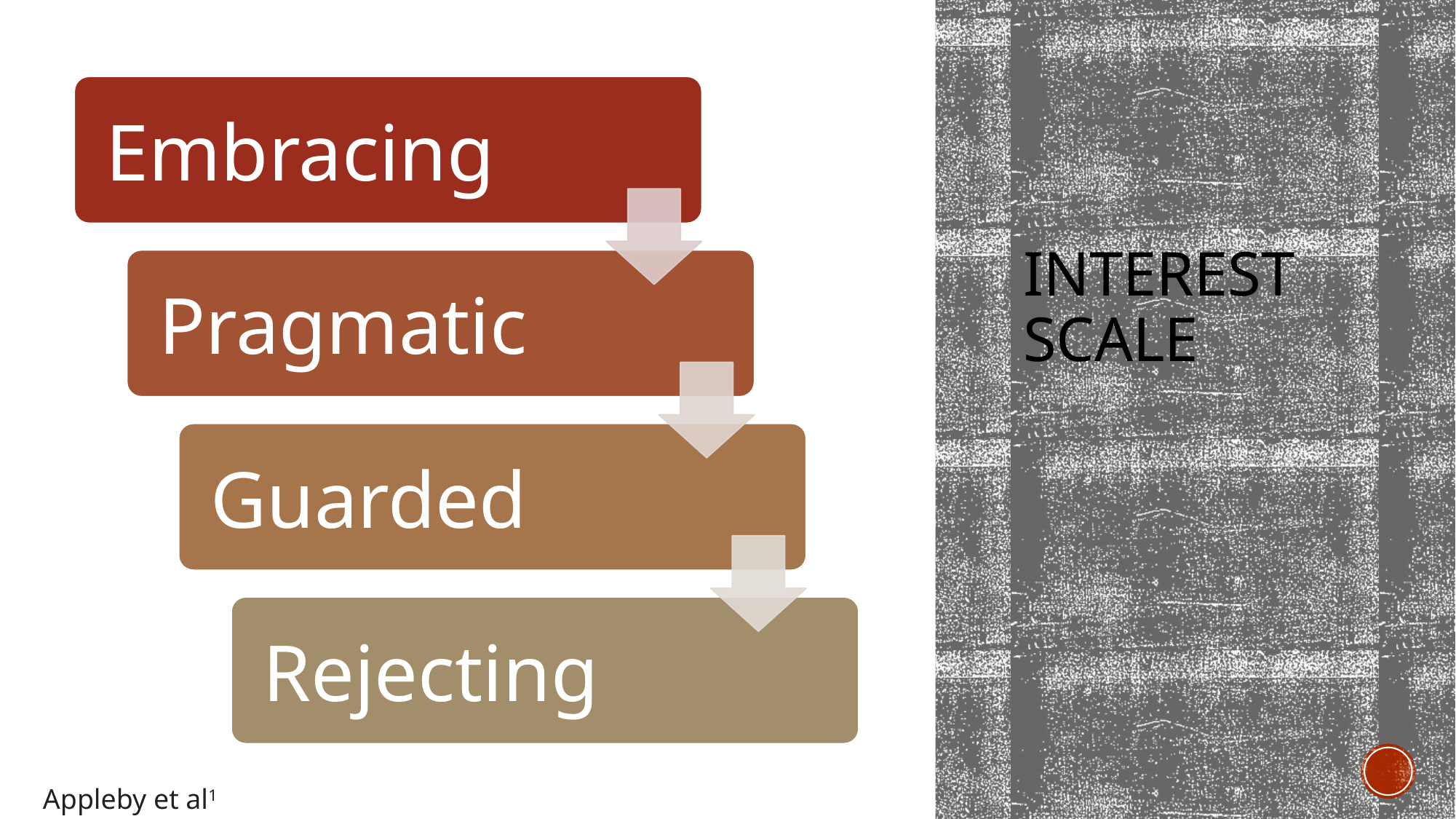

# Interest scale
Appleby et al1

## Slide 5
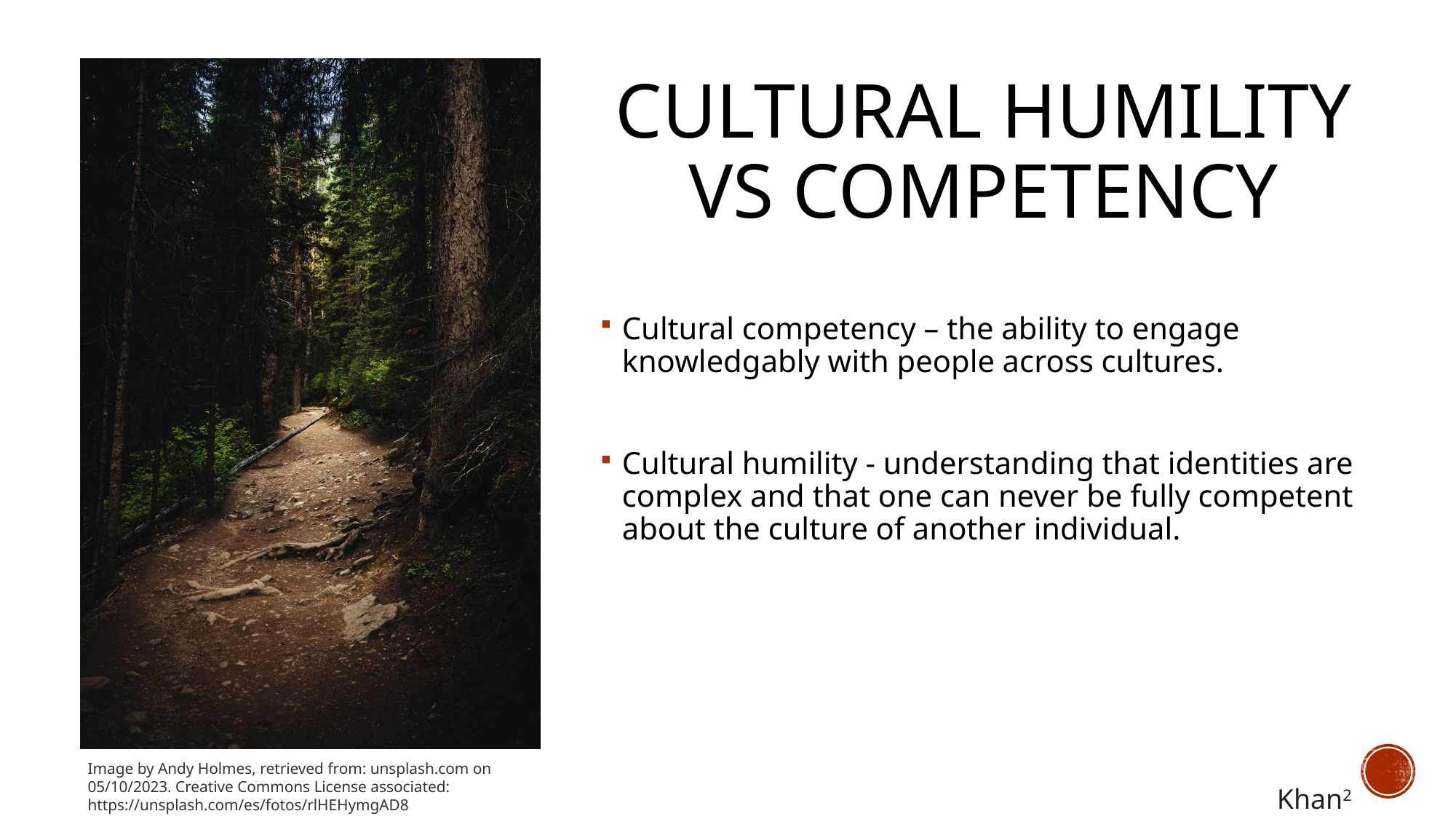

# Cultural Humility vs Competency
Cultural competency – the ability to engage knowledgably with people across cultures.
Cultural humility - understanding that identities are complex and that one can never be fully competent about the culture of another individual.
Image by Andy Holmes, retrieved from: unsplash.com on 05/10/2023. Creative Commons License associated: https://unsplash.com/es/fotos/rlHEHymgAD8
Khan2

## Slide 6
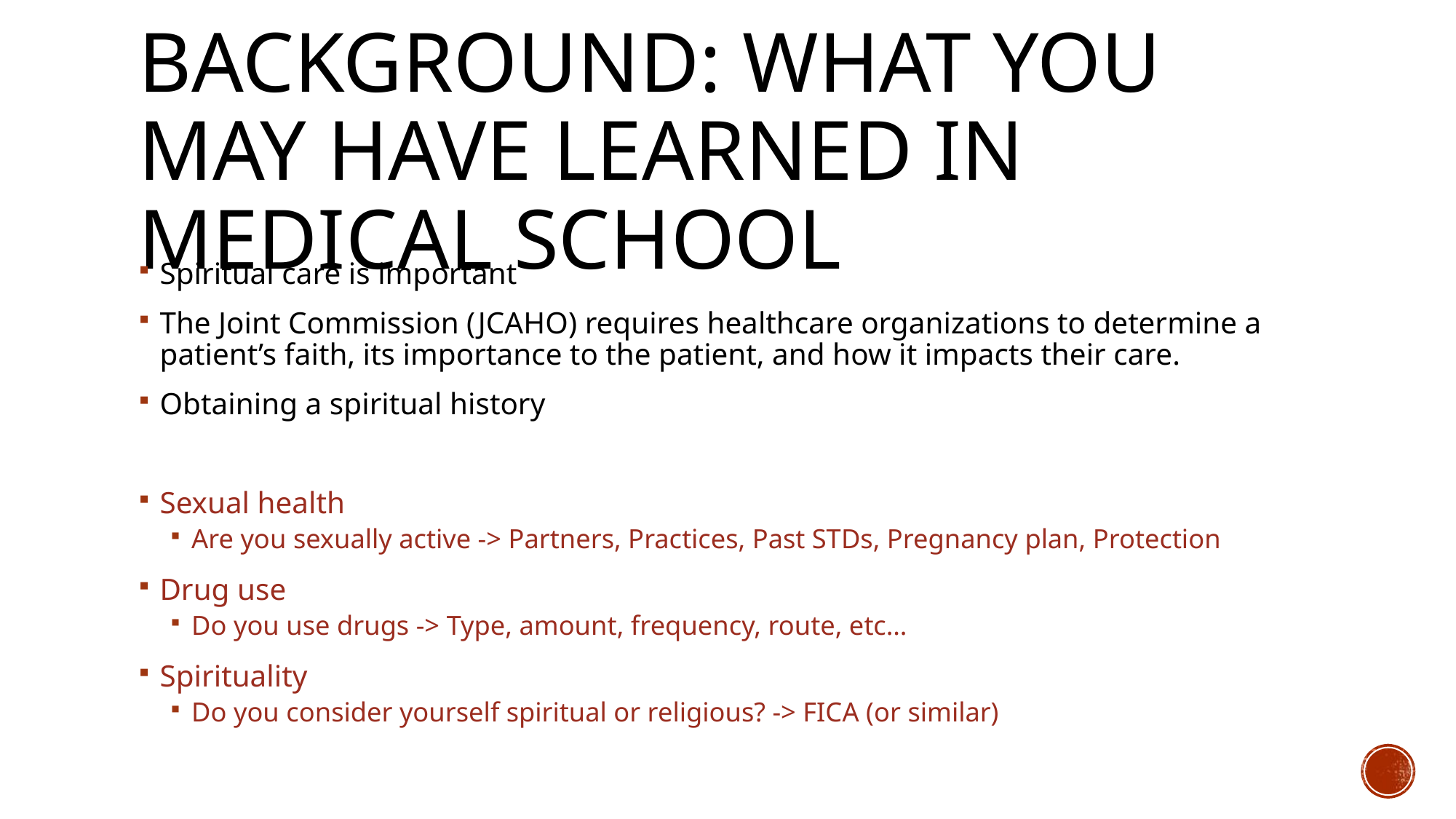

# Background: What you may have learned in medical school
Spiritual care is important
The Joint Commission (JCAHO) requires healthcare organizations to determine a patient’s faith, its importance to the patient, and how it impacts their care.
Obtaining a spiritual history
Sexual health
Are you sexually active -> Partners, Practices, Past STDs, Pregnancy plan, Protection
Drug use
Do you use drugs -> Type, amount, frequency, route, etc…
Spirituality
Do you consider yourself spiritual or religious? -> FICA (or similar)

## Slide 7
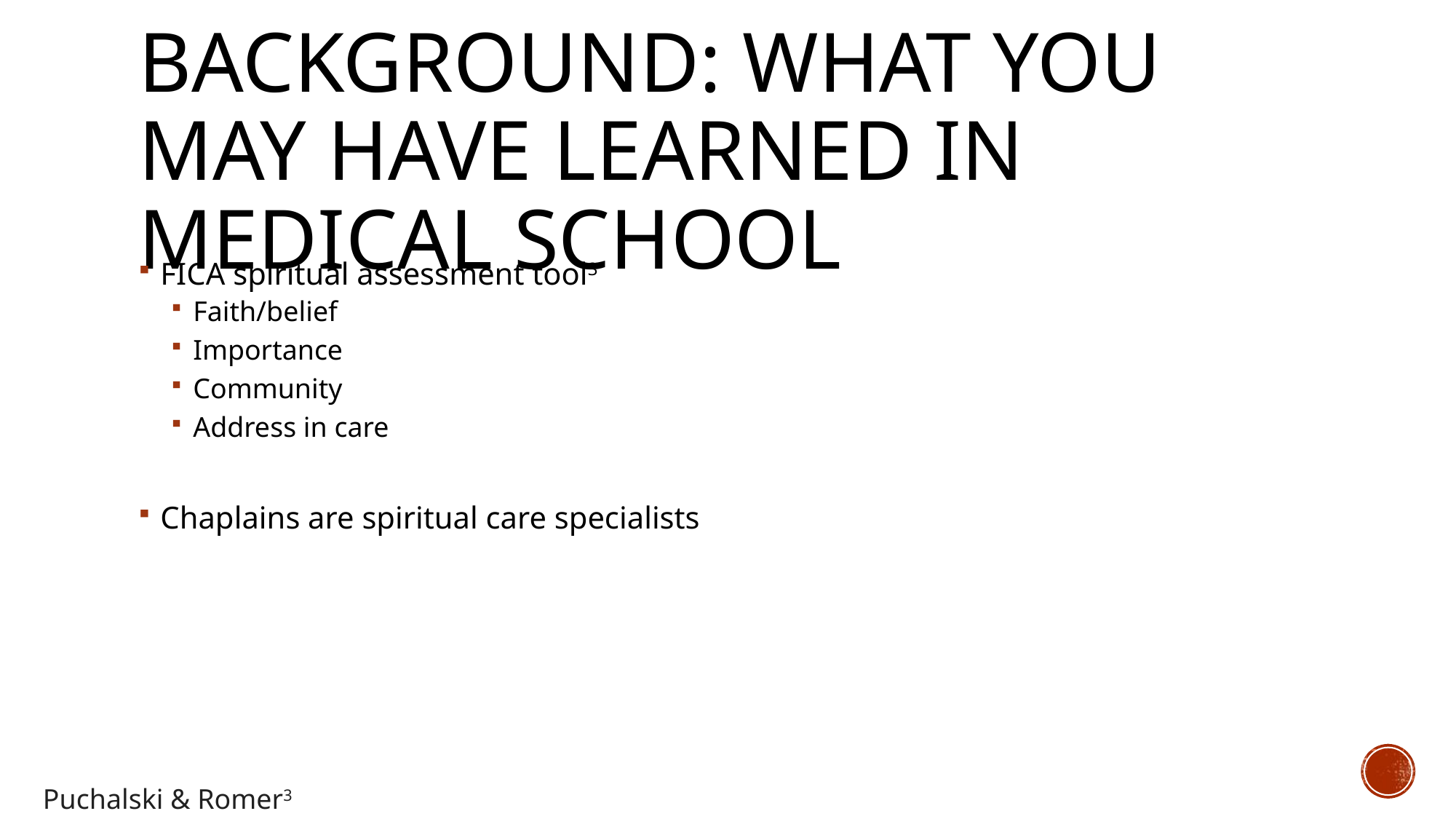

# Background: What you may have learned in medical school
FICA spiritual assessment tool3
Faith/belief
Importance
Community
Address in care
Chaplains are spiritual care specialists
Puchalski & Romer3

## Slide 8
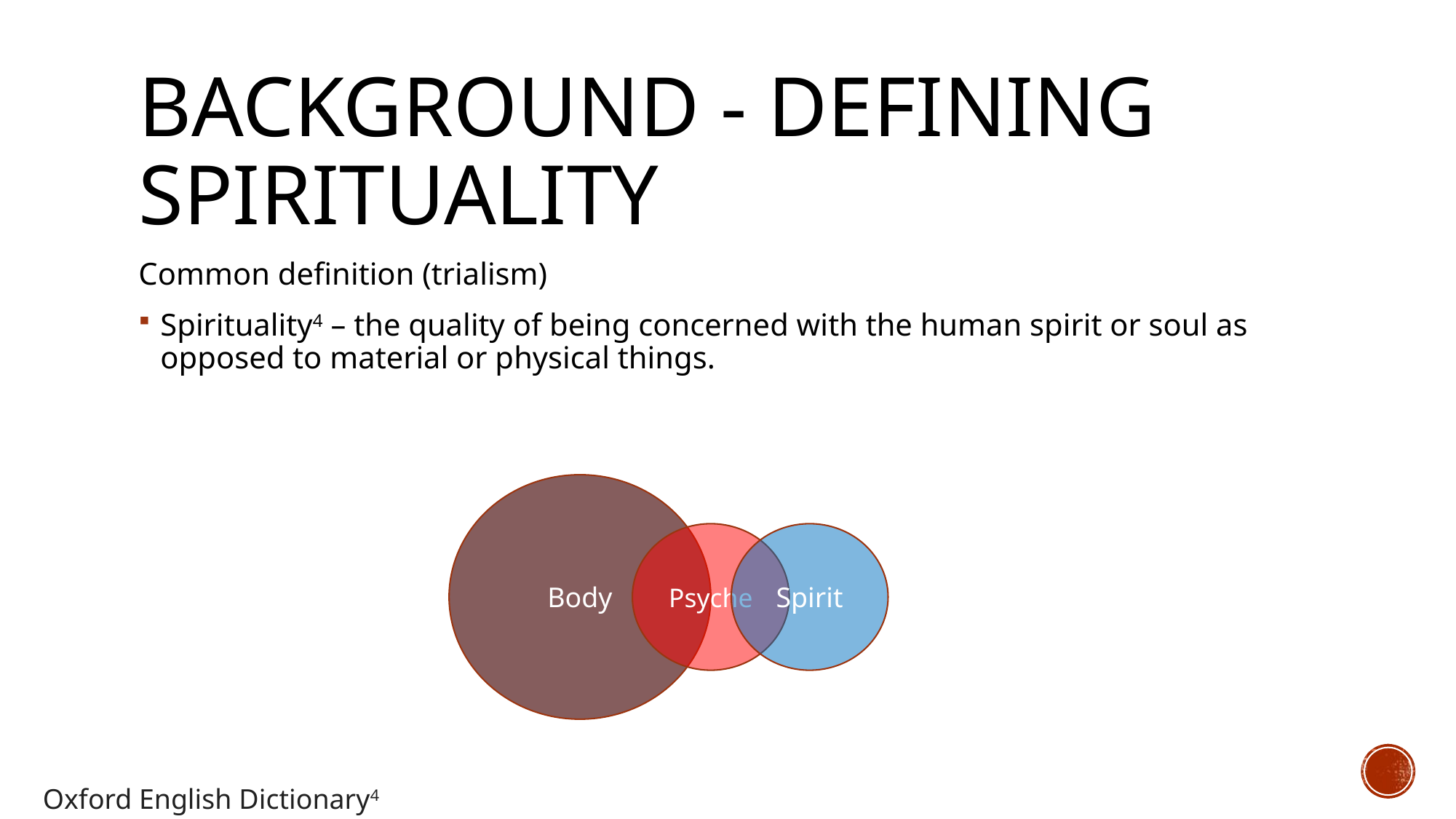

# Background - Defining Spirituality
Common definition (trialism)
Spirituality4 – the quality of being concerned with the human spirit or soul as opposed to material or physical things.
Body
Psyche
Spirit
Oxford English Dictionary4

## Slide 9
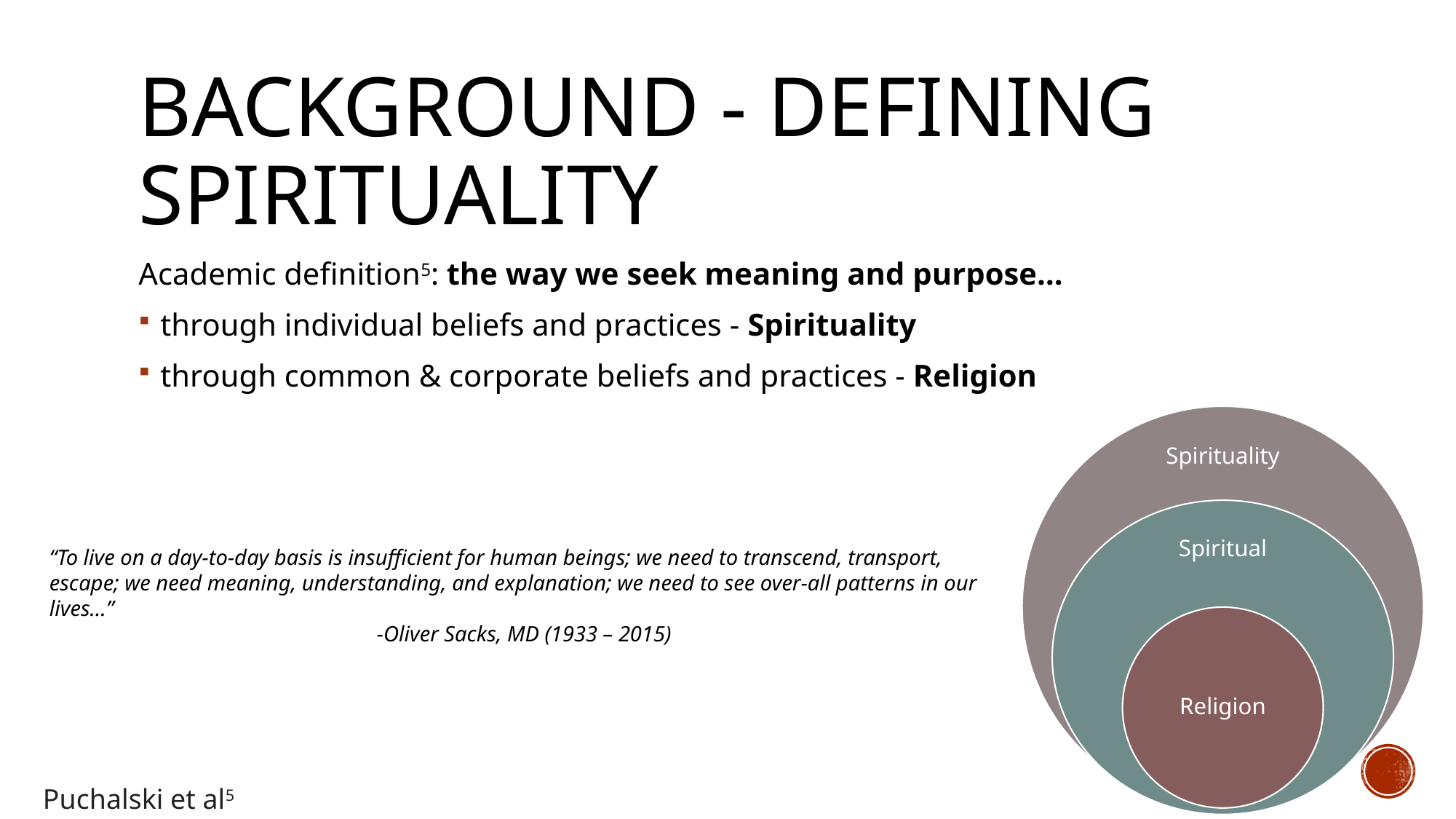

# Background - Defining Spirituality
Academic definition5: the way we seek meaning and purpose…
through individual beliefs and practices - Spirituality
through common & corporate beliefs and practices - Religion
“To live on a day-to-day basis is insufficient for human beings; we need to transcend, transport, escape; we need meaning, understanding, and explanation; we need to see over-all patterns in our lives…”
			-Oliver Sacks, MD (1933 – 2015)
Puchalski et al5

## Slide 10
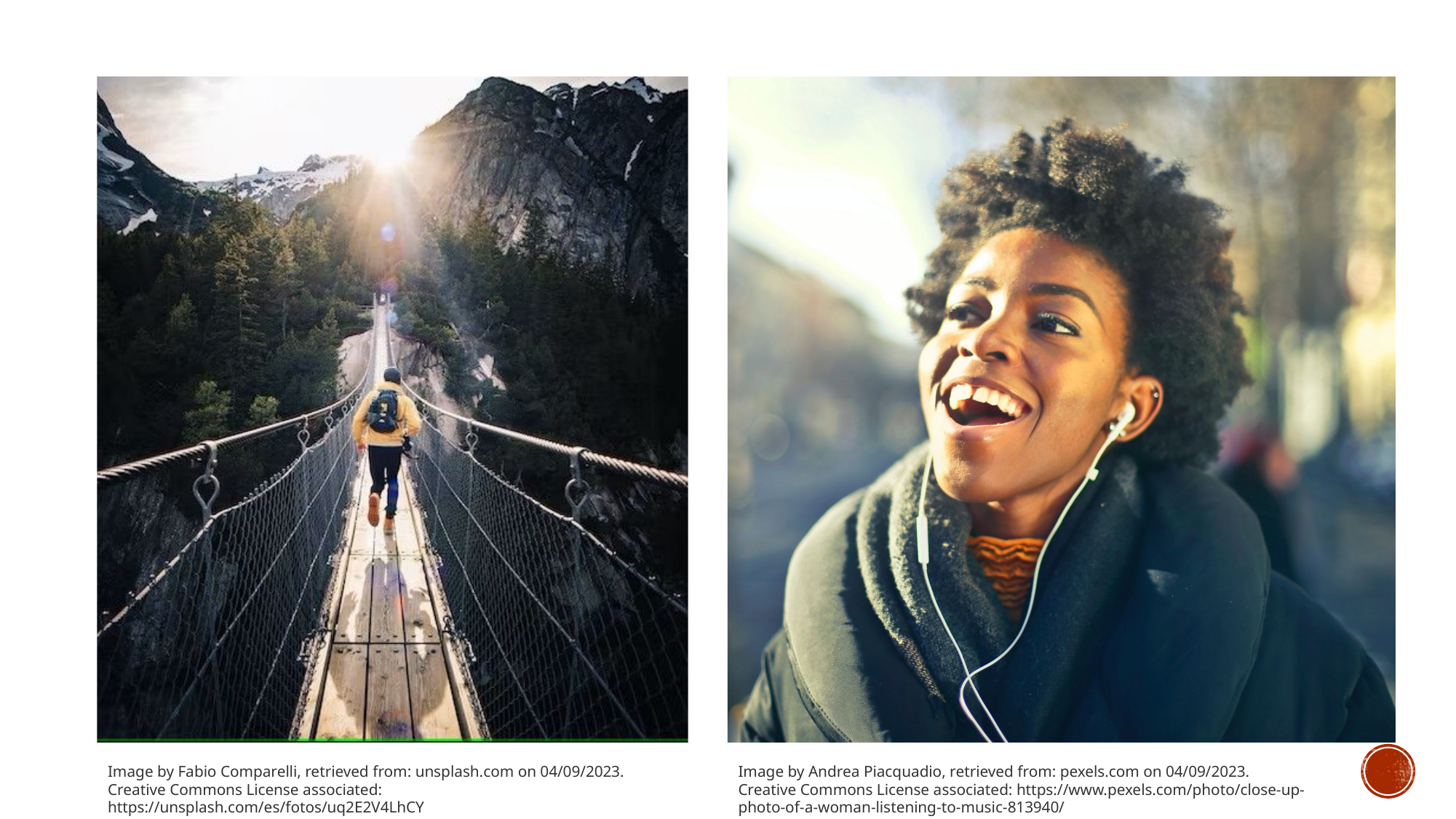

Image by Fabio Comparelli, retrieved from: unsplash.com on 04/09/2023. Creative Commons License associated: https://unsplash.com/es/fotos/uq2E2V4LhCY
Image by Andrea Piacquadio, retrieved from: pexels.com on 04/09/2023. Creative Commons License associated: https://www.pexels.com/photo/close-up-photo-of-a-woman-listening-to-music-813940/

## Slide 11
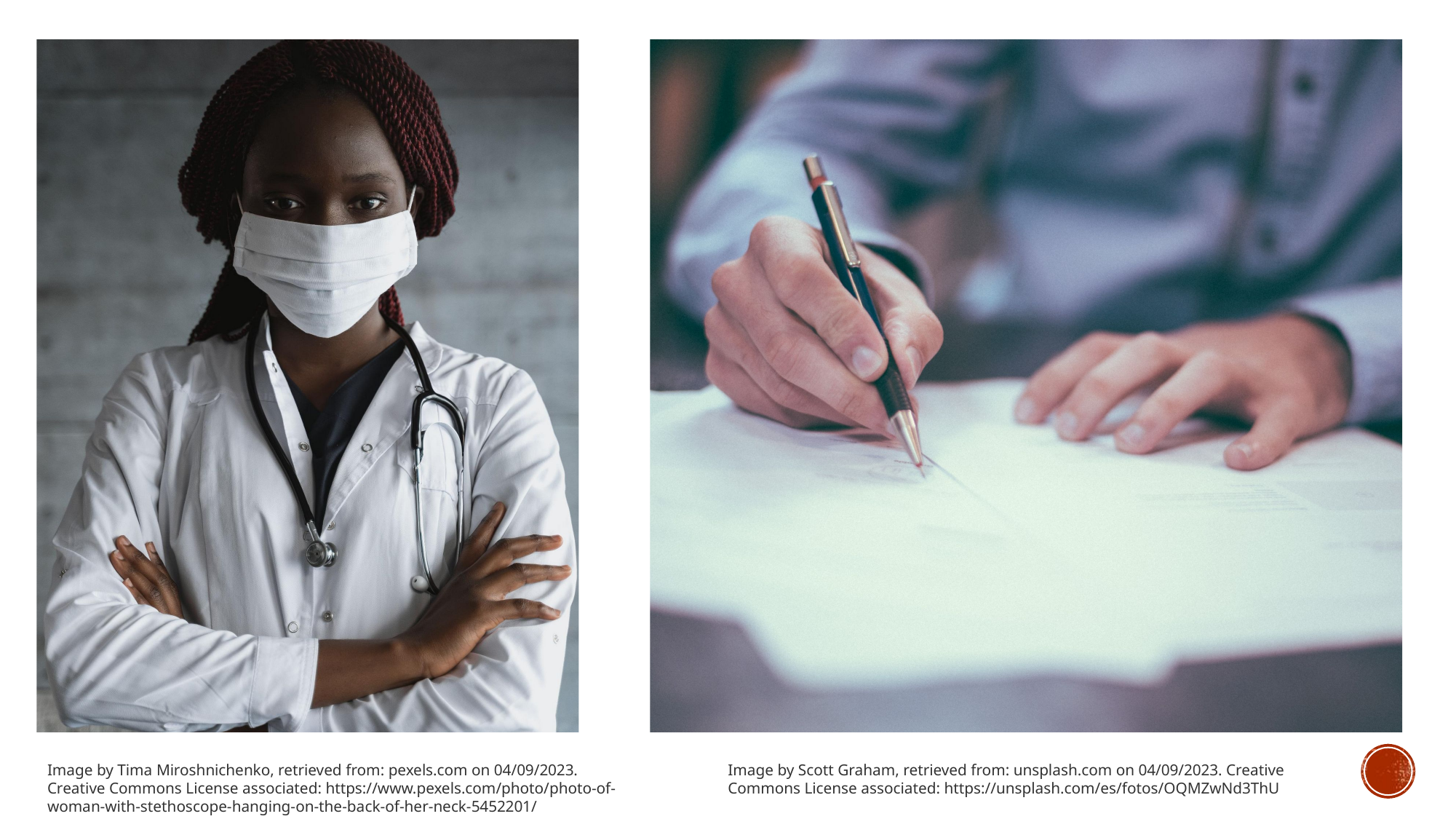

Image by Tima Miroshnichenko, retrieved from: pexels.com on 04/09/2023. Creative Commons License associated: https://www.pexels.com/photo/photo-of-woman-with-stethoscope-hanging-on-the-back-of-her-neck-5452201/
Image by Scott Graham, retrieved from: unsplash.com on 04/09/2023. Creative Commons License associated: https://unsplash.com/es/fotos/OQMZwNd3ThU

## Slide 12
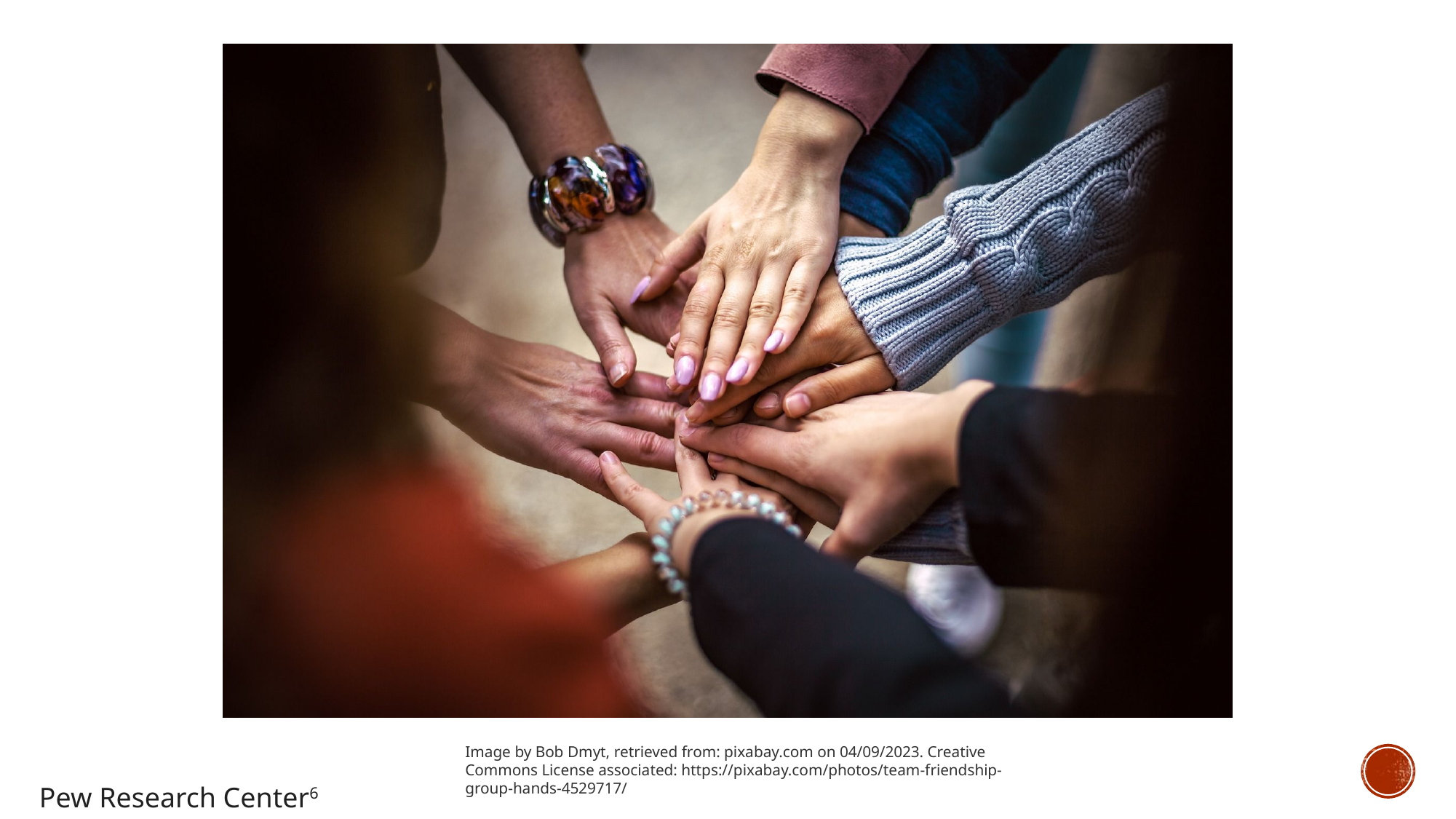

Image by Bob Dmyt, retrieved from: pixabay.com on 04/09/2023. Creative Commons License associated: https://pixabay.com/photos/team-friendship-group-hands-4529717/
Pew Research Center6

## Slide 13
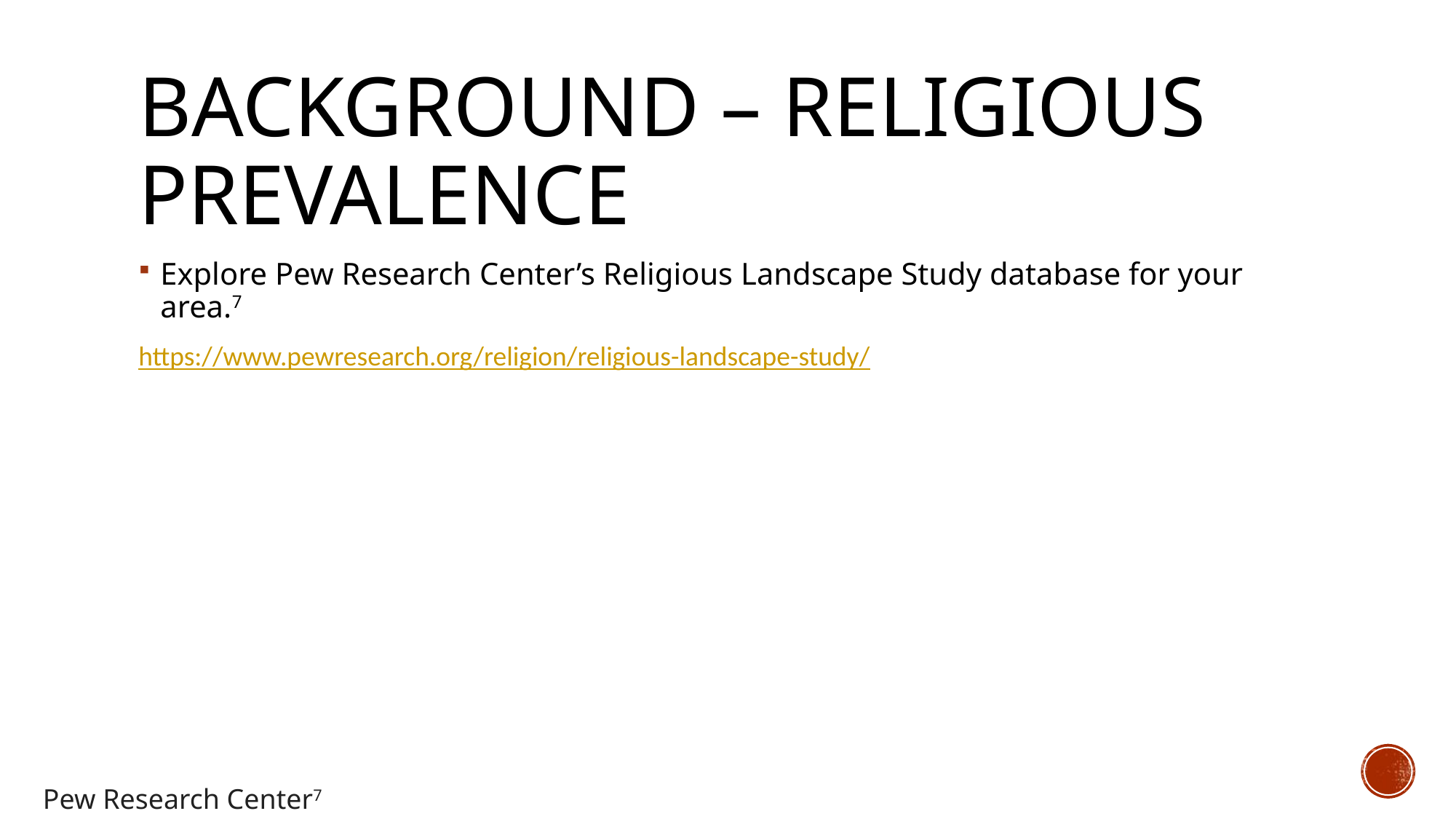

# Background – Religious Prevalence
Explore Pew Research Center’s Religious Landscape Study database for your area.7
https://www.pewresearch.org/religion/religious-landscape-study/
Pew Research Center7

## Slide 14
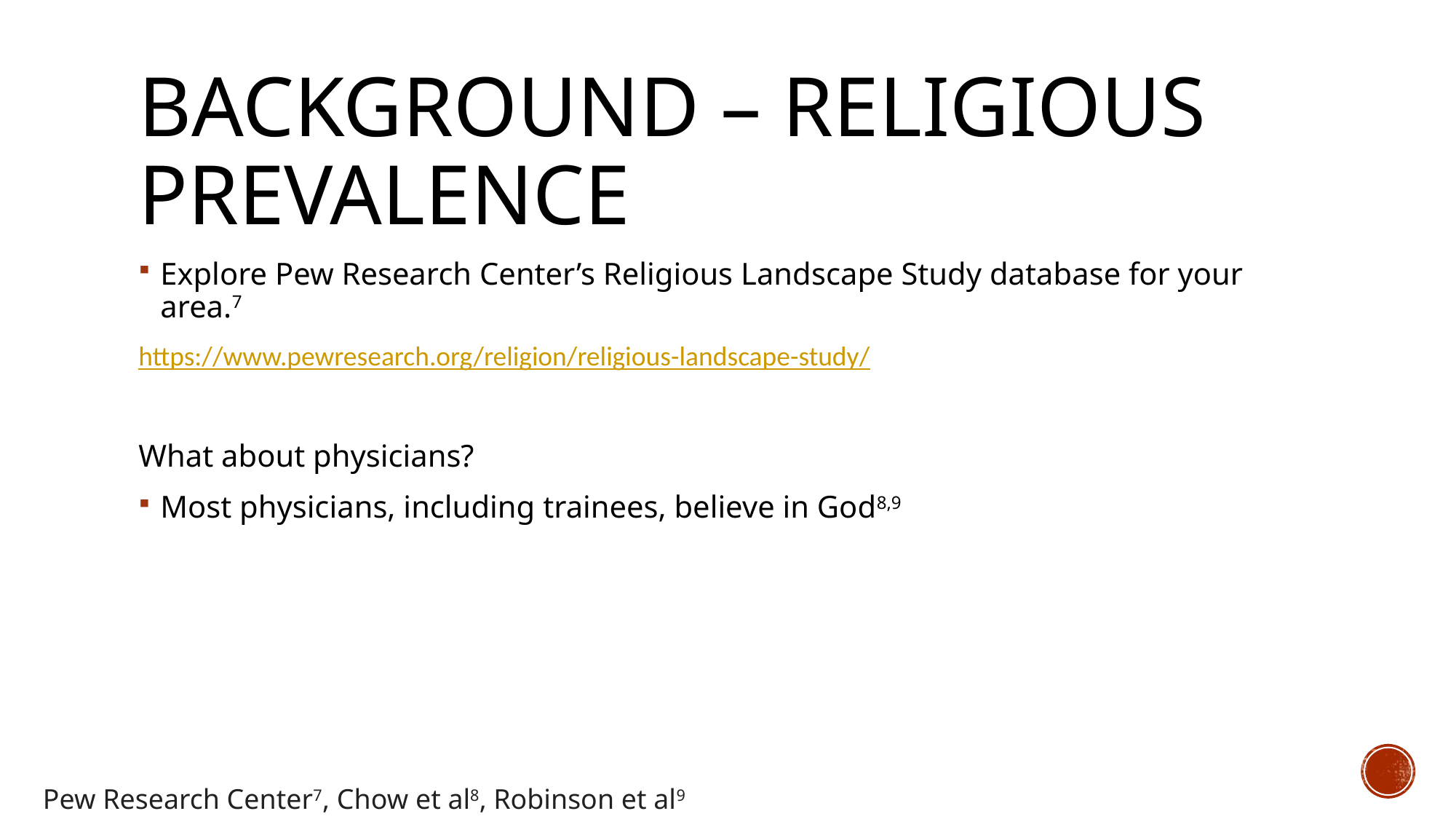

# Background – Religious Prevalence
Explore Pew Research Center’s Religious Landscape Study database for your area.7
https://www.pewresearch.org/religion/religious-landscape-study/
What about physicians?
Most physicians, including trainees, believe in God8,9
Pew Research Center7, Chow et al8, Robinson et al9

## Slide 15
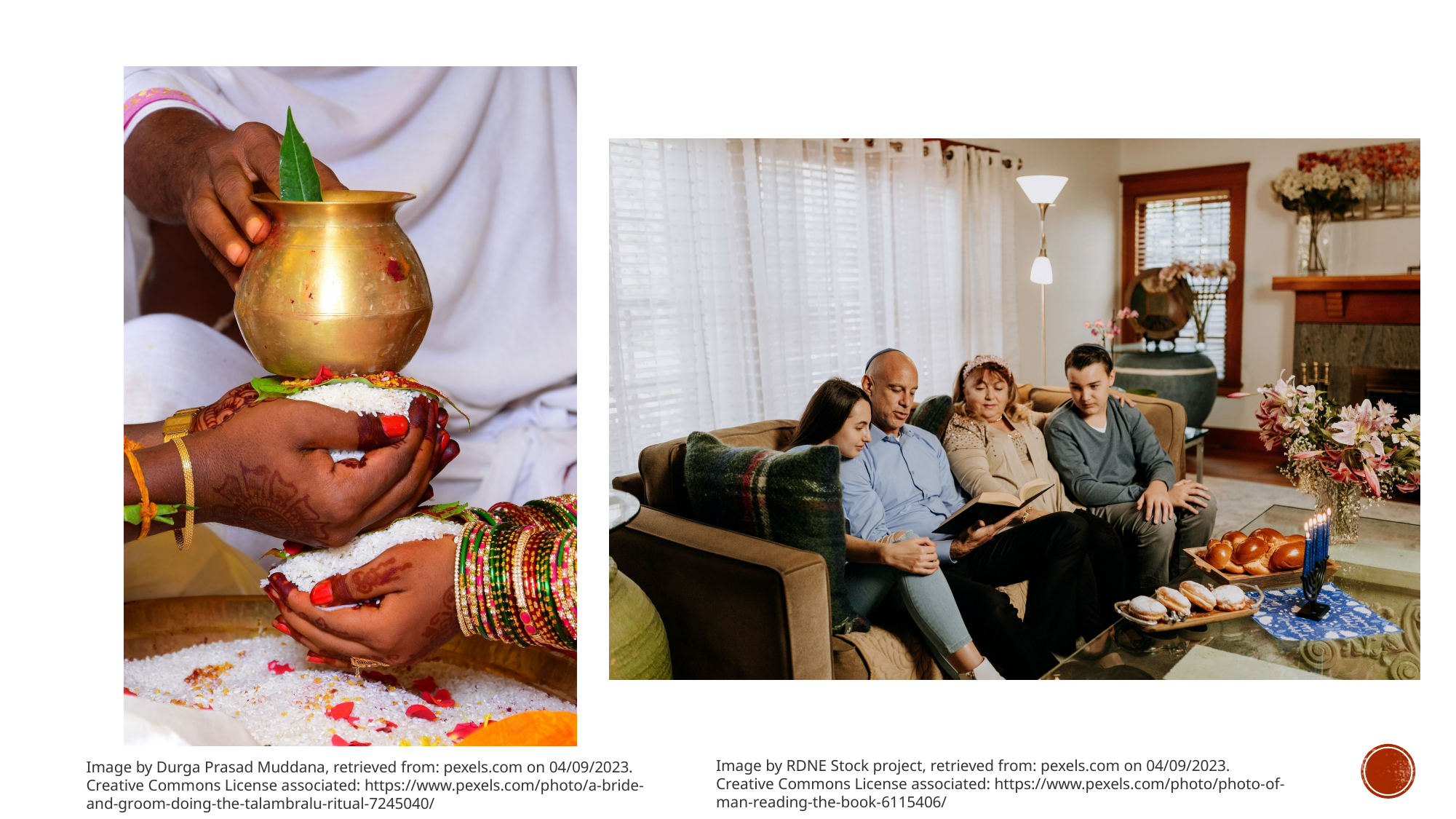

Image by RDNE Stock project, retrieved from: pexels.com on 04/09/2023. Creative Commons License associated: https://www.pexels.com/photo/photo-of-man-reading-the-book-6115406/
Image by Durga Prasad Muddana, retrieved from: pexels.com on 04/09/2023. Creative Commons License associated: https://www.pexels.com/photo/a-bride-and-groom-doing-the-talambralu-ritual-7245040/

## Slide 16
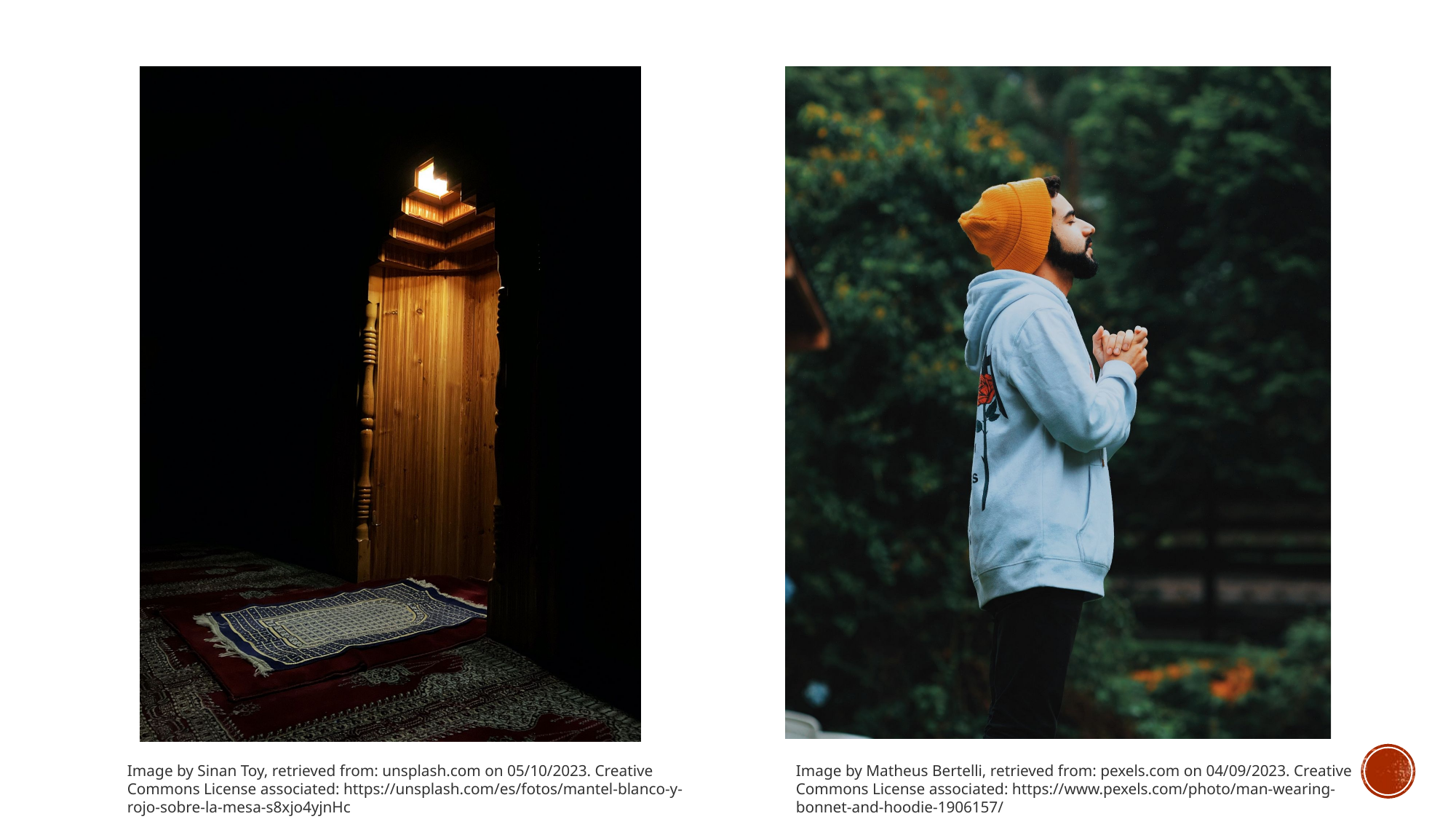

Image by Sinan Toy, retrieved from: unsplash.com on 05/10/2023. Creative Commons License associated: https://unsplash.com/es/fotos/mantel-blanco-y-rojo-sobre-la-mesa-s8xjo4yjnHc
Image by Matheus Bertelli, retrieved from: pexels.com on 04/09/2023. Creative Commons License associated: https://www.pexels.com/photo/man-wearing-bonnet-and-hoodie-1906157/

## Slide 17
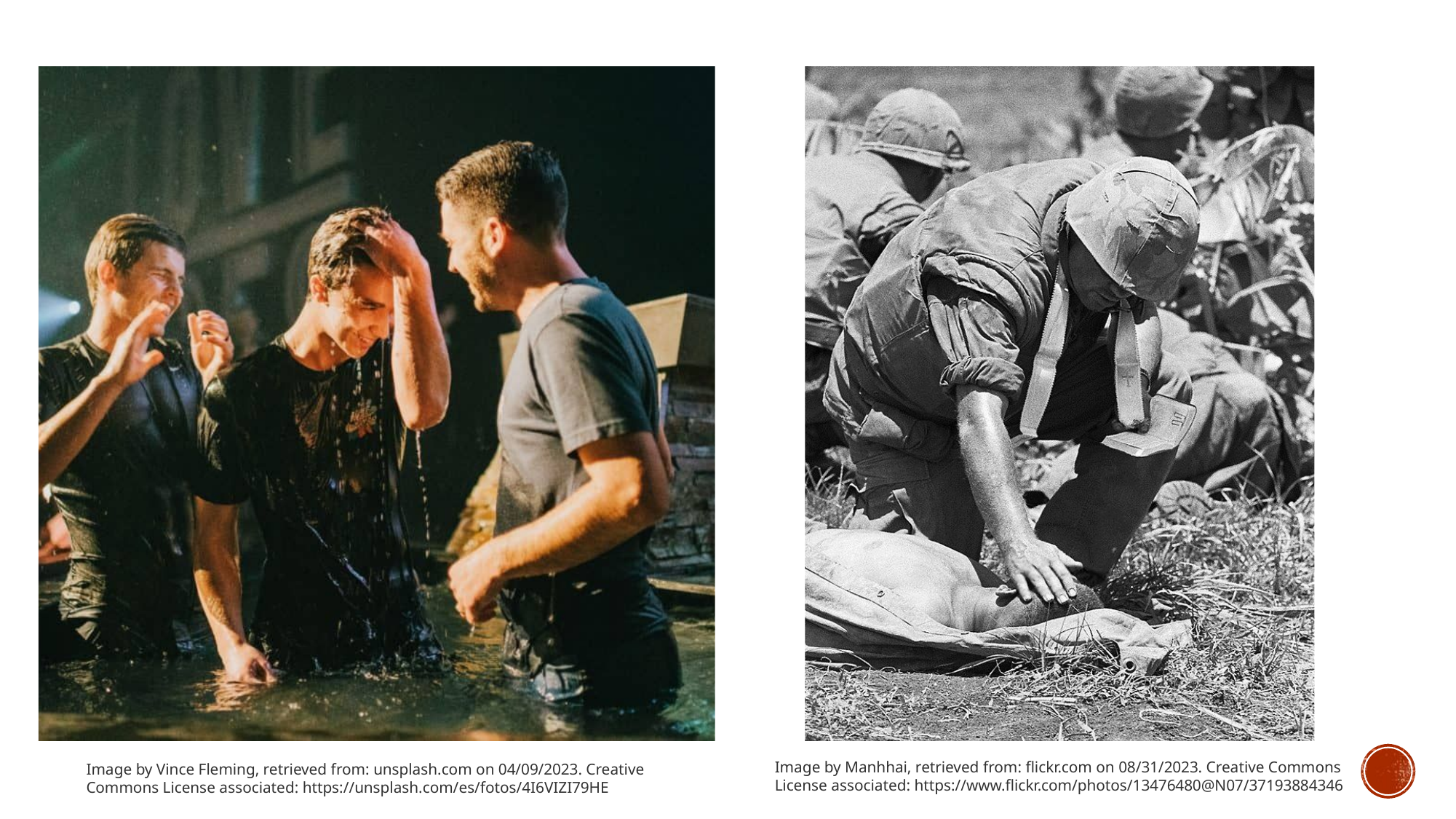

Image by Manhhai, retrieved from: flickr.com on 08/31/2023. Creative Commons License associated: https://www.flickr.com/photos/13476480@N07/37193884346
Image by Vince Fleming, retrieved from: unsplash.com on 04/09/2023. Creative Commons License associated: https://unsplash.com/es/fotos/4I6VIZI79HE

## Slide 18
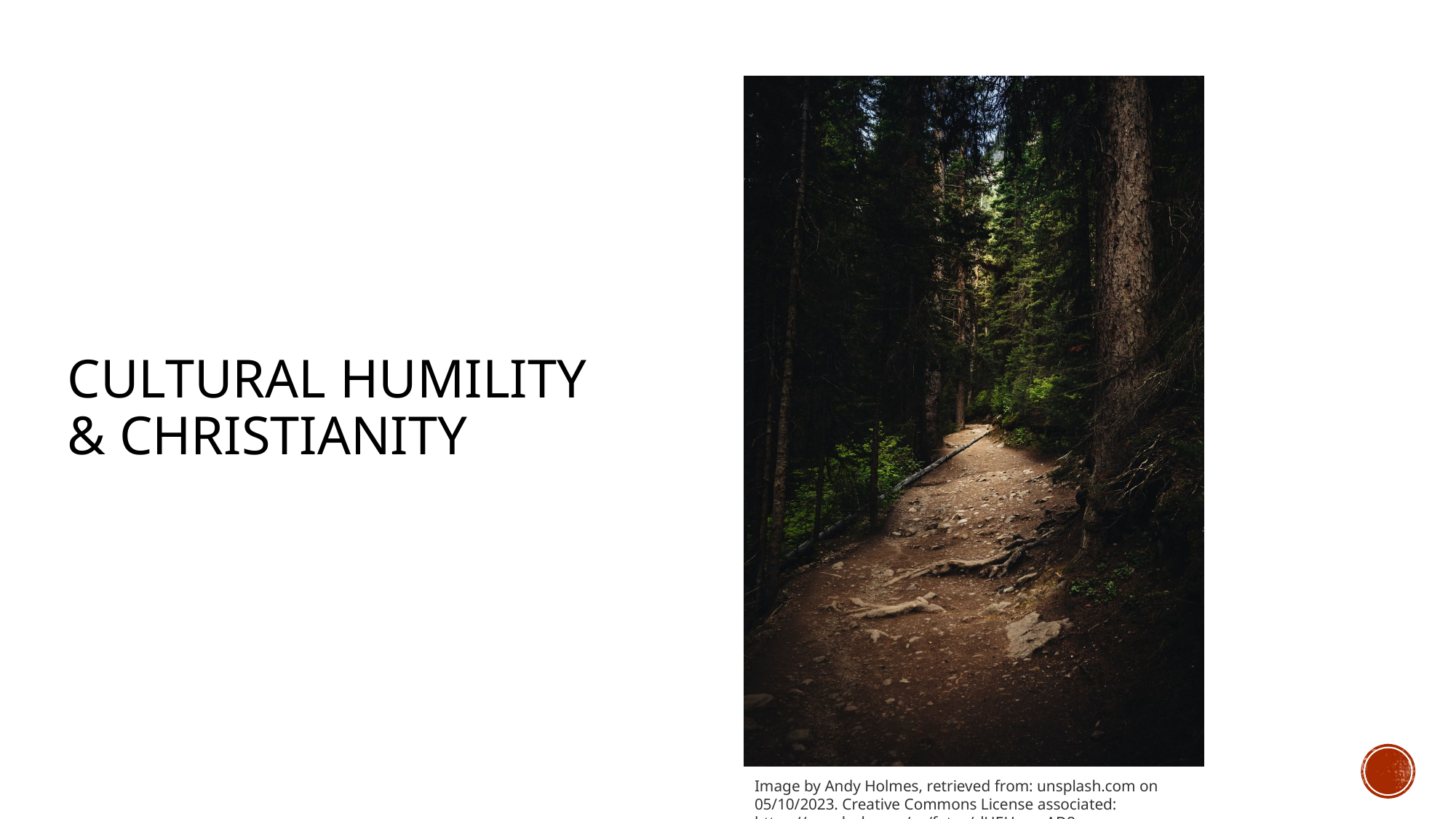

# Cultural humility & Christianity
Image by Andy Holmes, retrieved from: unsplash.com on 05/10/2023. Creative Commons License associated: https://unsplash.com/es/fotos/rlHEHymgAD8

## Slide 19
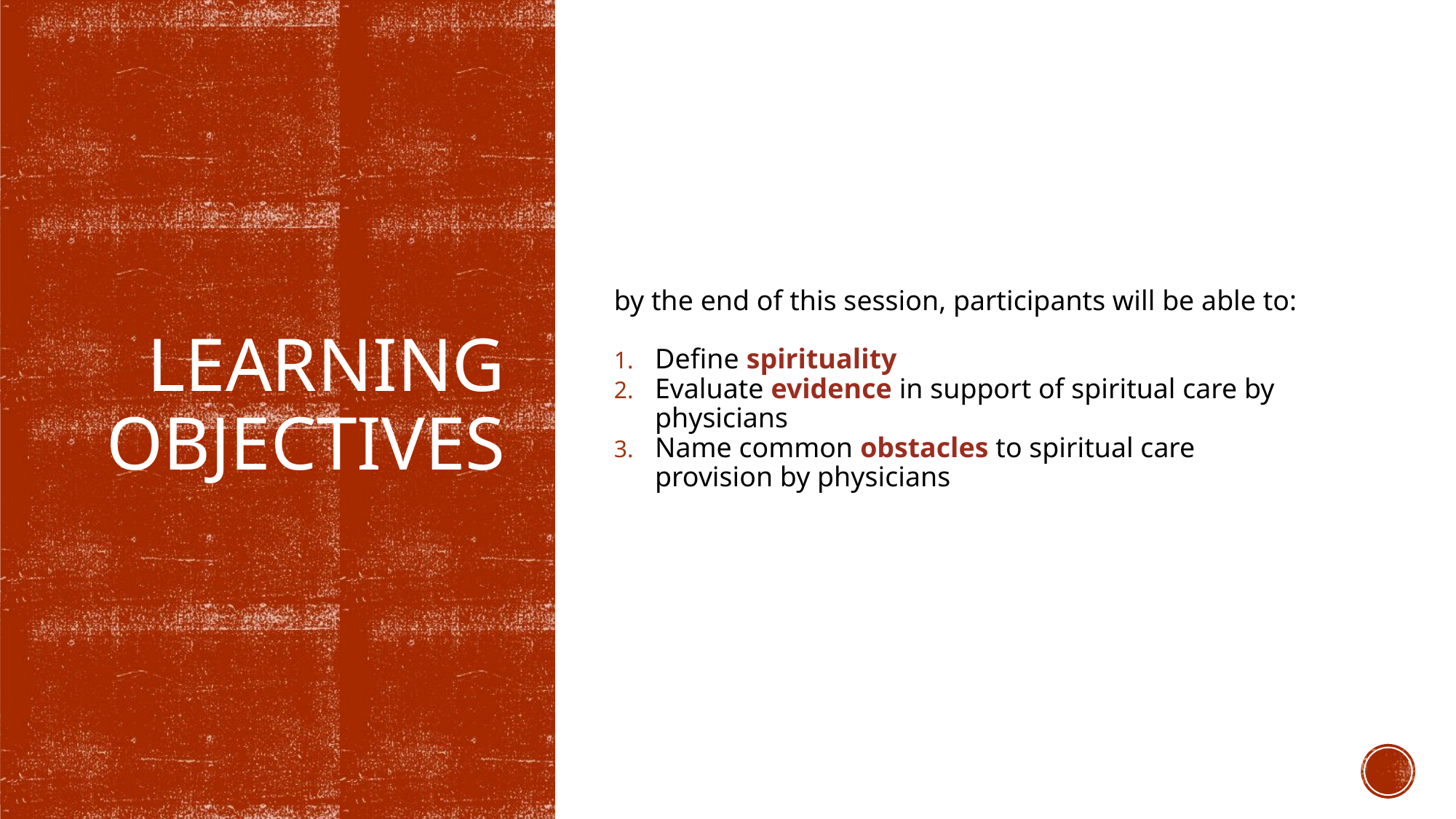

by the end of this session, participants will be able to:
Define spirituality
Evaluate evidence in support of spiritual care by physicians
Name common obstacles to spiritual care provision by physicians
# Learning Objectives

## Slide 20
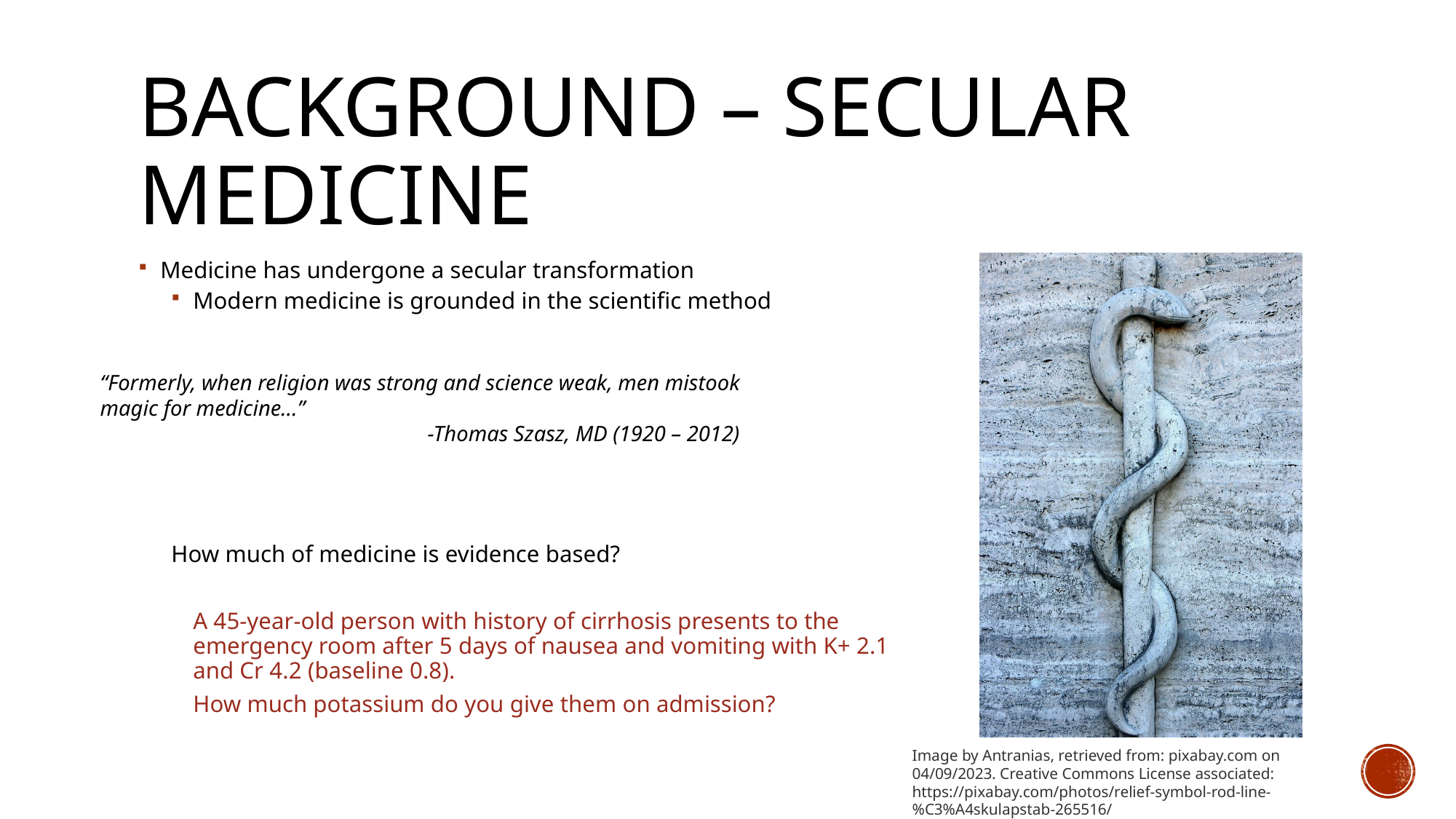

# Background – Secular Medicine
Medicine has undergone a secular transformation
Modern medicine is grounded in the scientific method
How much of medicine is evidence based?
A 45-year-old person with history of cirrhosis presents to the emergency room after 5 days of nausea and vomiting with K+ 2.1 and Cr 4.2 (baseline 0.8).
How much potassium do you give them on admission?
“Formerly, when religion was strong and science weak, men mistook magic for medicine…”
			-Thomas Szasz, MD (1920 – 2012)
Image by Antranias, retrieved from: pixabay.com on 04/09/2023. Creative Commons License associated: https://pixabay.com/photos/relief-symbol-rod-line-%C3%A4skulapstab-265516/

## Slide 21
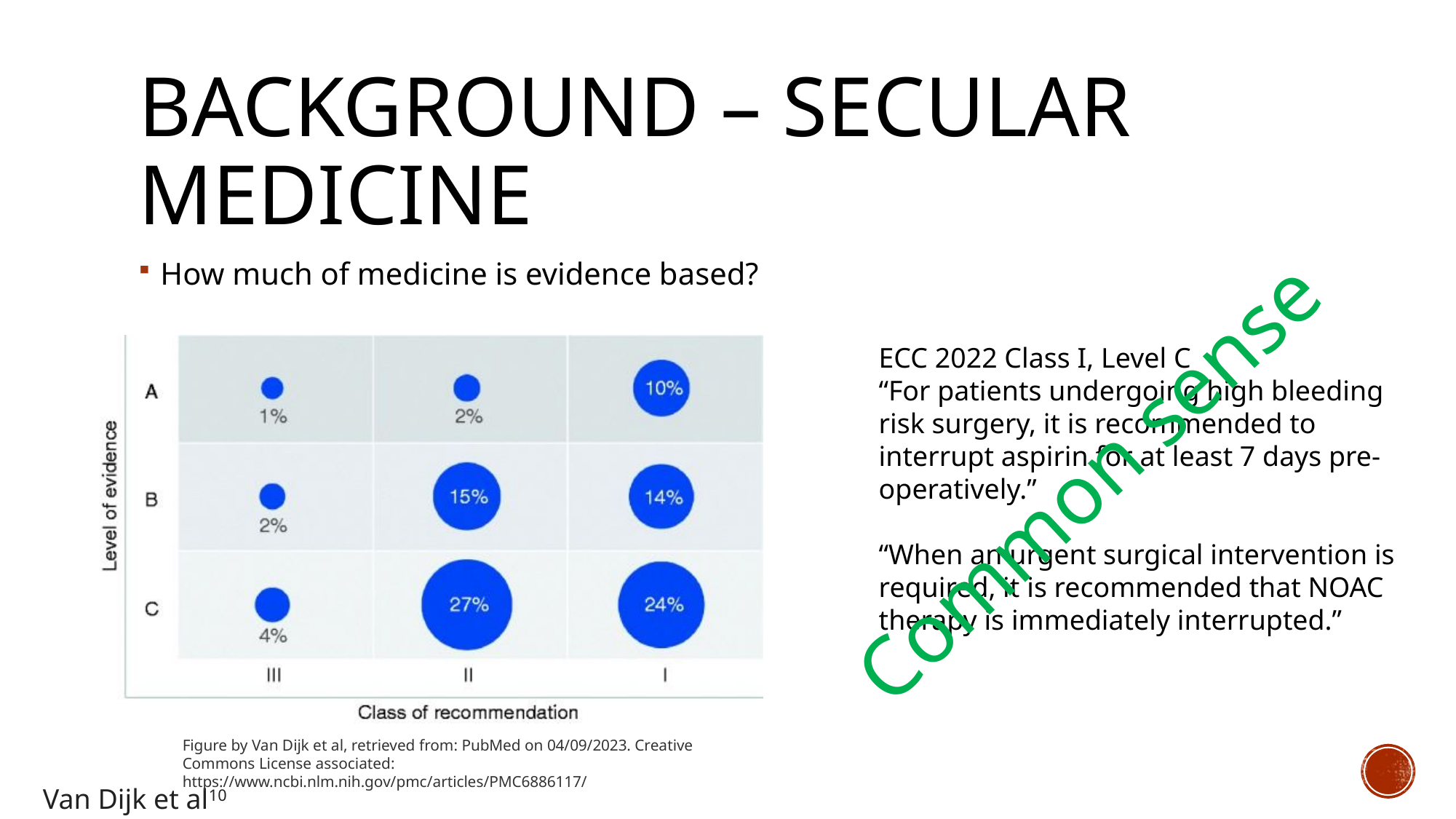

# Background – Secular Medicine
How much of medicine is evidence based?
ECC 2022 Class I, Level C
“For patients undergoing high bleeding risk surgery, it is recommended to interrupt aspirin for at least 7 days pre-operatively.”
“When an urgent surgical intervention is required, it is recommended that NOAC therapy is immediately interrupted.”
Common sense
Figure by Van Dijk et al, retrieved from: PubMed on 04/09/2023. Creative Commons License associated: https://www.ncbi.nlm.nih.gov/pmc/articles/PMC6886117/
Van Dijk et al10

## Slide 22
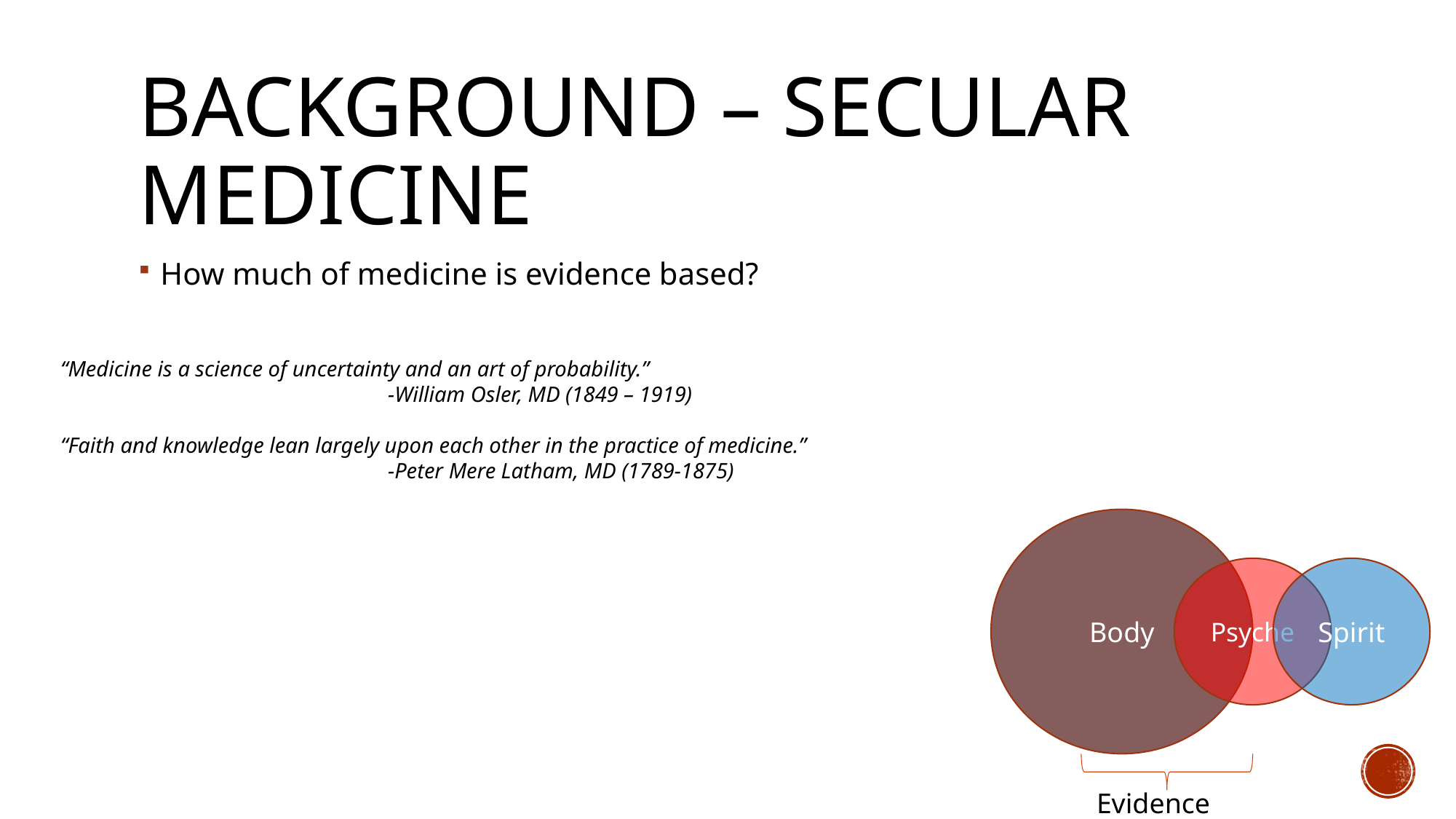

# Background – Secular Medicine
How much of medicine is evidence based?
“Medicine is a science of uncertainty and an art of probability.”
			-William Osler, MD (1849 – 1919)
“Faith and knowledge lean largely upon each other in the practice of medicine.”
			-Peter Mere Latham, MD (1789-1875)
Body
Psyche
Spirit
Evidence

## Slide 23
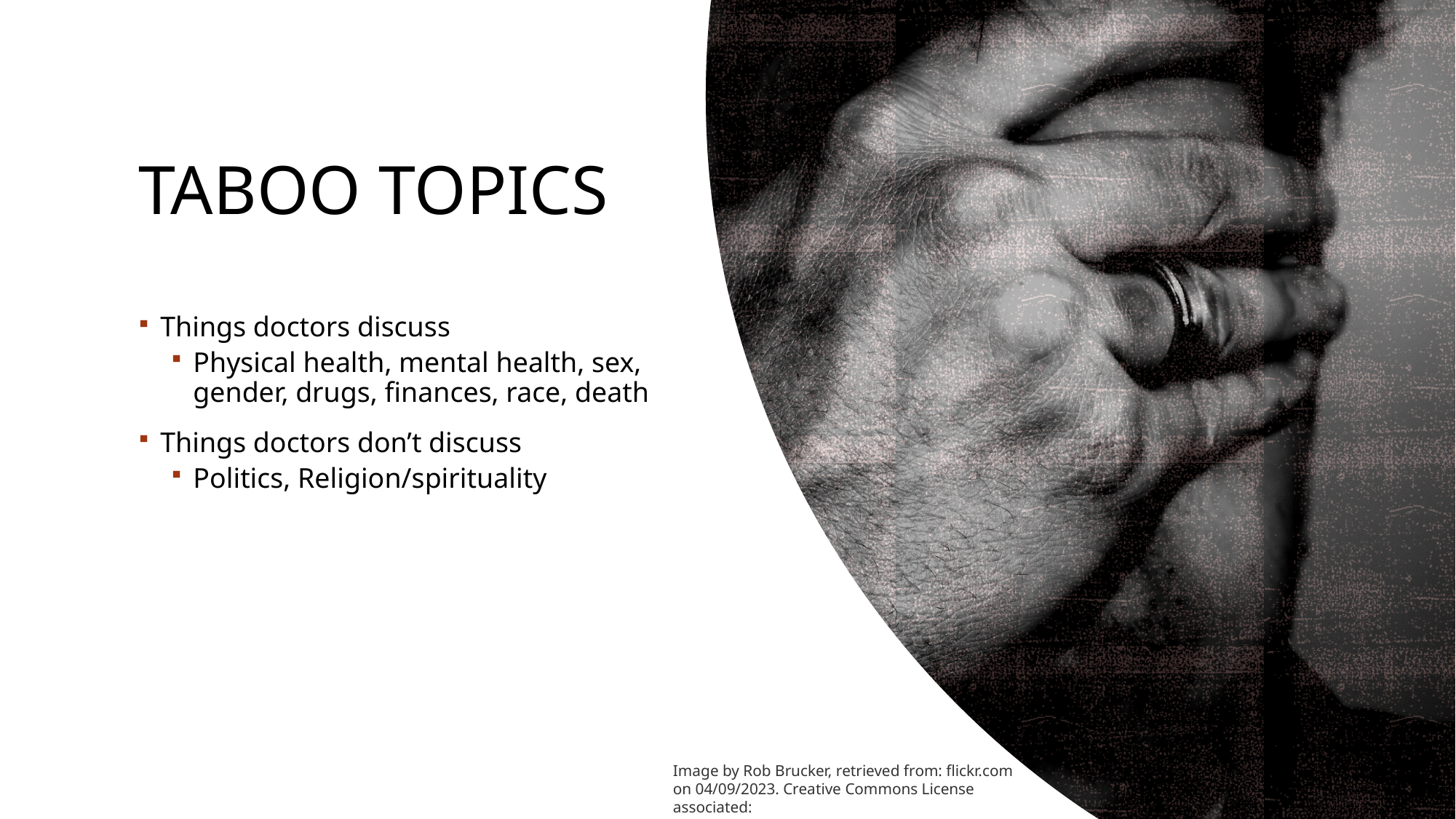

# Taboo topics
Things doctors discuss
Physical health, mental health, sex, gender, drugs, finances, race, death
Things doctors don’t discuss
Politics, Religion/spirituality
Image by Rob Brucker, retrieved from: flickr.com on 04/09/2023. Creative Commons License associated: https://www.flickr.com/photos/robbrucker/407842334

## Slide 24
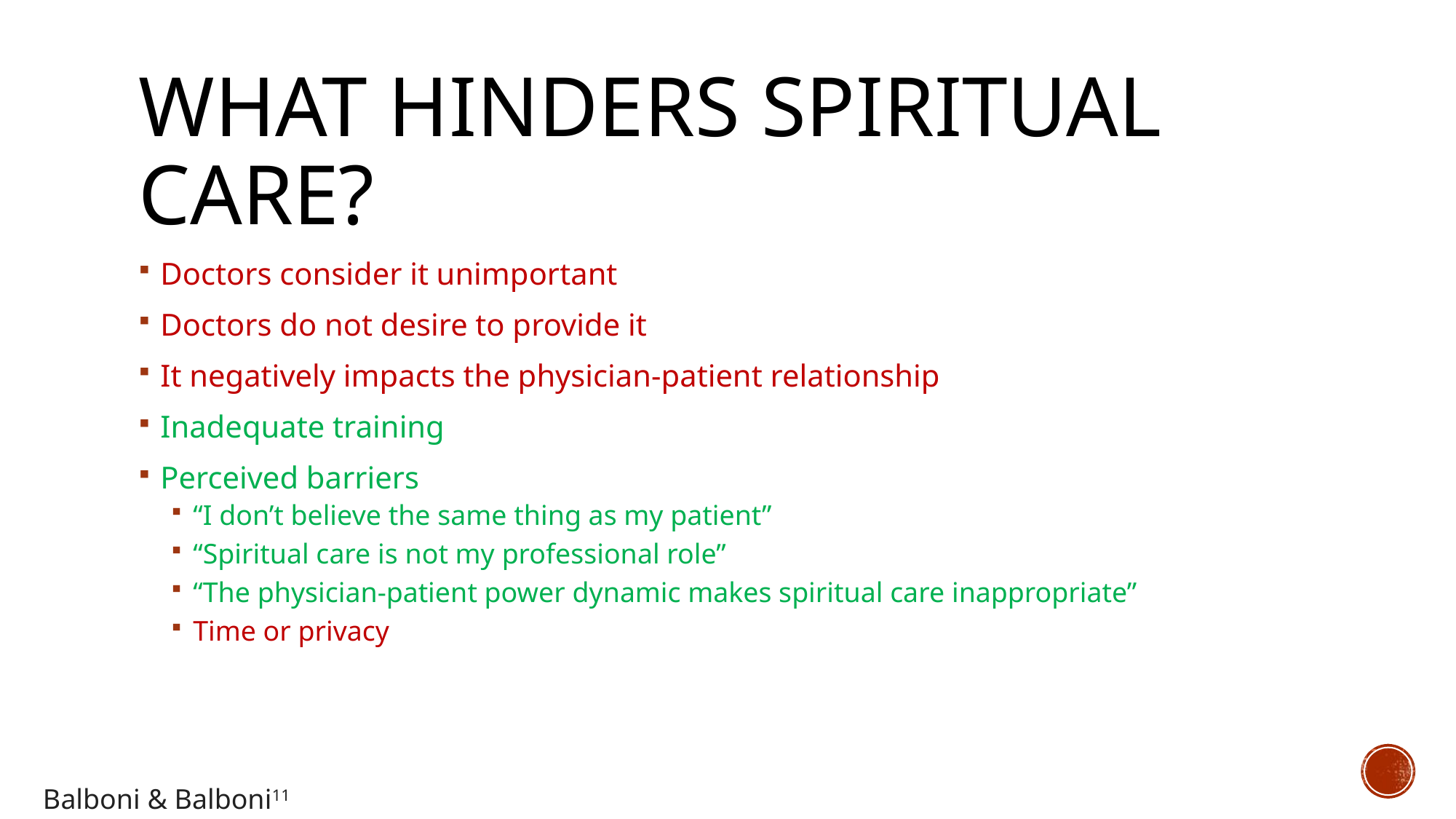

# What hinders spiritual care?
Doctors consider it unimportant
Doctors do not desire to provide it
It negatively impacts the physician-patient relationship
Inadequate training
Perceived barriers
“I don’t believe the same thing as my patient”
“Spiritual care is not my professional role”
“The physician-patient power dynamic makes spiritual care inappropriate”
Time or privacy
Balboni & Balboni11

## Slide 25
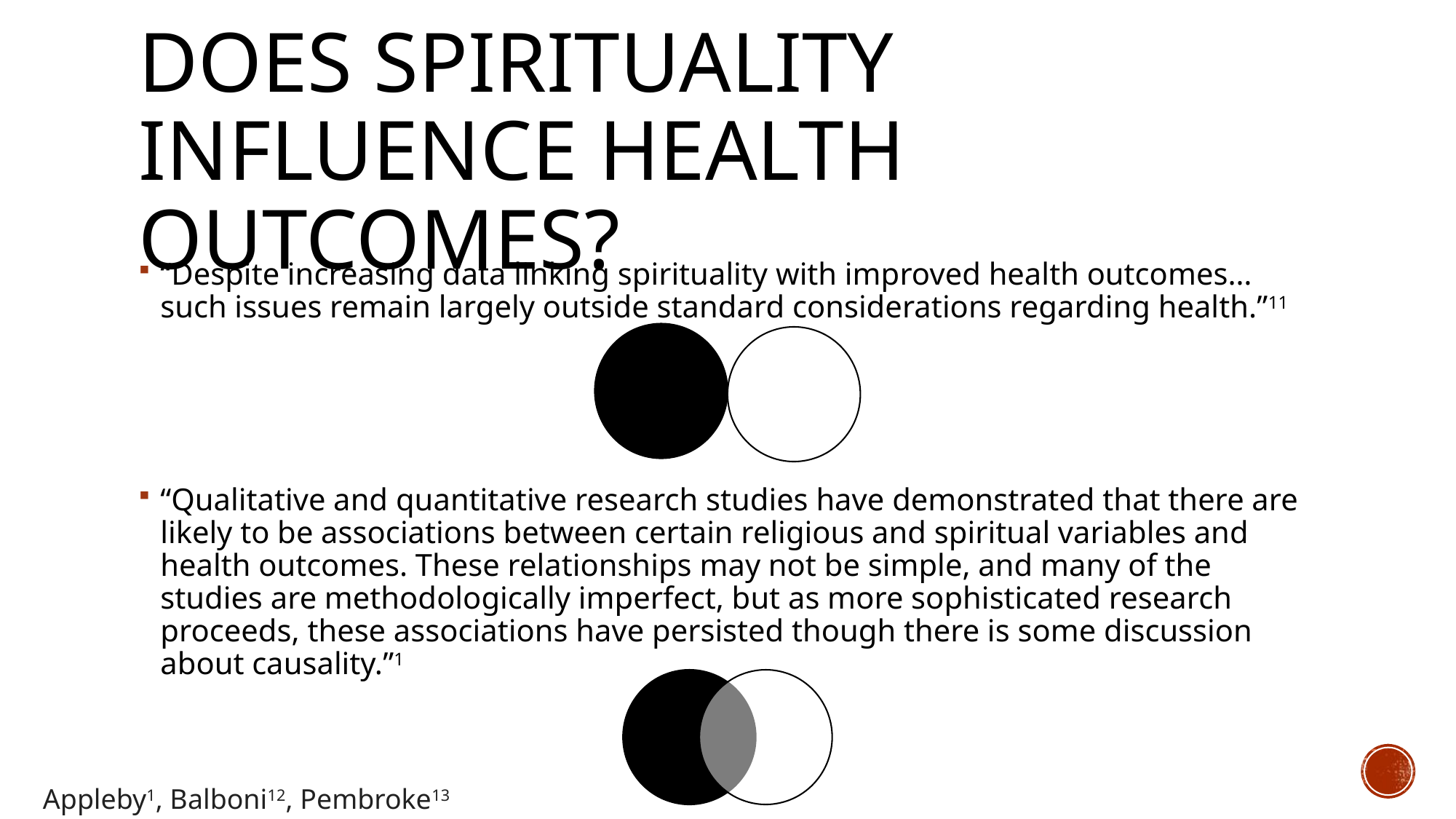

# Does Spirituality Influence Health Outcomes?
“Despite increasing data linking spirituality with improved health outcomes… such issues remain largely outside standard considerations regarding health.”11
“Qualitative and quantitative research studies have demonstrated that there are likely to be associations between certain religious and spiritual variables and health outcomes. These relationships may not be simple, and many of the studies are methodologically imperfect, but as more sophisticated research proceeds, these associations have persisted though there is some discussion about causality.”1
Appleby1, Balboni12, Pembroke13

## Slide 26
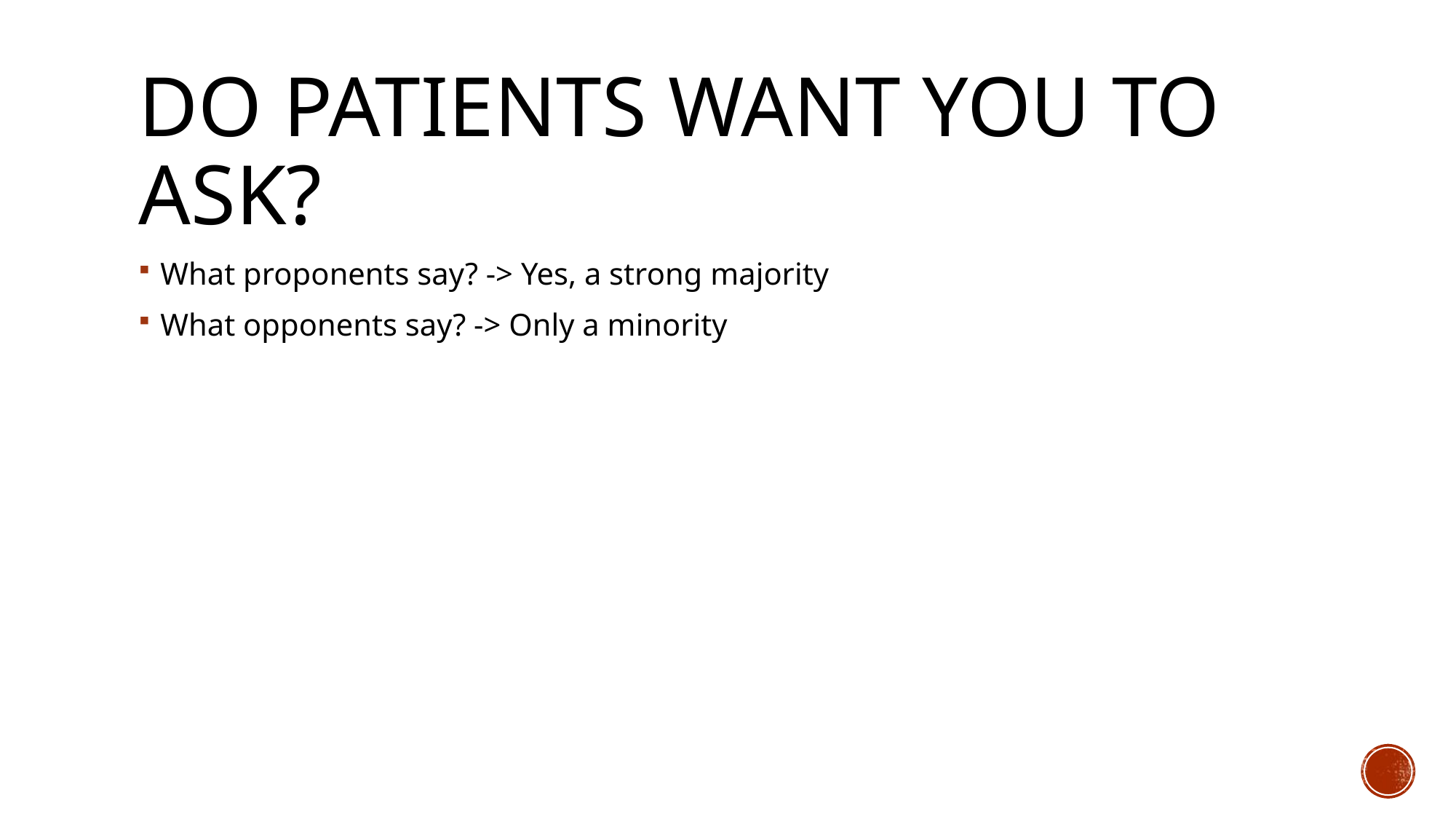

# Do patients want you to ask?
What proponents say? -> Yes, a strong majority
What opponents say? -> Only a minority

## Slide 27
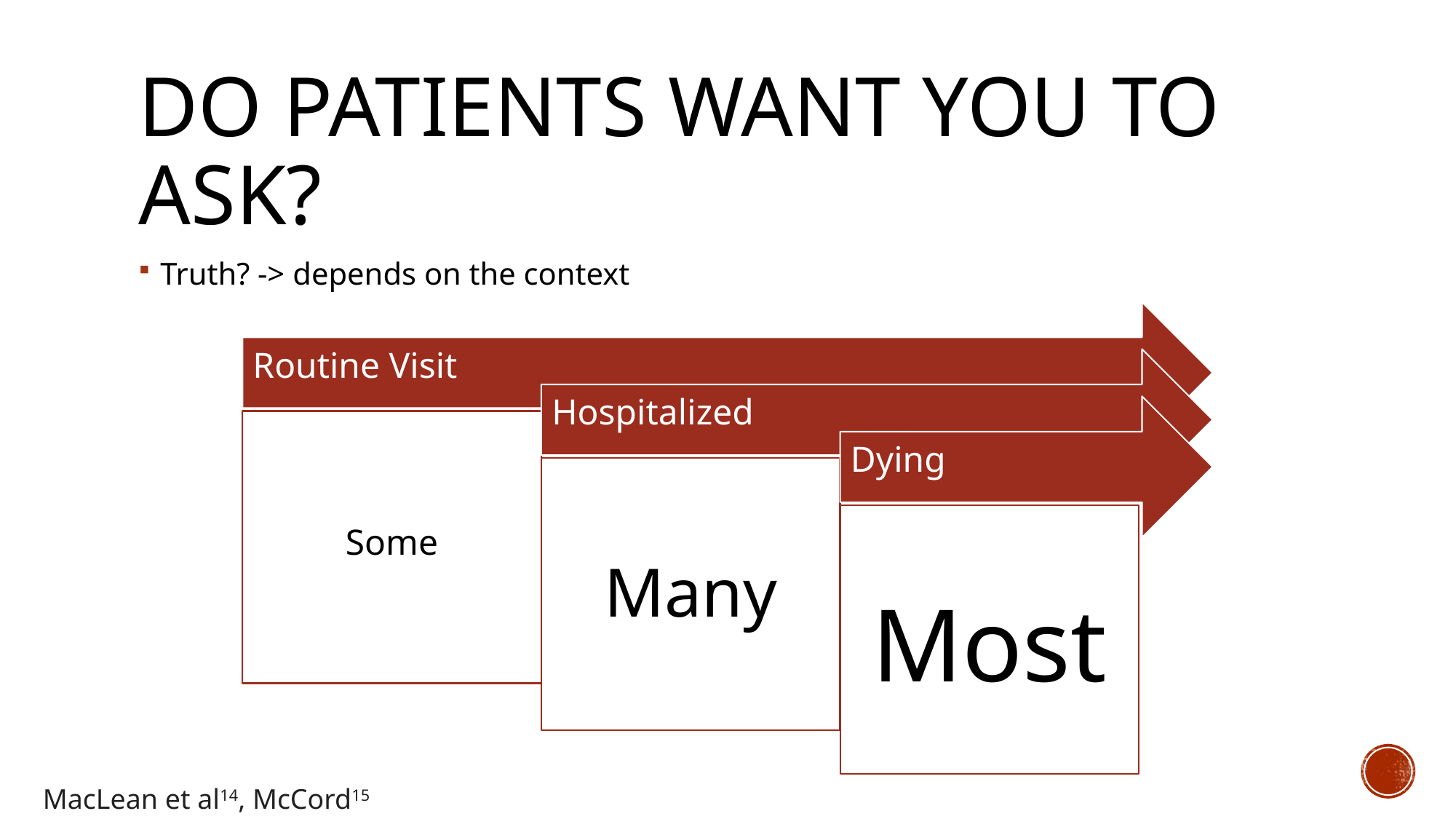

# Do patients want you to ask?
Truth? -> depends on the context
MacLean et al14, McCord15

## Slide 28
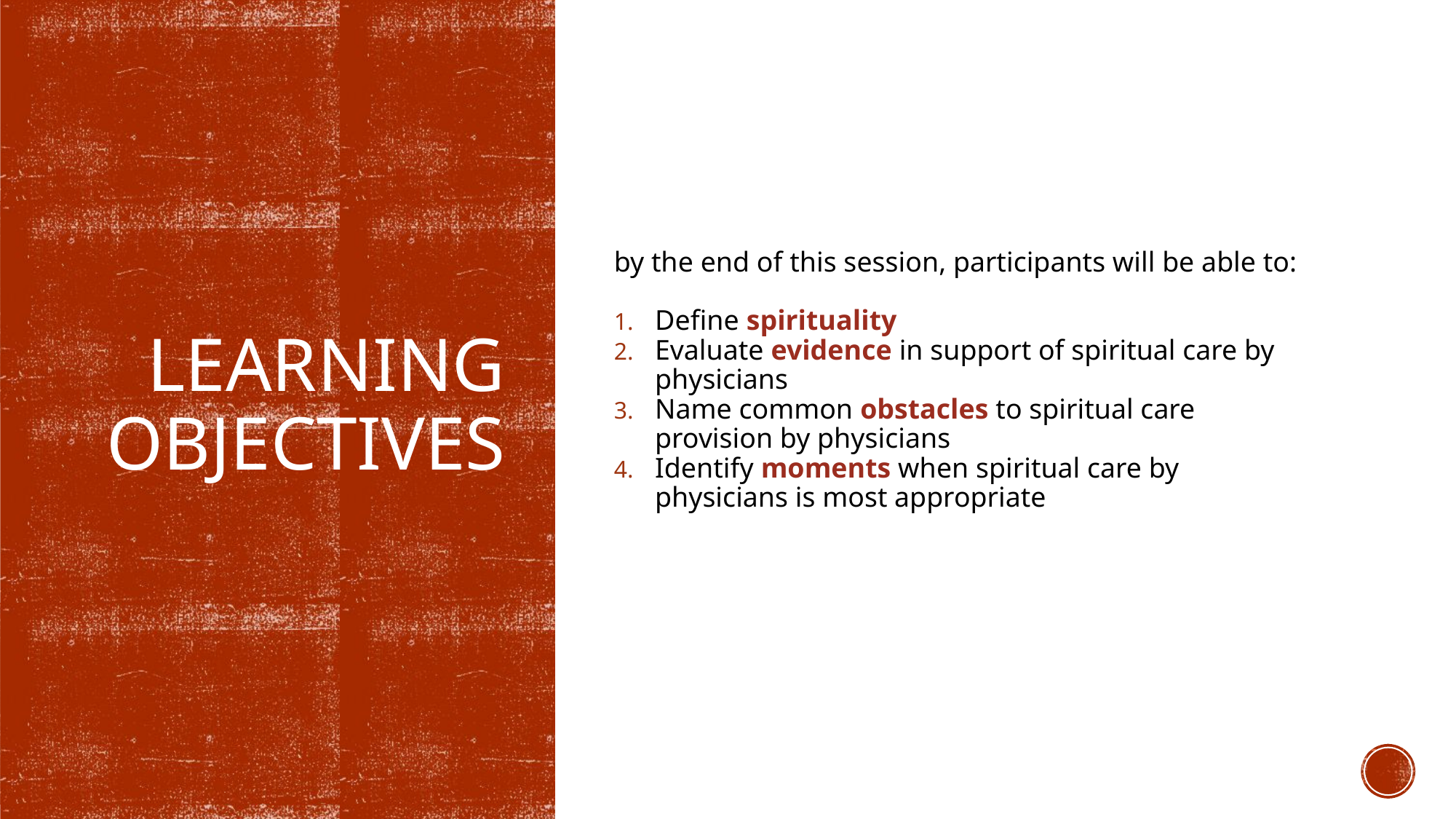

by the end of this session, participants will be able to:
Define spirituality
Evaluate evidence in support of spiritual care by physicians
Name common obstacles to spiritual care provision by physicians
Identify moments when spiritual care by physicians is most appropriate
# Learning Objectives

## Slide 29
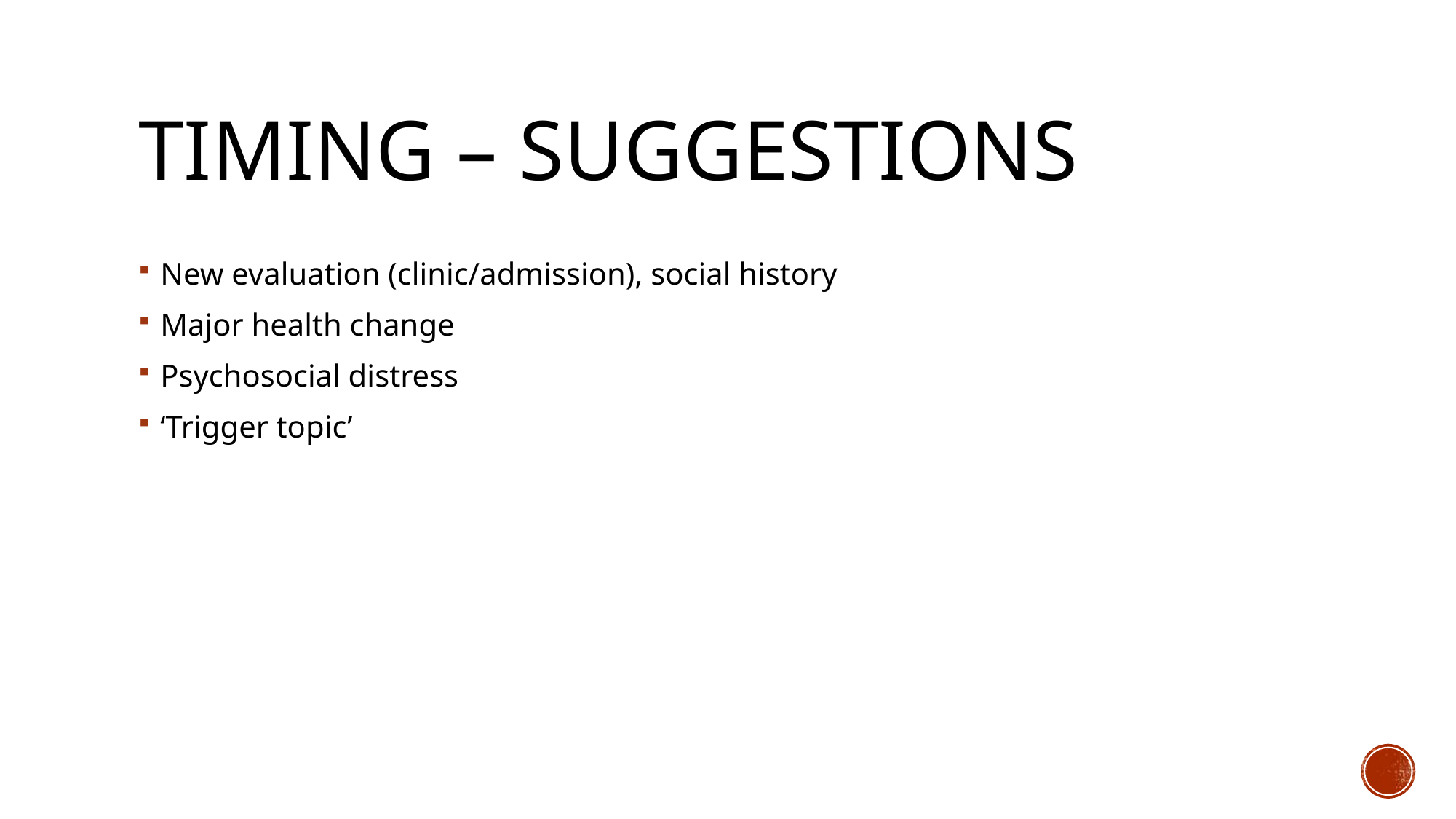

# Timing – Suggestions
New evaluation (clinic/admission), social history
Major health change
Psychosocial distress
‘Trigger topic’

## Slide 30
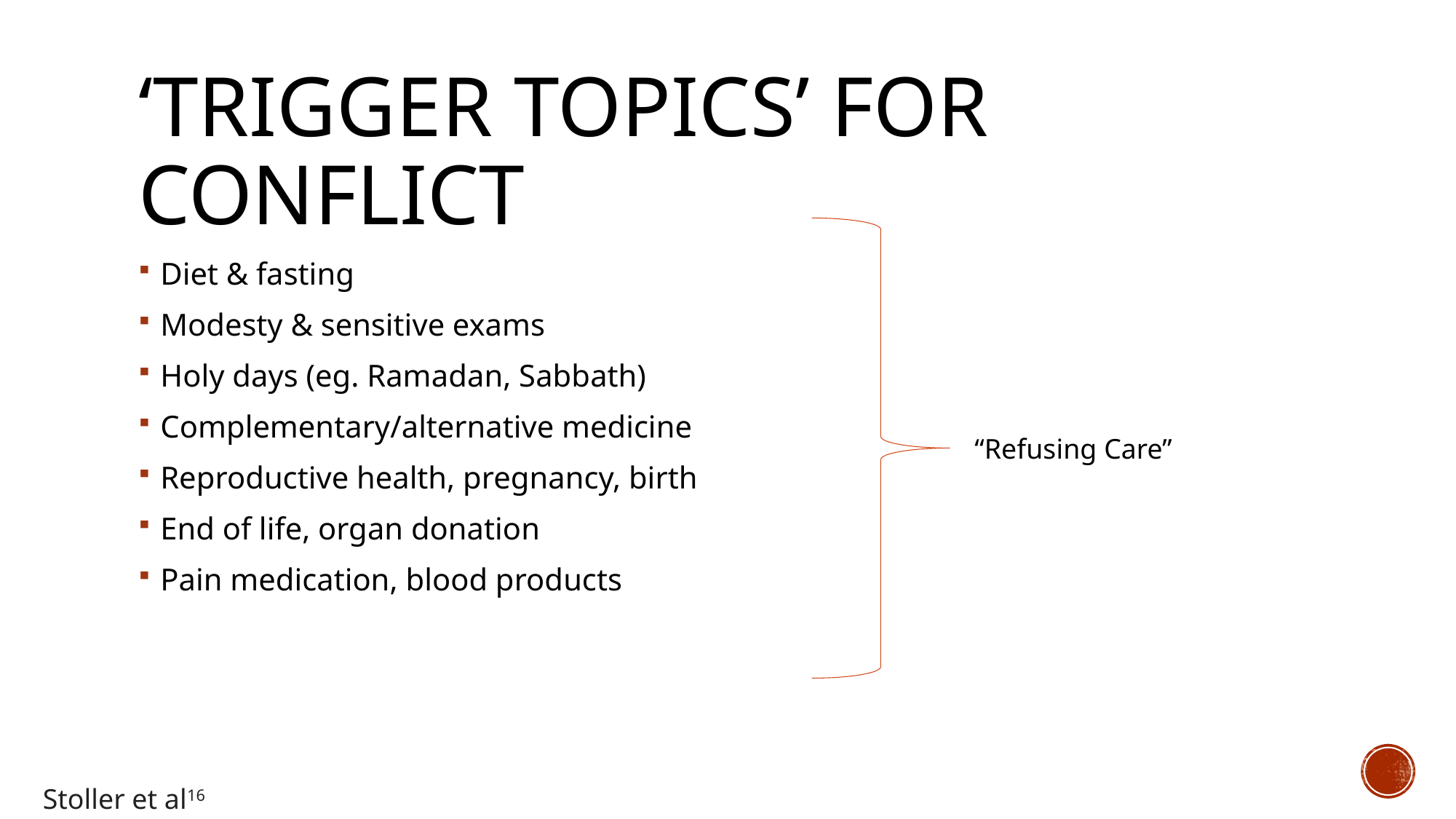

# ‘Trigger topics’ for conflict
Diet & fasting
Modesty & sensitive exams
Holy days (eg. Ramadan, Sabbath)
Complementary/alternative medicine
Reproductive health, pregnancy, birth
End of life, organ donation
Pain medication, blood products
“Refusing Care”
Stoller et al16

## Slide 31
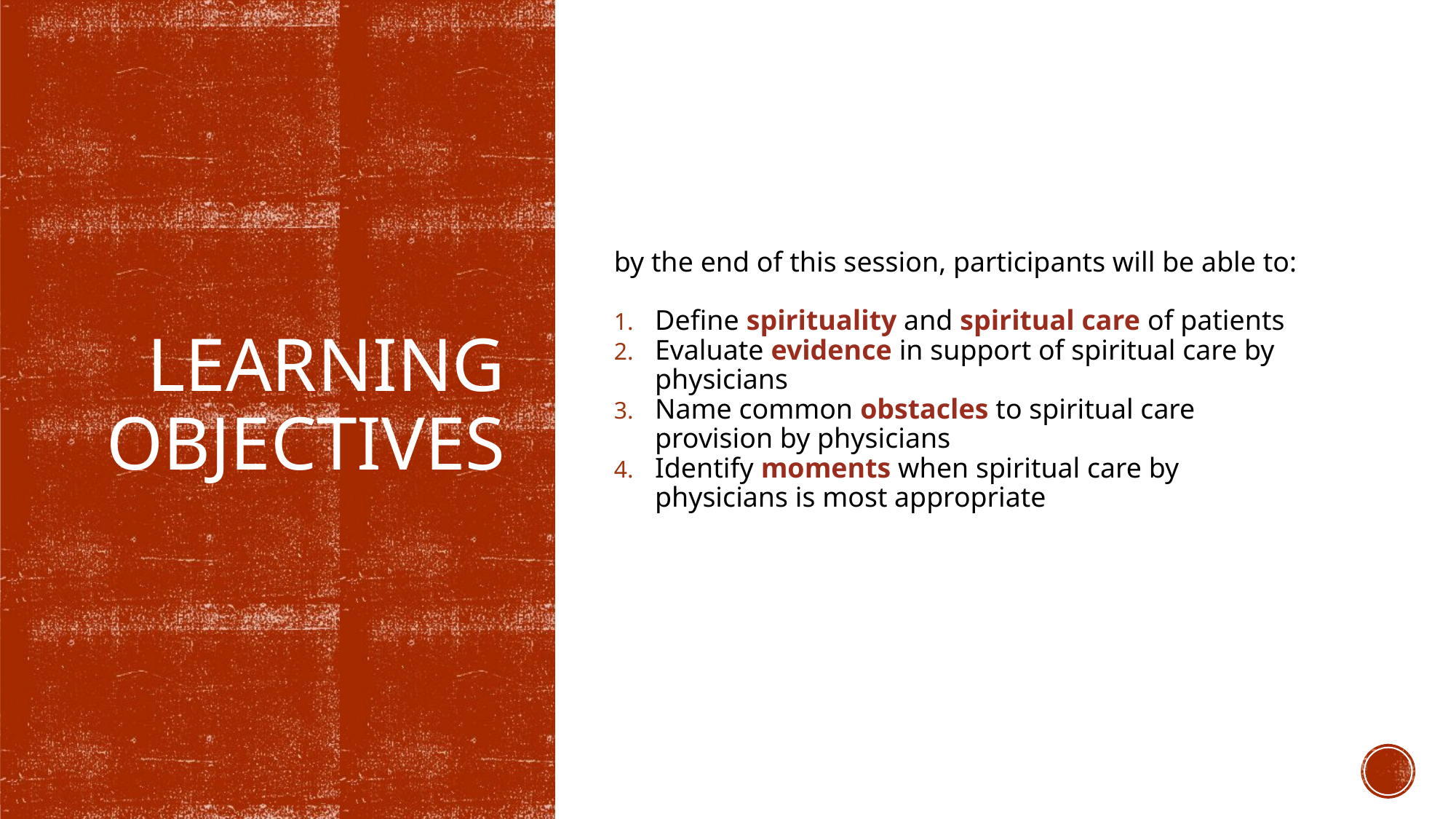

by the end of this session, participants will be able to:
Define spirituality and spiritual care of patients
Evaluate evidence in support of spiritual care by physicians
Name common obstacles to spiritual care provision by physicians
Identify moments when spiritual care by physicians is most appropriate
# Learning Objectives

## Slide 32
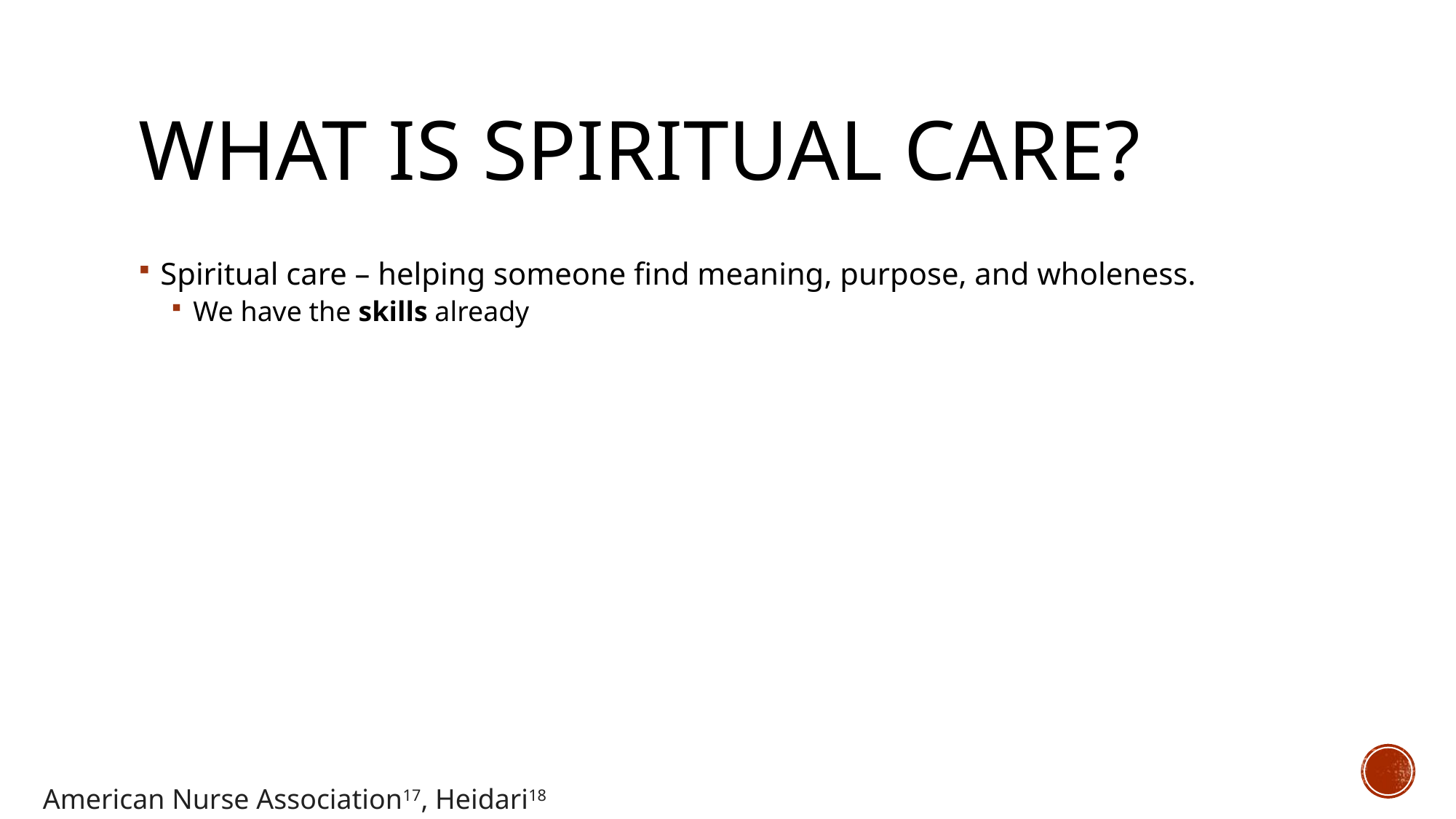

# What is Spiritual Care?
Spiritual care – helping someone find meaning, purpose, and wholeness.
We have the skills already
American Nurse Association17, Heidari18

## Slide 33
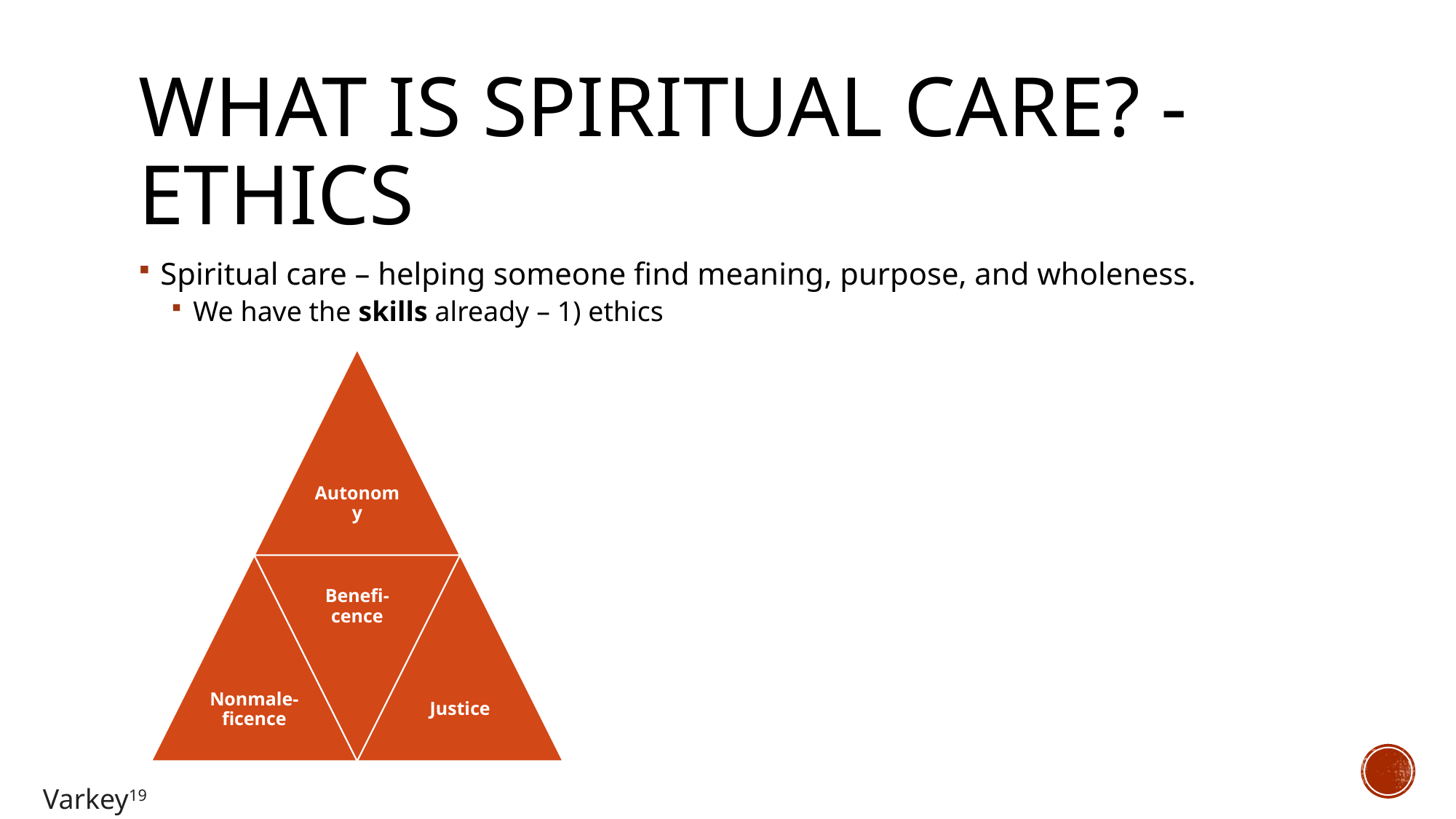

# What is Spiritual Care? - Ethics
Spiritual care – helping someone find meaning, purpose, and wholeness.
We have the skills already – 1) ethics
Varkey19

## Slide 34
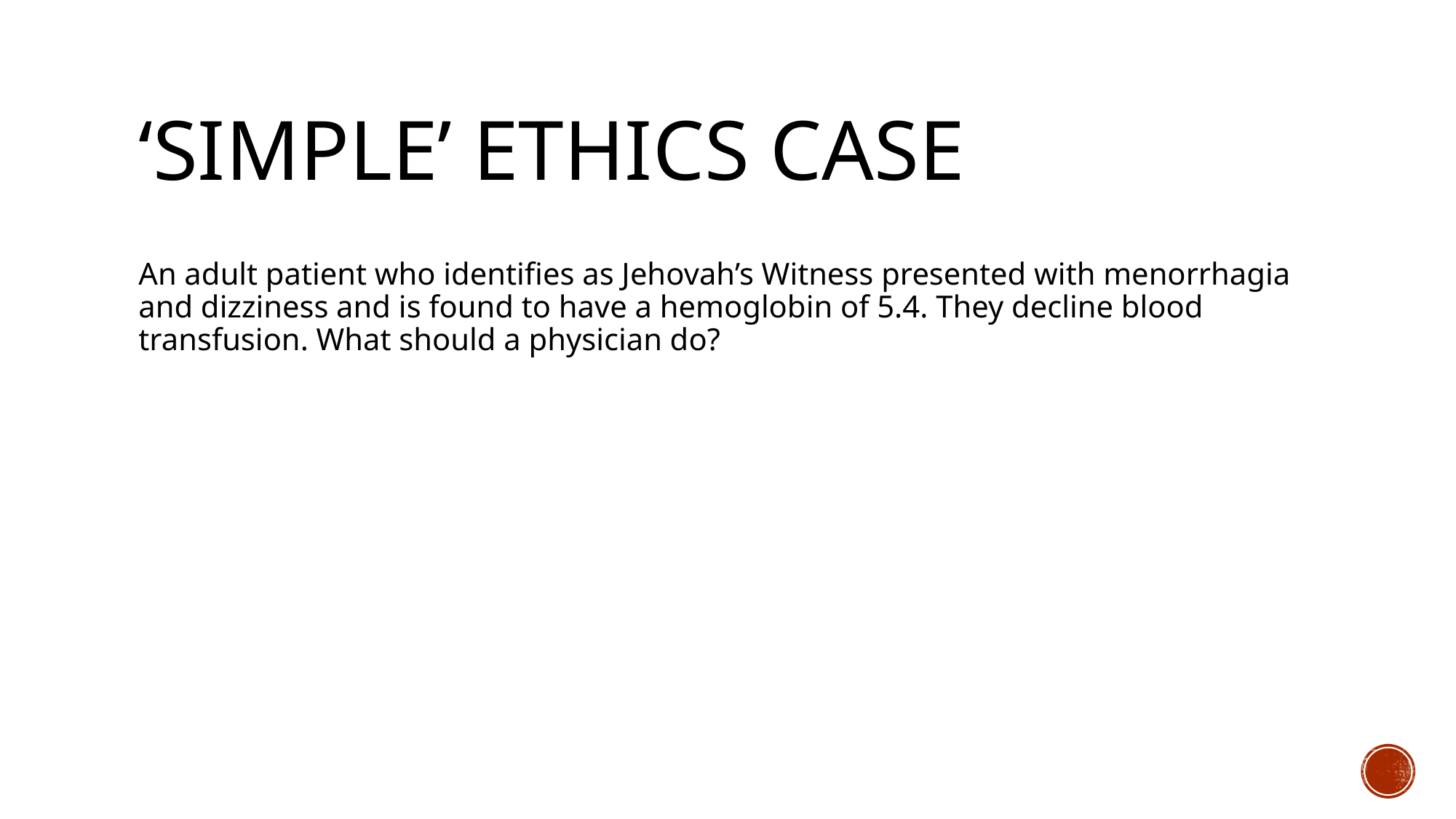

# ‘Simple’ Ethics Case
An adult patient who identifies as Jehovah’s Witness presented with menorrhagia and dizziness and is found to have a hemoglobin of 5.4. They decline blood transfusion. What should a physician do?

## Slide 35
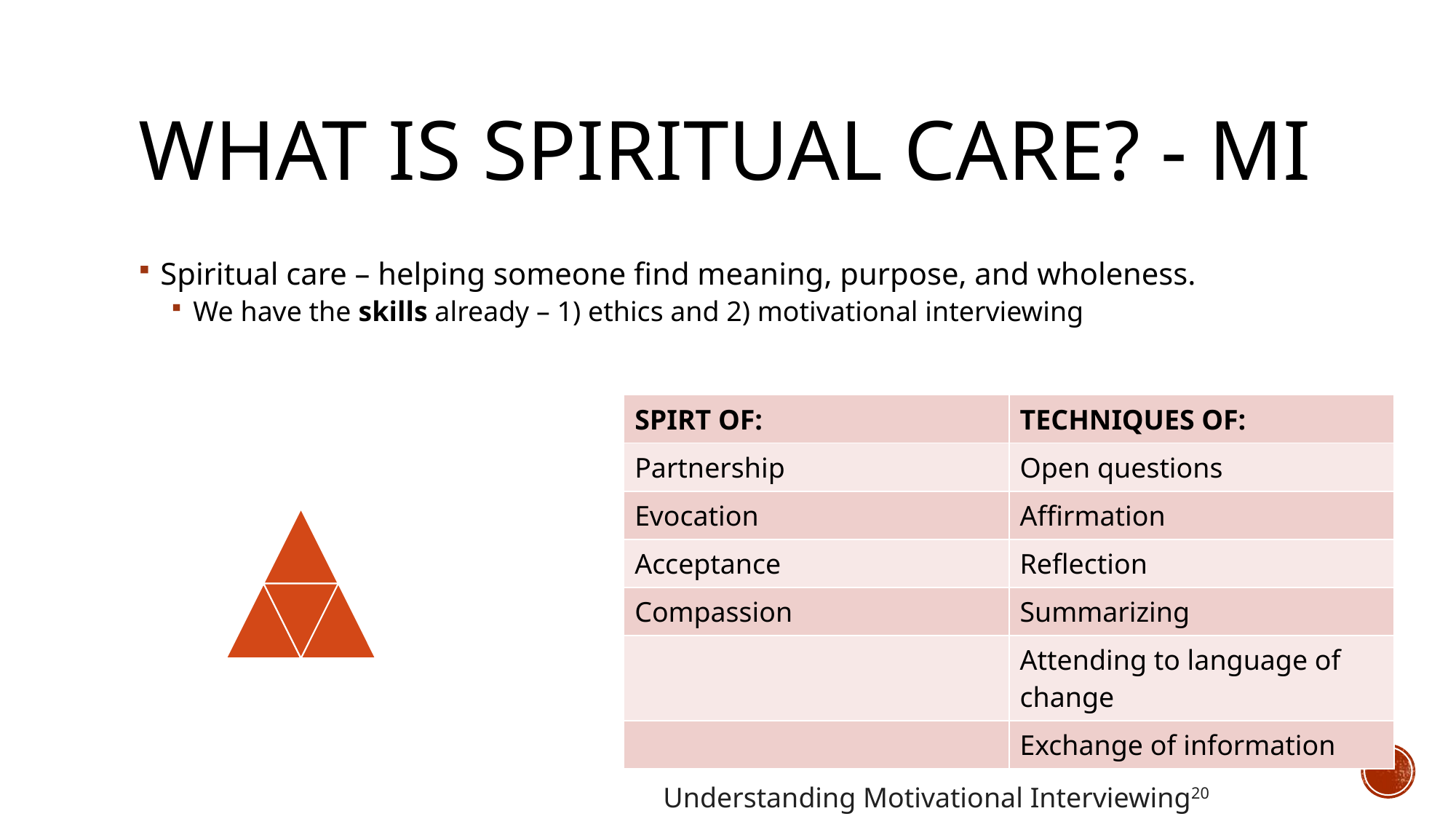

# What is Spiritual Care? - MI
Spiritual care – helping someone find meaning, purpose, and wholeness.
We have the skills already – 1) ethics and 2) motivational interviewing
| SPIRT OF: | TECHNIQUES OF: |
| --- | --- |
| Partnership | Open questions |
| Evocation | Affirmation |
| Acceptance | Reflection |
| Compassion | Summarizing |
| | Attending to language of change |
| | Exchange of information |
Understanding Motivational Interviewing20

## Slide 36
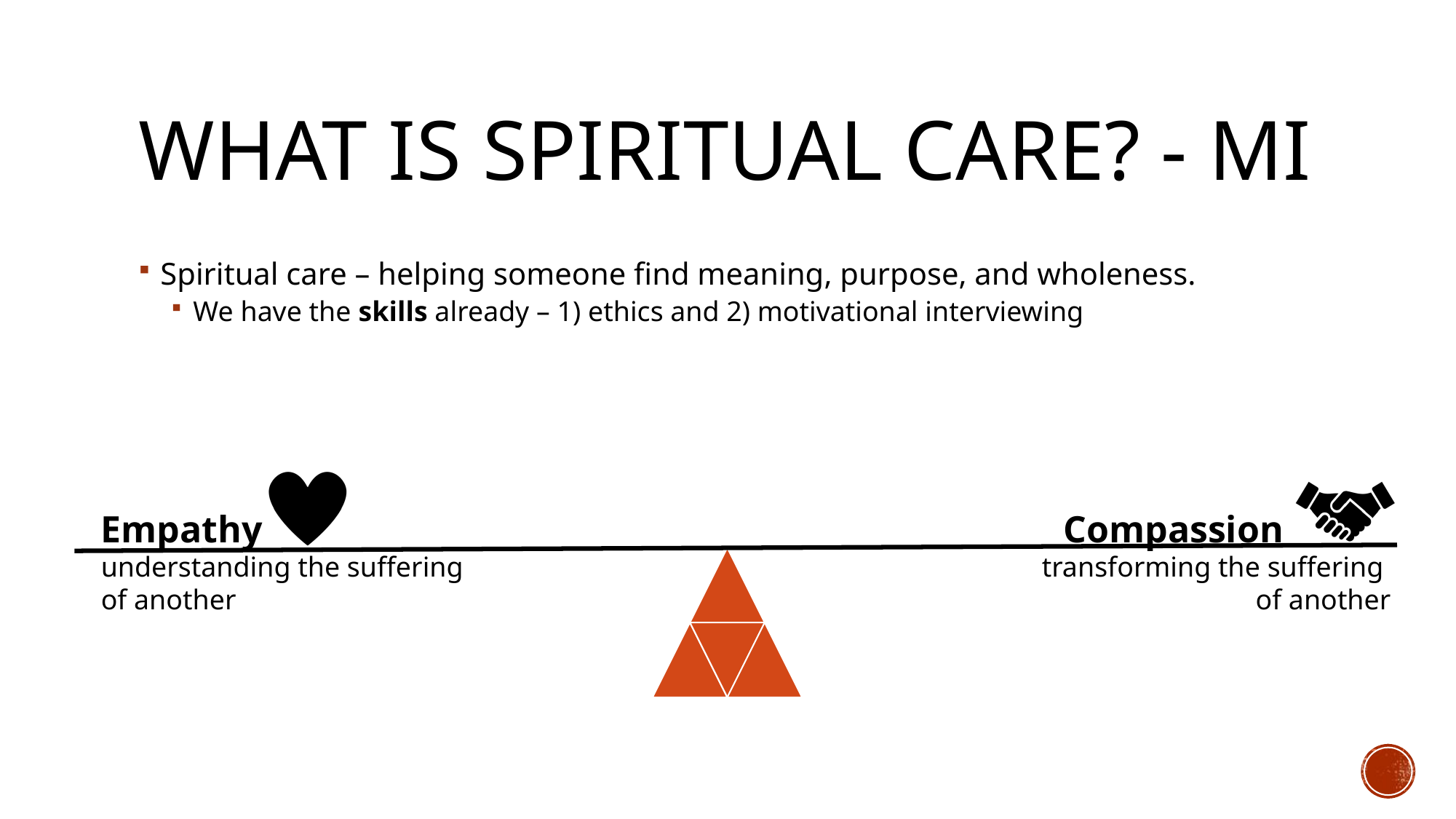

# What is Spiritual Care? - MI
Spiritual care – helping someone find meaning, purpose, and wholeness.
We have the skills already – 1) ethics and 2) motivational interviewing
Empathy
understanding the suffering
of another
Compassion
transforming the suffering
of another

## Slide 37
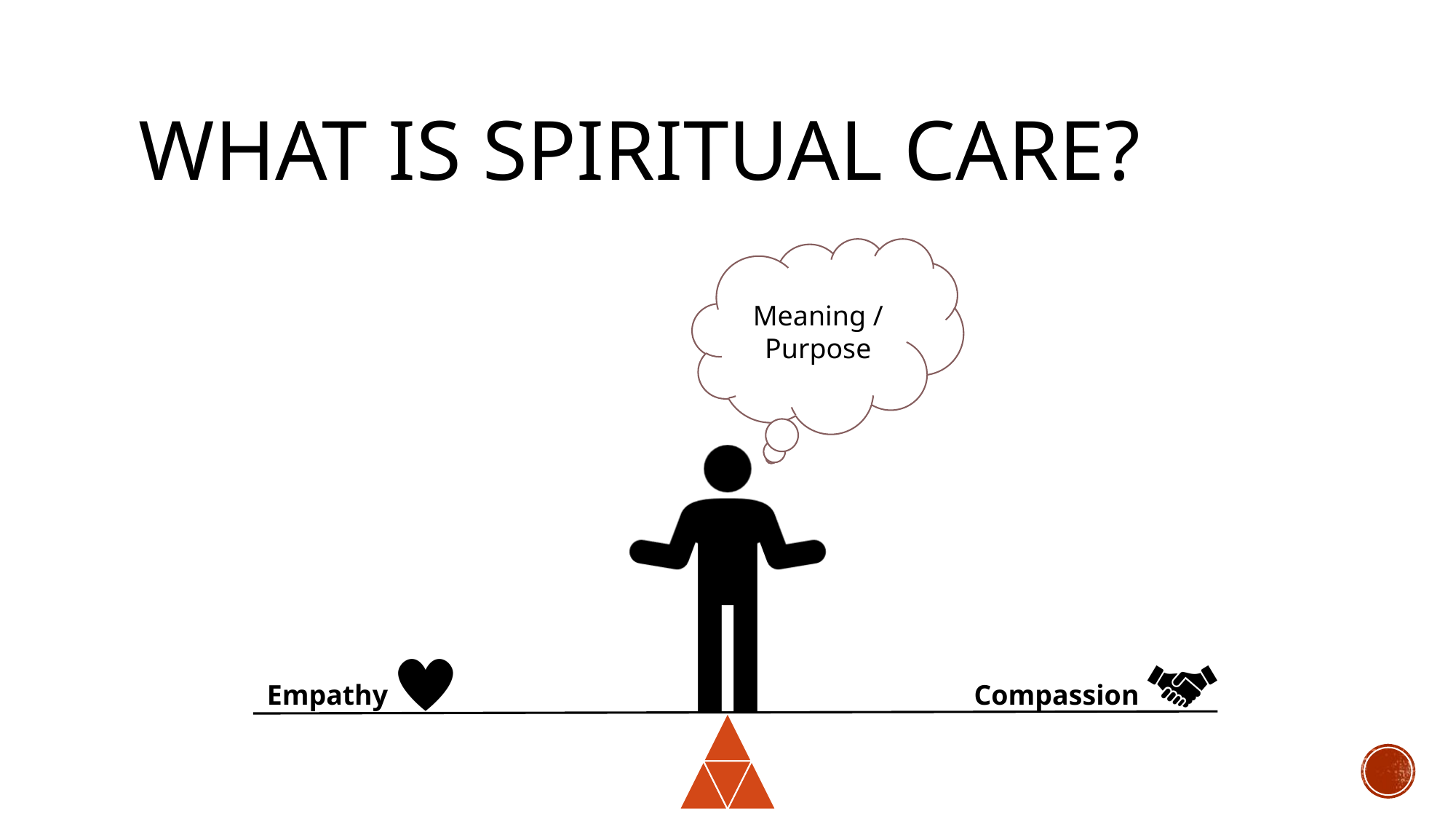

# What is Spiritual Care?
Meaning / Purpose
Empathy
Compassion

## Slide 38
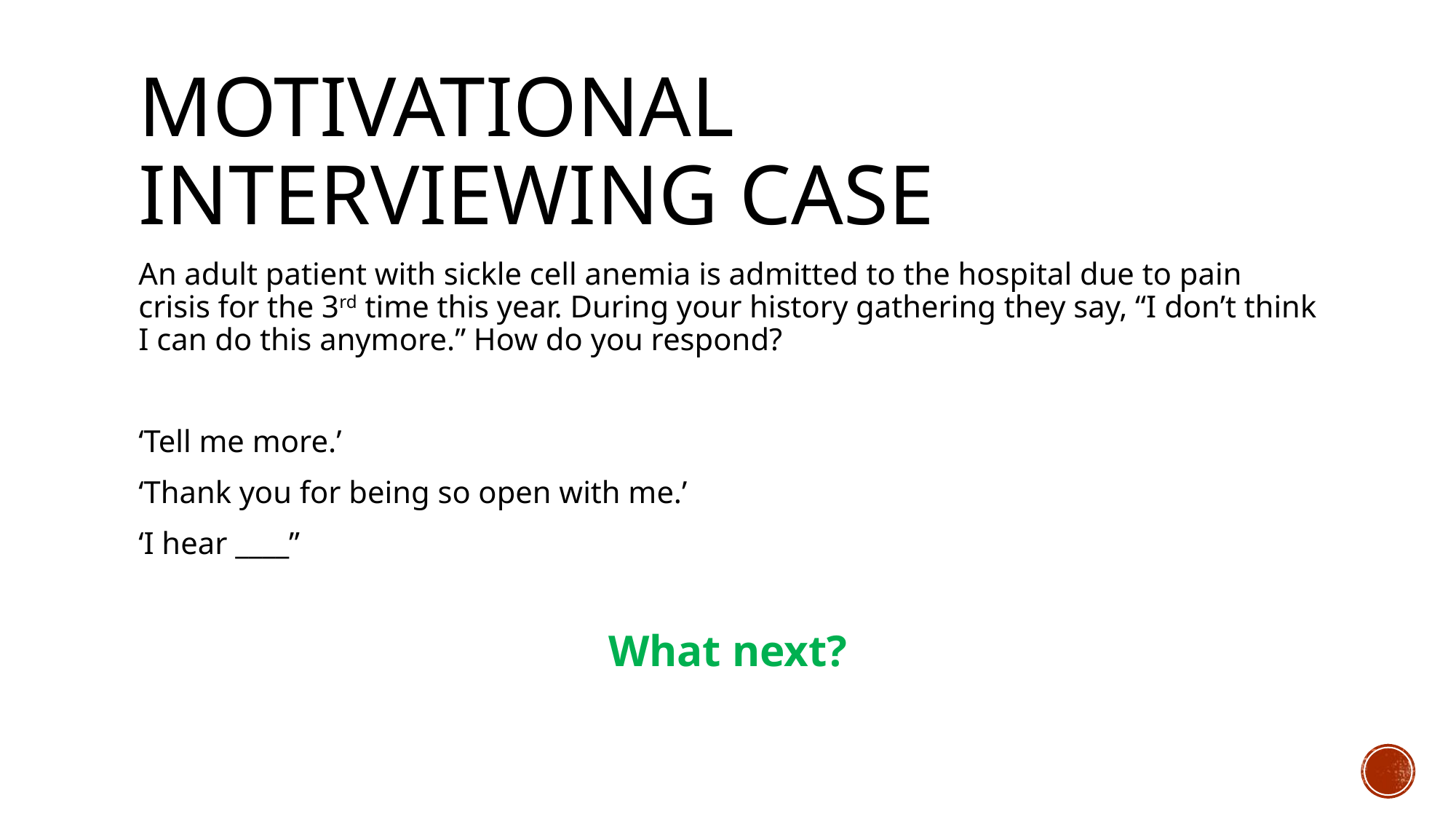

# Motivational Interviewing Case
An adult patient with sickle cell anemia is admitted to the hospital due to pain crisis for the 3rd time this year. During your history gathering they say, “I don’t think I can do this anymore.” How do you respond?
‘Tell me more.’
‘Thank you for being so open with me.’
‘I hear ____”
What next?

## Slide 39
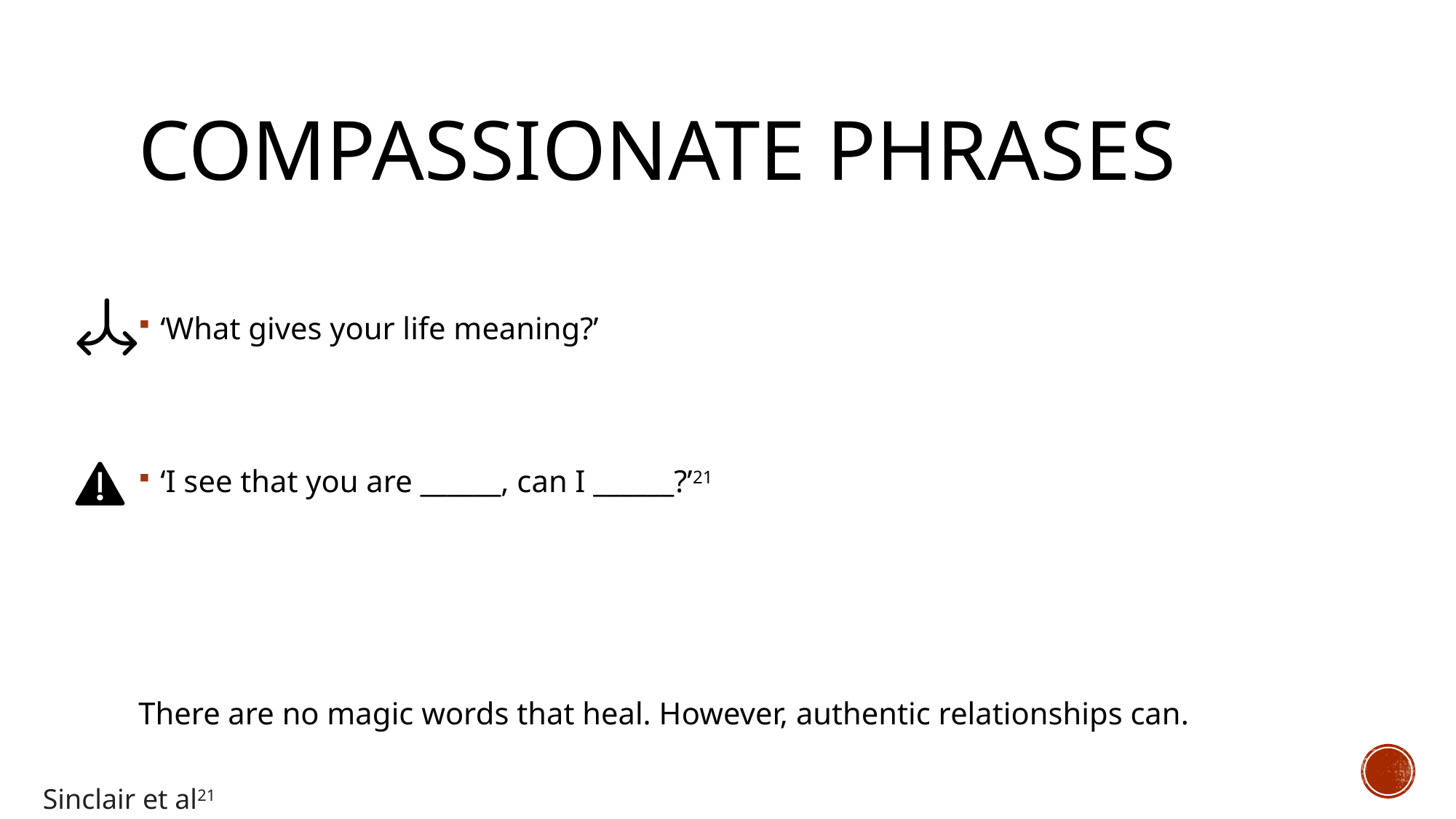

# Compassionate phrases
‘What gives your life meaning?’
‘I see that you are ______, can I ______?’21
There are no magic words that heal. However, authentic relationships can.
Sinclair et al21

## Slide 40
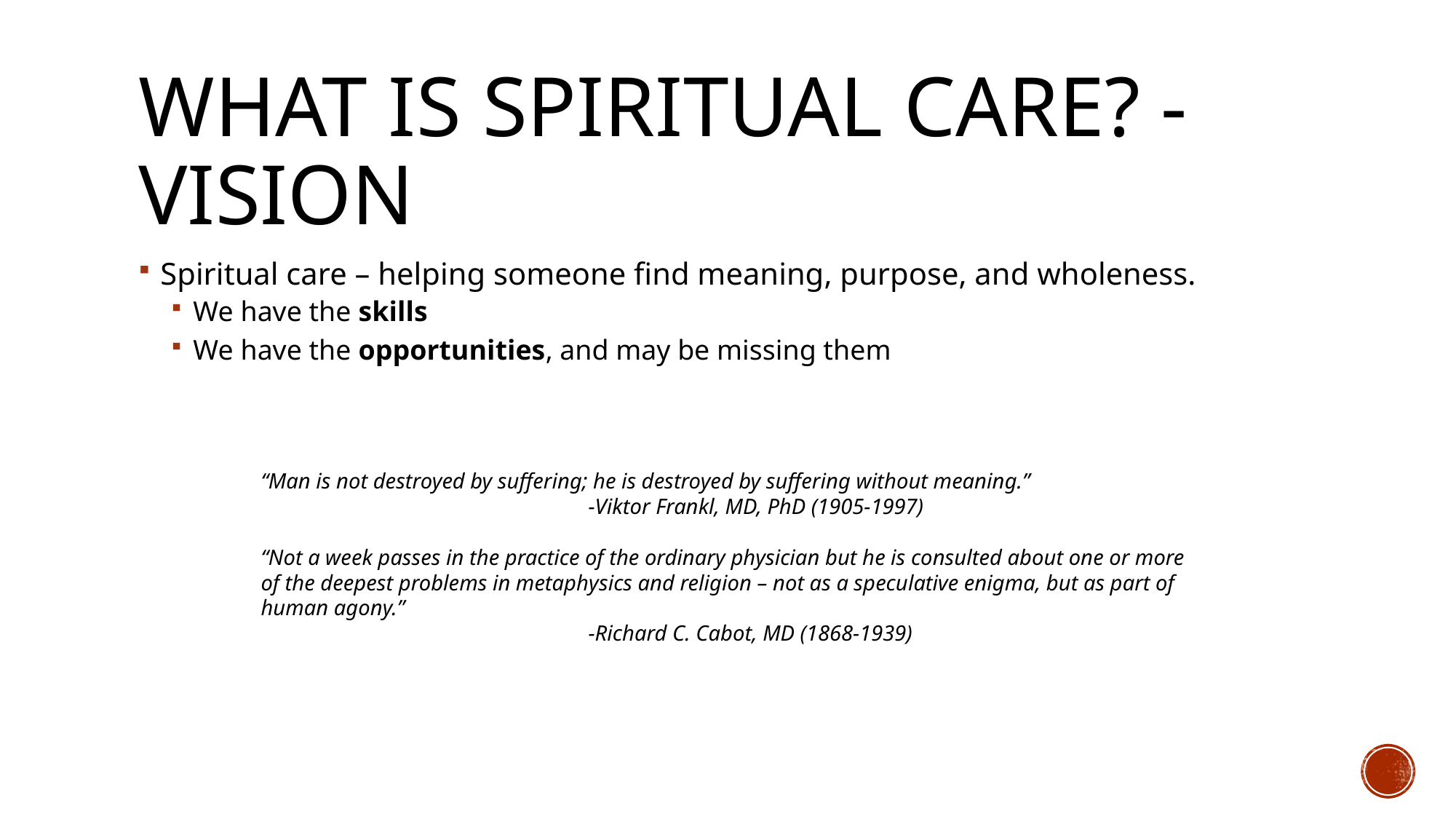

# What is Spiritual Care? - vISION
Spiritual care – helping someone find meaning, purpose, and wholeness.
We have the skills
We have the opportunities, and may be missing them
“Man is not destroyed by suffering; he is destroyed by suffering without meaning.”
			-Viktor Frankl, MD, PhD (1905-1997)
“Not a week passes in the practice of the ordinary physician but he is consulted about one or more of the deepest problems in metaphysics and religion – not as a speculative enigma, but as part of human agony.”
			-Richard C. Cabot, MD (1868-1939)

## Slide 41
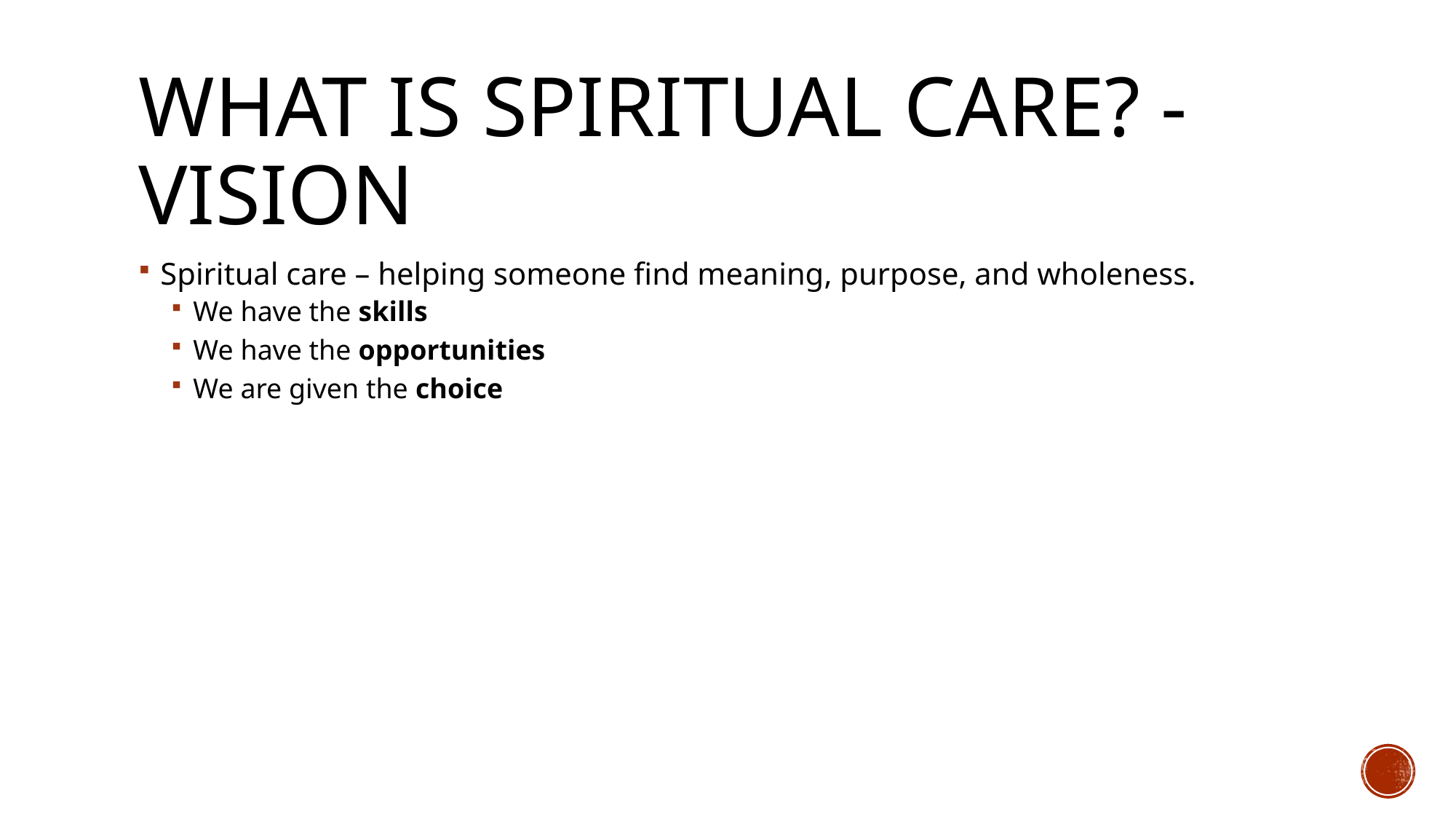

# What is Spiritual Care? - Vision
Spiritual care – helping someone find meaning, purpose, and wholeness.
We have the skills
We have the opportunities
We are given the choice

## Slide 42
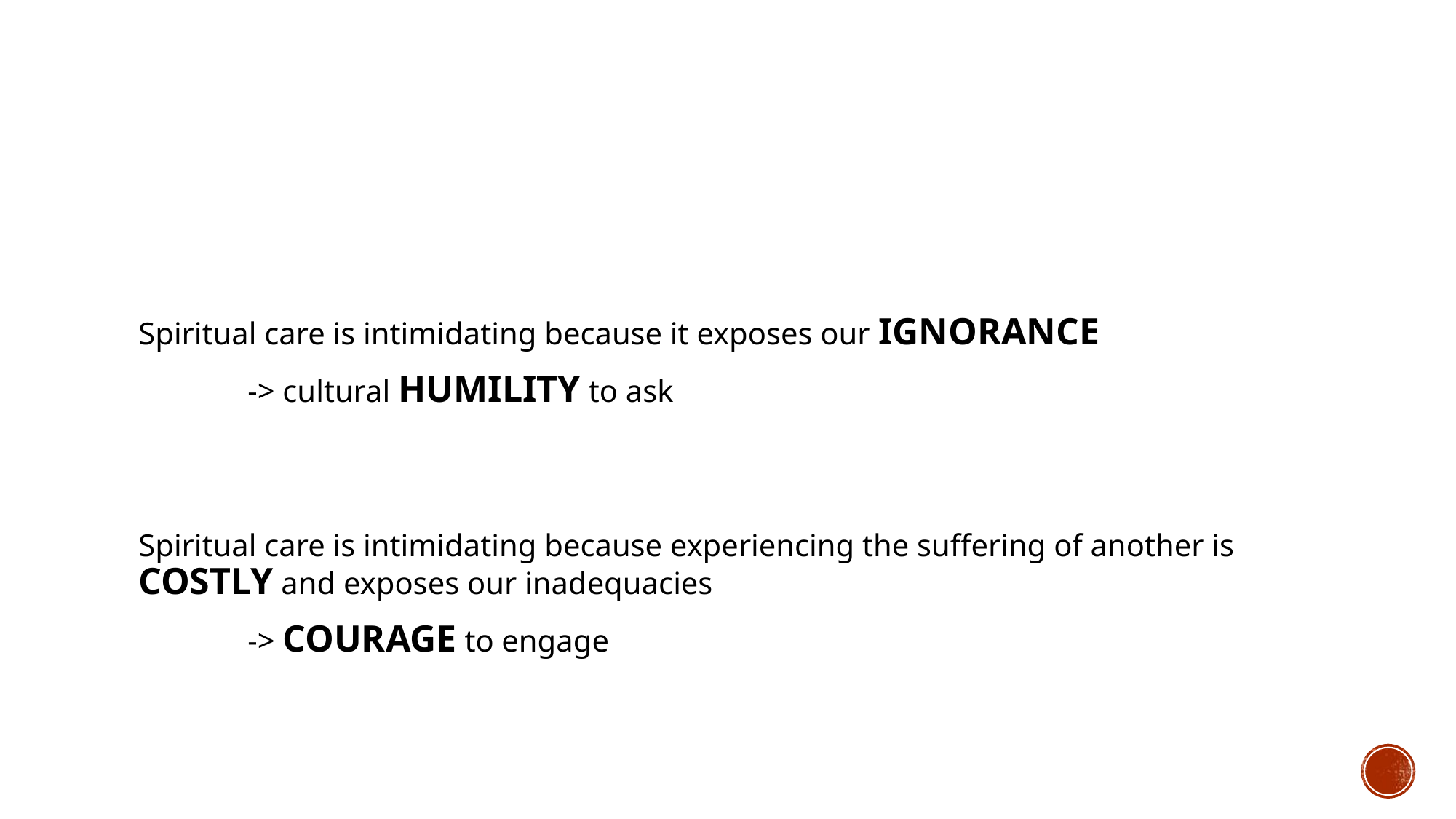

Spiritual care is intimidating because it exposes our IGNORANCE
	-> cultural HUMILITY to ask
Spiritual care is intimidating because experiencing the suffering of another is COSTLY and exposes our inadequacies
	-> COURAGE to engage

## Slide 43
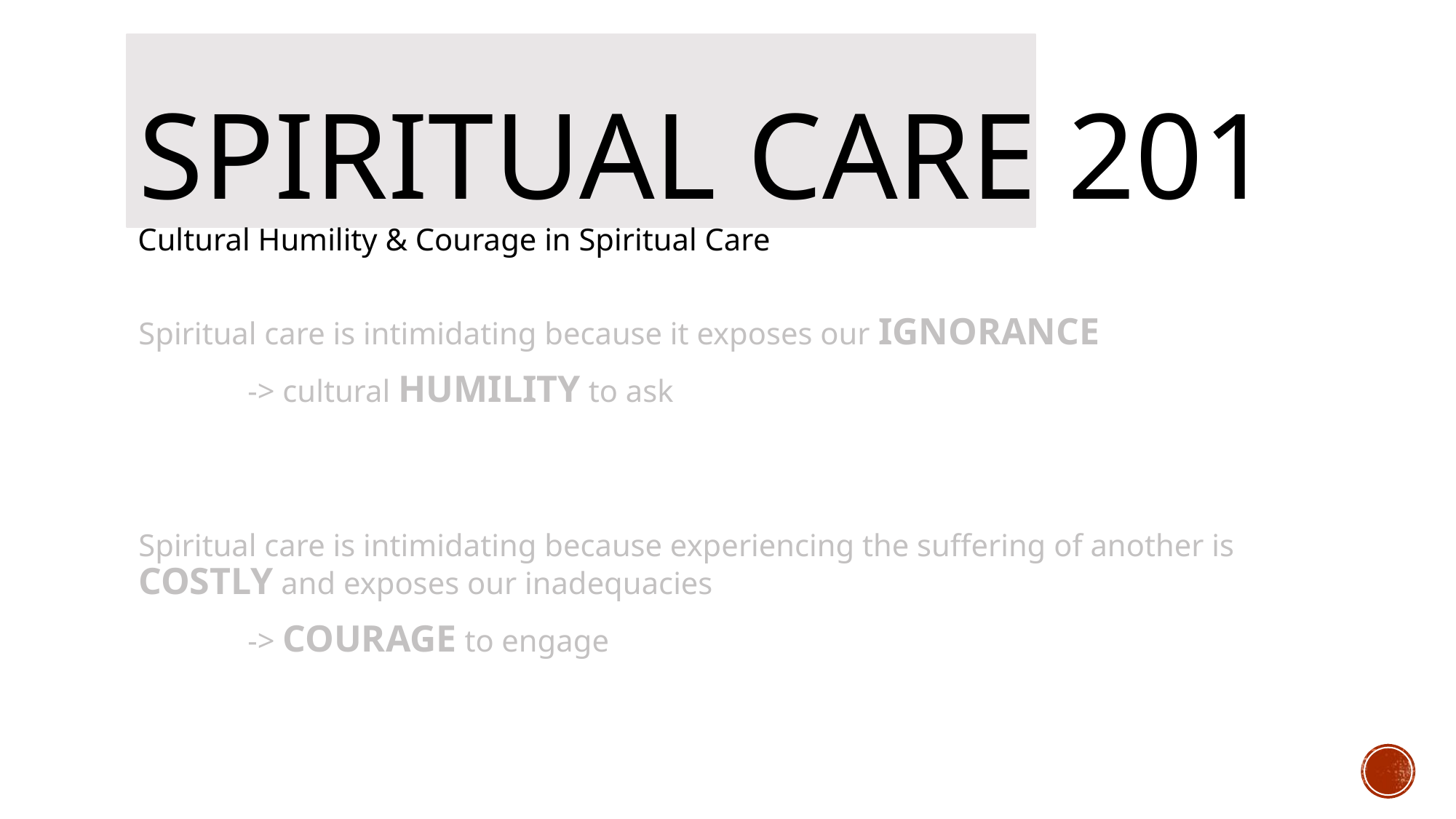

# Spiritual care 201
Cultural Humility & Courage in Spiritual Care
Spiritual care is intimidating because it exposes our IGNORANCE
	-> cultural HUMILITY to ask
Spiritual care is intimidating because experiencing the suffering of another is COSTLY and exposes our inadequacies
	-> COURAGE to engage

## Slide 44
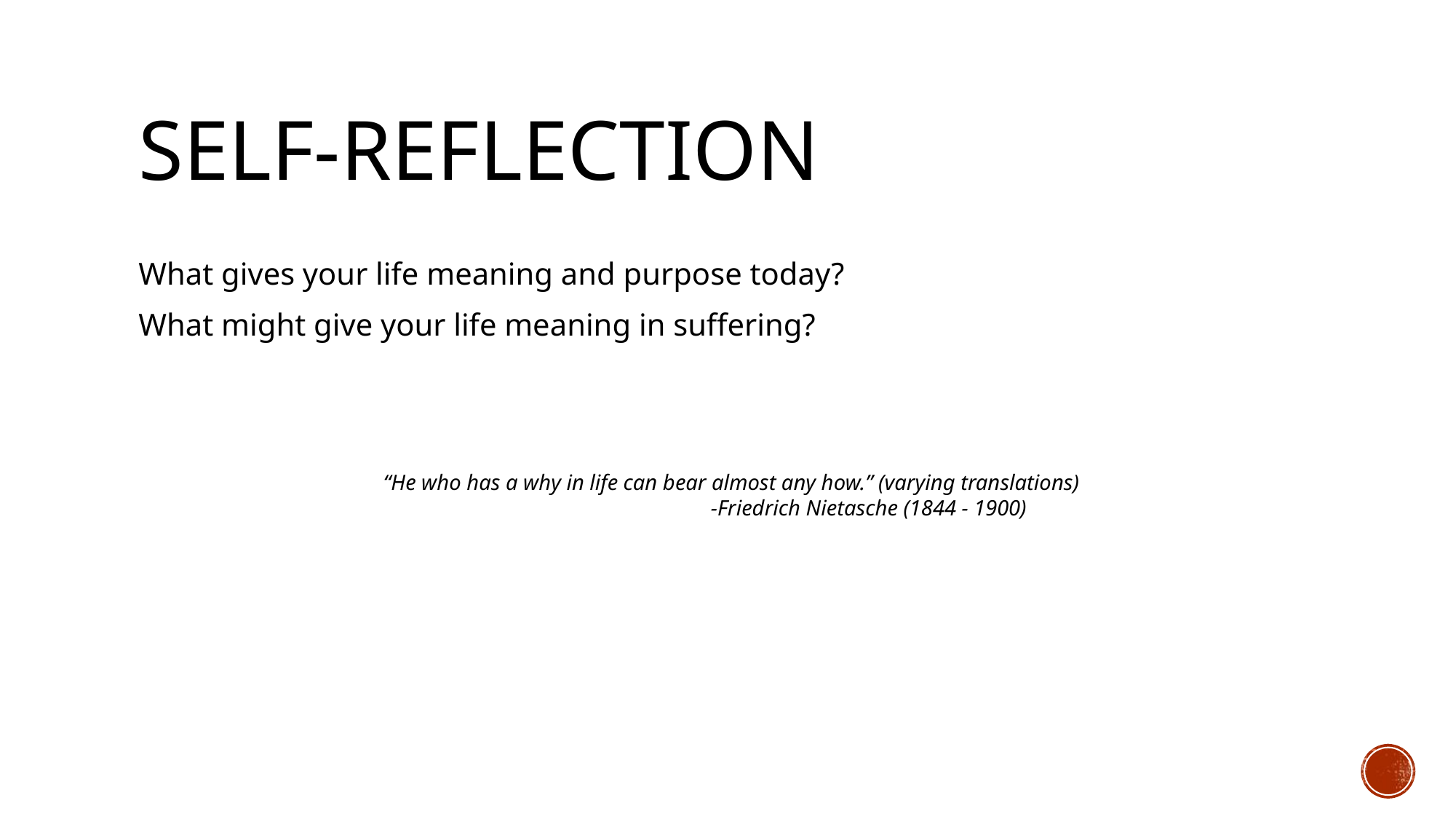

# Self-reflection
What gives your life meaning and purpose today?
What might give your life meaning in suffering?
“He who has a why in life can bear almost any how.” (varying translations)
			-Friedrich Nietasche (1844 - 1900)

## Slide 45
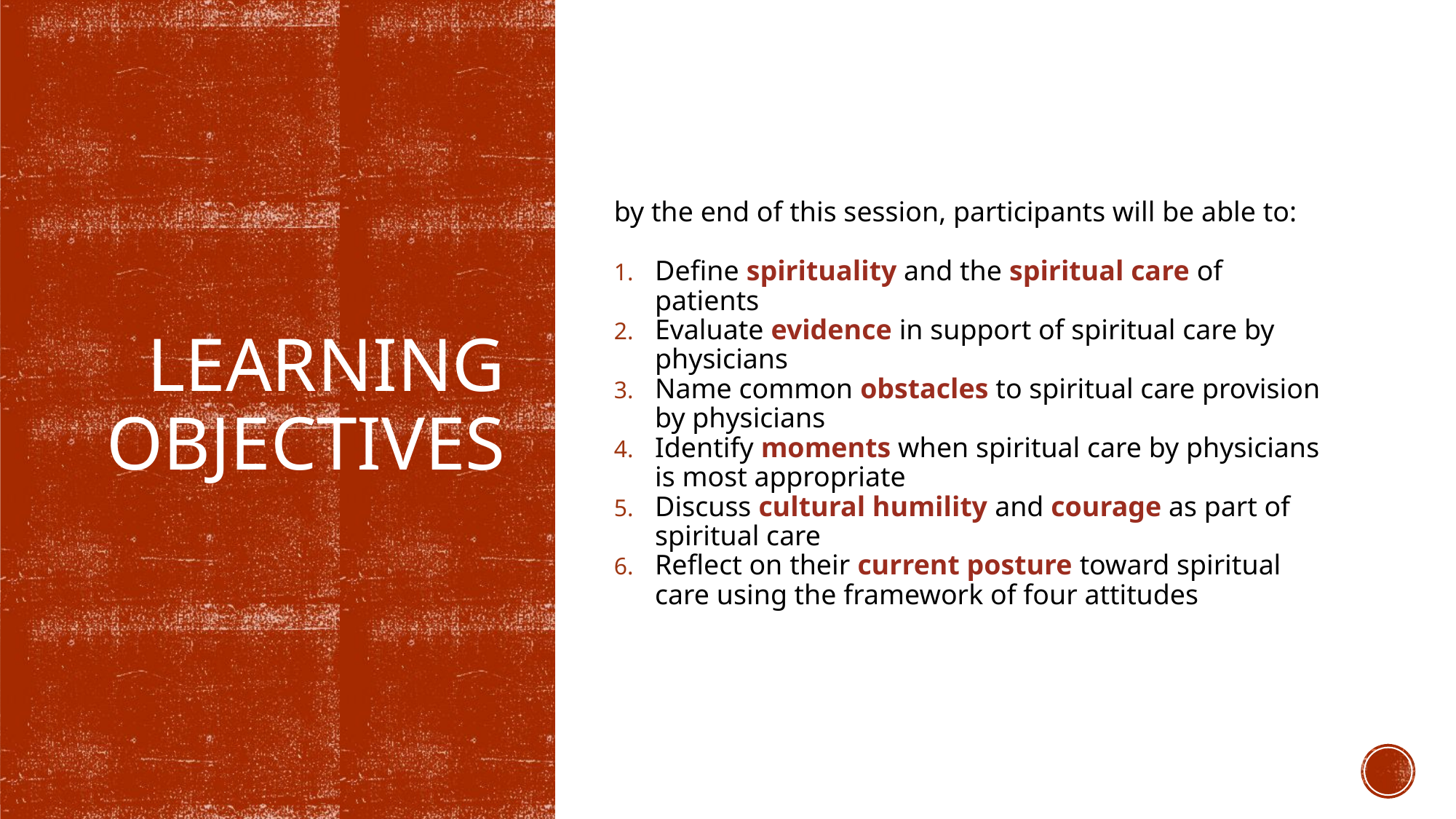

by the end of this session, participants will be able to:
Define spirituality and the spiritual care of patients
Evaluate evidence in support of spiritual care by physicians
Name common obstacles to spiritual care provision by physicians
Identify moments when spiritual care by physicians is most appropriate
Discuss cultural humility and courage as part of spiritual care
Reflect on their current posture toward spiritual care using the framework of four attitudes
# Learning Objectives

## Slide 46
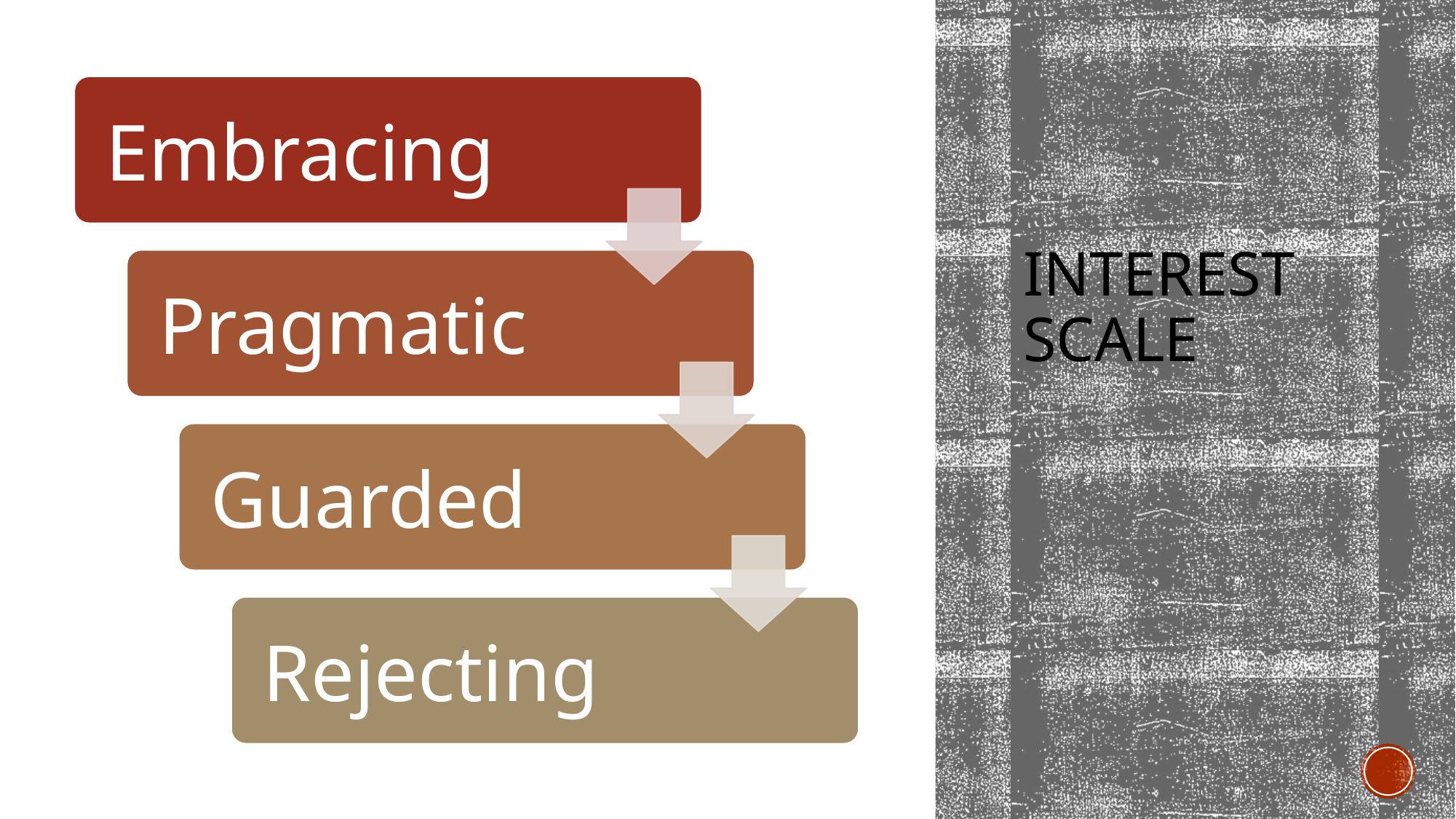

# Interest scale

## Slide 47
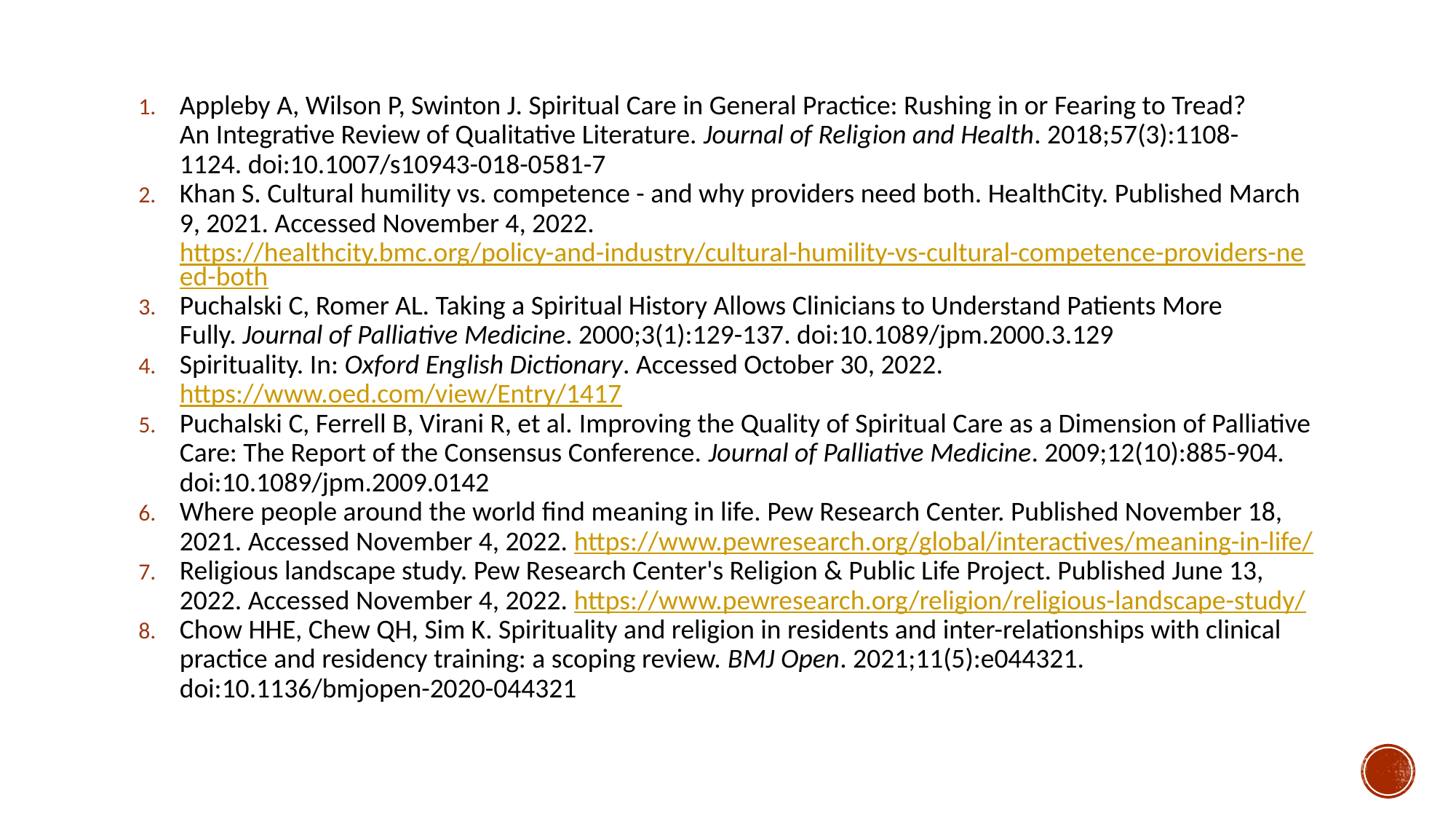

Appleby A, Wilson P, Swinton J. Spiritual Care in General Practice: Rushing in or Fearing to Tread? An Integrative Review of Qualitative Literature. Journal of Religion and Health. 2018;57(3):1108-1124. doi:10.1007/s10943-018-0581-7
Khan S. Cultural humility vs. competence - and why providers need both. HealthCity. Published March 9, 2021. Accessed November 4, 2022. https://healthcity.bmc.org/policy-and-industry/cultural-humility-vs-cultural-competence-providers-need-both
Puchalski C, Romer AL. Taking a Spiritual History Allows Clinicians to Understand Patients More Fully. Journal of Palliative Medicine. 2000;3(1):129-137. doi:10.1089/jpm.2000.3.129
Spirituality. In: Oxford English Dictionary. Accessed October 30, 2022. https://www.oed.com/view/Entry/1417
Puchalski C, Ferrell B, Virani R, et al. Improving the Quality of Spiritual Care as a Dimension of Palliative Care: The Report of the Consensus Conference. Journal of Palliative Medicine. 2009;12(10):885-904. doi:10.1089/jpm.2009.0142
Where people around the world find meaning in life. Pew Research Center. Published November 18, 2021. Accessed November 4, 2022. https://www.pewresearch.org/global/interactives/meaning-in-life/
Religious landscape study. Pew Research Center's Religion & Public Life Project. Published June 13, 2022. Accessed November 4, 2022. https://www.pewresearch.org/religion/religious-landscape-study/
Chow HHE, Chew QH, Sim K. Spirituality and religion in residents and inter-relationships with clinical practice and residency training: a scoping review. BMJ Open. 2021;11(5):e044321. doi:10.1136/bmjopen-2020-044321

## Slide 48
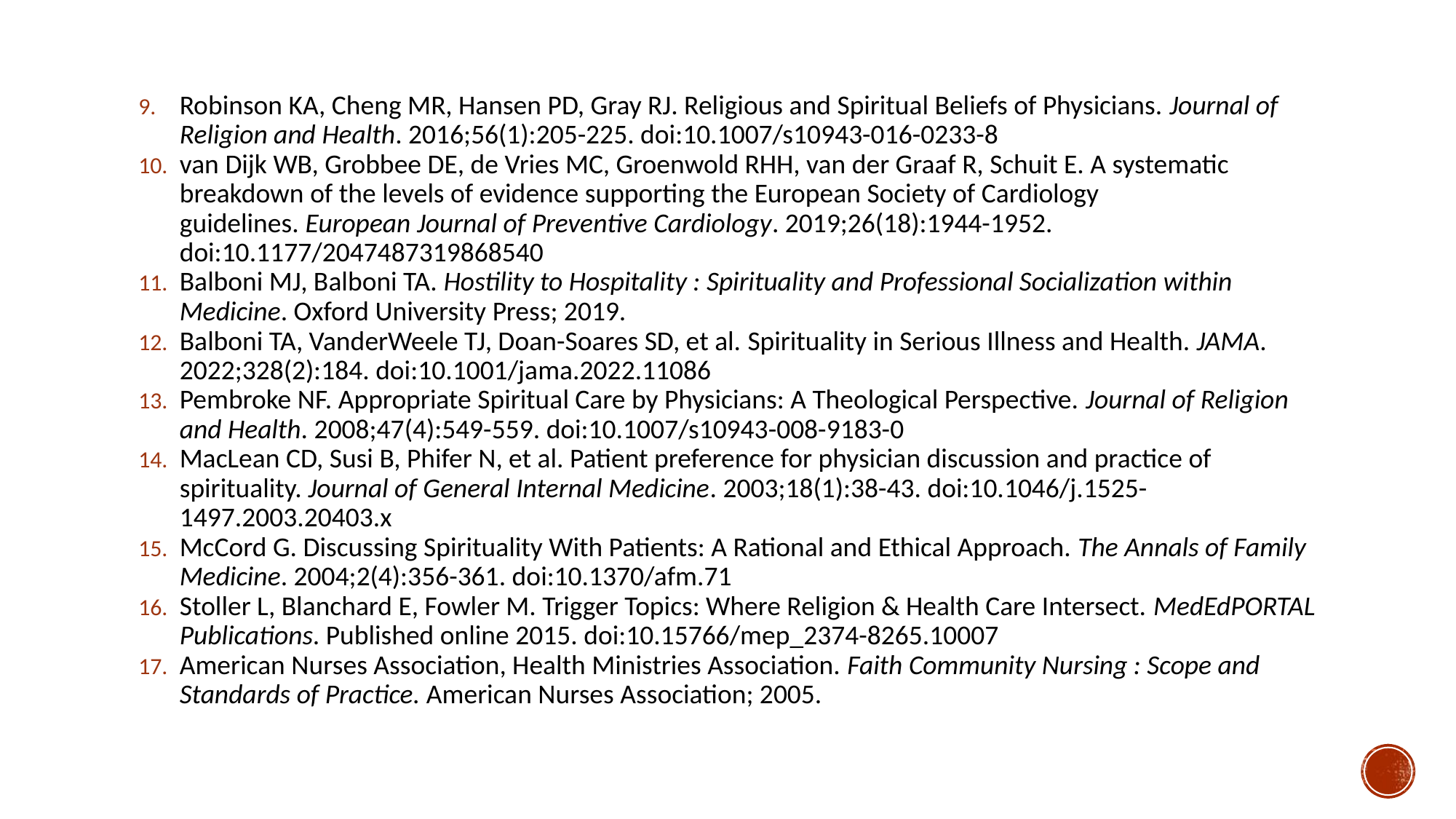

Robinson KA, Cheng MR, Hansen PD, Gray RJ. Religious and Spiritual Beliefs of Physicians. Journal of Religion and Health. 2016;56(1):205-225. doi:10.1007/s10943-016-0233-8
van Dijk WB, Grobbee DE, de Vries MC, Groenwold RHH, van der Graaf R, Schuit E. A systematic breakdown of the levels of evidence supporting the European Society of Cardiology guidelines. European Journal of Preventive Cardiology. 2019;26(18):1944-1952. doi:10.1177/2047487319868540
Balboni MJ, Balboni TA. Hostility to Hospitality : Spirituality and Professional Socialization within Medicine. Oxford University Press; 2019.
Balboni TA, VanderWeele TJ, Doan-Soares SD, et al. Spirituality in Serious Illness and Health. JAMA. 2022;328(2):184. doi:10.1001/jama.2022.11086
Pembroke NF. Appropriate Spiritual Care by Physicians: A Theological Perspective. Journal of Religion and Health. 2008;47(4):549-559. doi:10.1007/s10943-008-9183-0
MacLean CD, Susi B, Phifer N, et al. Patient preference for physician discussion and practice of spirituality. Journal of General Internal Medicine. 2003;18(1):38-43. doi:10.1046/j.1525-1497.2003.20403.x
McCord G. Discussing Spirituality With Patients: A Rational and Ethical Approach. The Annals of Family Medicine. 2004;2(4):356-361. doi:10.1370/afm.71
Stoller L, Blanchard E, Fowler M. Trigger Topics: Where Religion & Health Care Intersect. MedEdPORTAL Publications. Published online 2015. doi:10.15766/mep_2374-8265.10007
American Nurses Association, Health Ministries Association. Faith Community Nursing : Scope and Standards of Practice. American Nurses Association; 2005.

## Slide 49
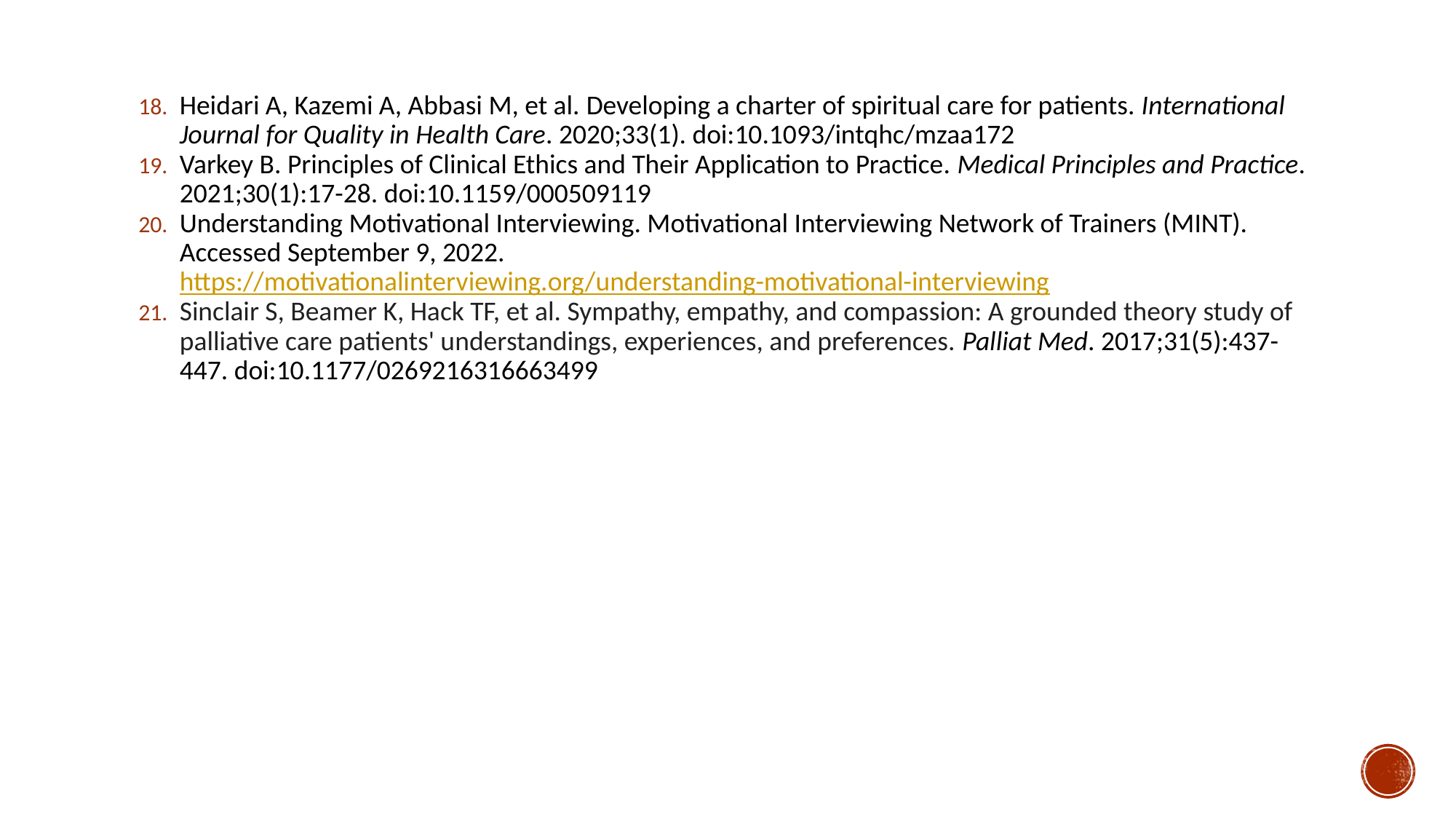

Heidari A, Kazemi A, Abbasi M, et al. Developing a charter of spiritual care for patients. International Journal for Quality in Health Care. 2020;33(1). doi:10.1093/intqhc/mzaa172
Varkey B. Principles of Clinical Ethics and Their Application to Practice. Medical Principles and Practice. 2021;30(1):17-28. doi:10.1159/000509119
Understanding Motivational Interviewing. Motivational Interviewing Network of Trainers (MINT). Accessed September 9, 2022. https://motivationalinterviewing.org/understanding-motivational-interviewing
Sinclair S, Beamer K, Hack TF, et al. Sympathy, empathy, and compassion: A grounded theory study of palliative care patients' understandings, experiences, and preferences. Palliat Med. 2017;31(5):437-447. doi:10.1177/0269216316663499

## Slide 50
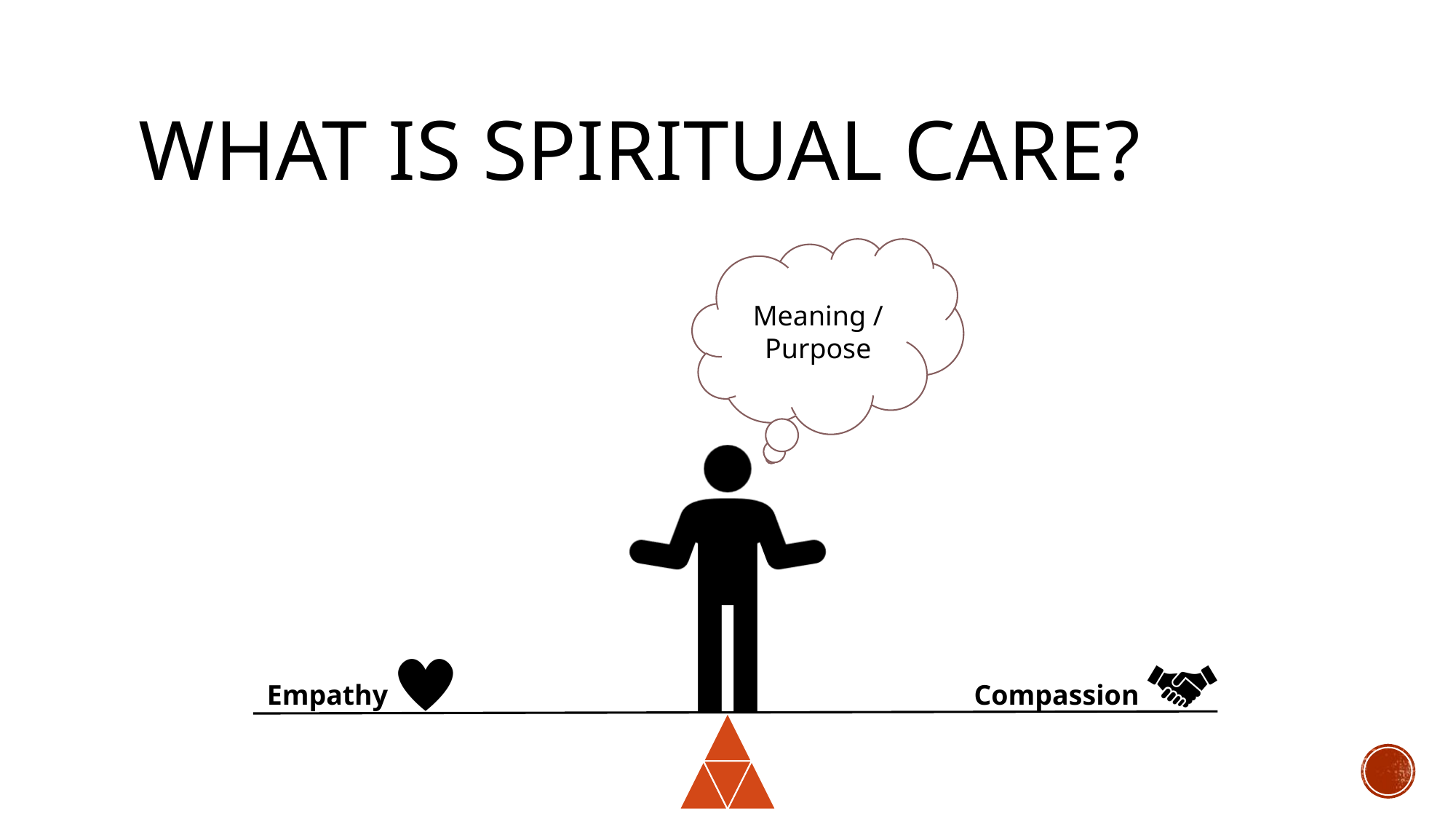

# What is Spiritual Care?
Meaning / Purpose
Empathy
Compassion
