# Supplementary material for: Enriching Spiritual Care in Medical Residents Through Cultural Humility and Courage
Source: MedEdPORTAL. 2024 Jul 26;20:11423. doi: 10.15766/mep_2374-8265.11423 (PMC11272909; doi:10.15766/mep_2374-8265.11423)
Supplement: Supplementary file 1 — Cultural Humility and Courage in Spiritual Care.pptxFacilitator Guide for Spiritual Care Session.docxSpiritual Care Reflection Questions.docxSpiritual Care Surveys.docx [file mep_2374-8265.11423-s001.zip › B. Facilitator Guide for Spiritual Care Session.docx]

**Appendix B – Facilitator Guide for the Spiritual Care Session**

We developed this 90-minute session to speak directly to resident physicians about the relationships between medicine and spirituality and the nature of spiritual care.  Given this audience and purpose, we recommend the session be facilitated by a physician. The session requires a computer with projector and PowerPoint setup, but also benefits from an internet connection for slides 13-14. We advise facilitators to familiarize themselves with the presentation and this facilitator guide in advance of the session as the slides are not intended to be comprehensive.

This Facilitator Guide provides an in-depth exposition of each presentation slide in the Spiritual Care Session. It also includes the approximate time recommended for each individual slide and the running total time aligned on the right. It may be used as a true script, though the presentation would likely be more effective in one’s own words. For this reason, we include suggested highlights to point out phrases for the facilitator to present with more flexibility without missing any key concept.

**Highlight Color Key:**

Green – opportunity for reflection and discussion

Grey – key word or phrase

**Educational Objectives**

By the end of this activity, learners will be able to:

1. Define spirituality and the spiritual care of patients

2. Evaluate evidence in support of spiritual care by physicians

3. Name common obstacles to spiritual care provision by physicians

4. Identify moments when spiritual care by physicians is most appropriate

5. Discuss cultural humility and courage as part of spiritual care

6. Reflect on their current posture toward spiritual care using the framework of four attitudes

**Slide 1 – Title slide**

**Slide 2-3 – Disclosures and learning objectives**   **1min (slide);** 1min (total)

*Consider disclosing personal background or biases regarding spiritual care*

**Slide 4 – Interest scale**  **1min (slide);** 2min (total)

At least 4 different types of attitudes to providing spiritual care exist in the literature ^1^: embracing, pragmatic, guarded, and rejecting. These attitudes may be fluid.

*Reflection: Which attitude fits your current posture toward spiritual care?*

- *Embracing – I enthusiastically approve of spiritual care and accept it as part of my role.*
- *Pragmatic – I approve of and would offer spiritual care if it is deemed to help or be wished by the patient.*
- *Guarded – I have reservations about spiritual care but might consider offering it in some limited contexts.*
- *Rejecting – I am opposed to spiritual care and do not think it is part of my role.*

**Slide 5 – Cultural Humility vs Competency ^2^**   **2min (slide);** 4min (total)

Cultural competency is the ability to engage knowledgably with people across cultures. Though admirable, there is a danger of stereotyping and bias as we search for categorical knowledge about groups of people (e.g., Muslim people do ____). Cultural humility is a necessary partner of competency: adopting a posture of curiosity toward the individual and a willingness to expose our own ignorance for correction. It acknowledges that even in sameness there is difference. I invite you to embrace the discomfort of ignorance with humility.

**Slide 6 – Background: What you may have learned in medical school #1**   **2min (slide);** 6min (total)

As medicine has further affirmed the importance of spiritual care, governing bodies are requiring its provision and medical schools are incorporating it into their curriculum. In medical school, the emphasis of spiritual care training is within obtaining a social history. This usually entails a list of screening questions with further history gathered if the patient answers in the affirmative (eg. sex, drugs, spirituality).

**Slide 7 – Background: What you may have learned in medical school #2 7min (slide);** 13min (total)

The most common tool for obtaining a basic spiritual history is the FICA which asks about the presence of faith/belief, its importance to the patient, whether they are part of a spiritual community, and how the patient’s belief will impact their care.^3^ This tool is easily accessible on the internet if you do not have it committed to memory. If a spiritual need is identified, physicians are often trained to refer the patient to a chaplain: spiritual care specialists.

Yet, despite this training most physicians do not regularly incorporate it into their practice or know what to do with the information. Also, the terms spirituality and spiritual care remain nebulous. One purpose of this session is to provide clarity while creating a space for reflection.

*Discussion: in a small group, discuss your definitions of spirituality (5min)*

**Slide 8-9 – Background: Defining Spirituality**   **3min (slide);** 16min (total)

*Spirituality (common) ^4^: the quality of being concerned with the human spirit or soul as opposed to material or physical things.*

In the popular worldview of trialism, there is a body, a mind, and then something transcendent called the spirit. However, academia has a slightly expanded definition.

*Spirituality (academic) ^5^: the way individuals seek and express* ***meaning and purpose*** *and the way they experience their connectedness to the moment, to self, to nature, and to the significant and sacred.*

Latin ‘spiritus’ = “breath”, and spirituality can be seen as the search for what gives life ‘breath’. Academic definition emphasizes an individual nature of beliefs and practices, especially pertaining to meaning and purpose.

*Religion (academic) ^5^: An organized system of beliefs, rituals, and practices with which an individual identifies and associates and includes a relationship with a divine being.*

Latin ‘religare’ = “to tie”. The definition emphasizes a common/corporate nature of beliefs and practices.

Main points 1) Not all spirituality is rooted in religion.

2) Even people who are not religious or do not believe in higher/inanimate beings are still searching for the meaning and purpose of their lives. In this way, ‘spirituality’ is universal and weighty.

**Slide 10-12 – Spirituality pictures #1, non-religious**   **1min (slide);** 17min (total)

**What does spirituality look like for different people?**

Recreation – many people find meaning and purpose in adventure, nature, music, etc…

Vocation – many people find meaning and purpose in their career. E.g., care of the sick or advancing their field

Family – when asked about the meaning of life, the most common response involved people’s family.^6^

**Slide 13-14 – Background – Religious Prevalence**   **4min (slide);** 21min (total)

*Reflection: What is your percentage guess regarding religious prevalence in your region?*

- Explore Pew Research Center’s Religious Landscape Study database for your area.^7^

https://www.pewresearch.org/religion/religious-landscape-study/

*After opening the webpage facilitators will find a table of religious prevalence in the United States. Facilitators can scroll down to the map and click on the state where they are presenting in order to pull up more relevant data.*

- - Particular attention to: belief in God, importance of religion, and sources of guidance
  - Most patients have belief in God and assign importance to religion.

Most physicians, including trainees, also believe in God.^8,9^ Even though spirituality is broader than just religion or relating to a ’spirit’, most people in most regions believe in God and identify as religious.

**Slide 15-18 – Spirituality pictures #2, religious**   **3min (slide);** 24min (total)

Talambralu – a ritual of happiness during weddings in certain regions of India. Bride and groom pour rice on each other’s heads while a Hindu family priest (purohit) chants mantras.

Shabbat – a Jewish weekly day of rest. Often involves festive meals with wine, singing, and prayer.

Prayer – in Islam, Muslims are encouraged to pray at 5 specific times per day at least. Prayer is an important daily activity for many people of diverse beliefs and backgrounds.

Baptism – a ritual of purification involving water found in various religions, commonly associated with Christianity.

Last rites – a primarily Catholic tradition administered to a person in danger of dying involving confession, prayer, and other rituals to prepare them for death.

Cultural humility & Christianity – although a majority of people in the United States identify as Christian, there are dozens of different denominations with varying practices and numerous non-denominational churches as well. Even within a community, personal practices and the importance of faith can vary. The best way to understand a patient’s spirituality is to ask about it in humility even if we think we may know already.

**Slide 19-22 – Background – Secular Medicine**   **13min (slide);** 37min (total)

Modern medicine understands itself as a secular (non-spiritual) practice built upon scientific evidence. Barring the power of black clouds and full moons, as a field we equate secularity with science and spirituality with superstition. This devalues the lived experience of spirituality, which many patients identify as critical. Further, it fosters a moral opposition to concepts that transcend the tangible. We know that if an idea is not testable, repeatable, observable, and falsifiable, it is not scientific. But, how do we account for the truths and ideas we struggle to measure with science? How much of medical knowledge meets scientific criteria?

*Discussion: Discuss brief case in small groups. There is no one correct answer.*

A 2019 study of European College of Cardiology guidelines shows that of Class I (strong) recommendations, only 21% were based on Level A (high quality) evidence while 50% were based on Level C (low quality) evidence, meaning only expert opinion as the studies have serious flaws.^10^ We accept these things due to common sense. We also don’t have the resources to test every possible theory.

Though some can argue that medicine is secular we cannot claim it is completely scientific. There are many truths we hold and ideas we act upon even though we cannot test and repeat them using empirical measures. We use our common sense or educated judgements to move forward through the uncertainty in good faith. How much more will the limits of empiricism be exposed as science struggles to make claims about something of the human spirit? Spiritual care will require both our common sense and our good faith in order to properly care for those who are suffering.

*Discussion: in the large group discuss reactions, comments, and disagreements (5min)*

**Slide 23 – Topics to Avoid?**   **6min (slide);** 43min (total)

Doctors are privileged to be invited into some of the most intimate parts of a patient’s life. There are many topics that may be considered taboo to the general public that are integral to a patient’s health and well-being. Medical professionals already commonly ask patients about many of these taboo topics.

*Discussion: In a small group, discuss why doctors do not often ask patients about spirituality. (5min)*

**Slide 24 – What hinders spiritual care?^11^**   **2min (slide);** 45min (total)

It is common for doctors to shy away from asking about spirituality. However, evidence shows that this is not because doctors do not value spiritual care or desire to provide it. It is also not because of proven negative impact on physician-patient relationships, though this is a common fear.

Instead, studies show that inadequate training, discordant beliefs, physician job description, and concern over physician-patient power dynamics are the greatest reasons physicians do not offer spiritual care. Interestingly, although time and privacy were listed as among the most common physician concerns, they were not associated with actual frequency of providing spiritual care.

These are real barriers in which to reflect for each individual doctor. But as you weigh some of the barriers, let us consider some of the advantages. Does spirituality influence health outcomes? Does the patient want to talk about spirituality? These questions are where spirituality may differ from politics.

**Slide 25 – Does spirituality influence health outcomes?**   **3min (slide);** 48min (total)

“Despite increasing data linking spirituality with improved health outcomes… such issues remain largely outside standard considerations regarding health.”^12^ This statement may be overly strong (‘black and white’) relative to the quality of evidence we have. A more appropriate, though lengthy, summary could be:

“Qualitative and quantitative research studies have demonstrated that there are likely to be associations between certain religious and spiritual variables and health outcomes. These relationships may not be simple, and many of the studies are methodologically imperfect, but as more sophisticated research proceeds, these associations have persisted though there is some discussion about causality.“^1^

The nature of spirituality makes it hard to measure. Although there are positive associations, Pembroke argues that focusing on these and treating spirituality as a means to an end degrades its essence.^13^ We can ask, is spirituality only valuable if it has a measurable effect, regardless of the importance to the patient?

**Slide 26-27 – Do patients want you to ask? 3min (slide);** 51min (total)

There are studies with data supporting that the majority of patients want to talk about spirituality. Other studies report more modest results. The difference is likely in the context. The sicker the patient, the more important spirituality becomes to them.^14^ Another study showed a similar trend, but higher percentages.^15^ It also reported high percentage in the setting of addiction, chronic pain, other long-term serious illness, and during the medical history on an initial doctor’s visit.

**Slide 28-30 – Timing – Suggestions, ‘Trigger Topics’ for conflict**   **2min (slide);** 53min (total)

The earlier data give us an idea that patients may desire most to be asked about their spirituality with new evaluations, major health change, or psychosocial distress. But, another important time for discussion of spirituality is when medicine and spirituality seem to quarrel. These ‘trigger topics’ can be frustrating for physicians because they often involve patients declining a recommended medical intervention.^16^ These situations are fertile ground to reflect, practice our cultural humility, and provide spiritual care. But what actually is spiritual care?

**Slide 31-32 – What is Spiritual Care? – General**  **5min (slide);** 58min (total)

*Spiritual care ^17^: interventions, individual or communal, that facilitate the ability to express the integration of the body, mind, and spirit to achieve wholeness, health, and a sense of connection to self, others, and/or a higher power.*

When we remember our definition of spirituality, we can summarize spiritual care as helping someone find meaning, purpose, and wholeness. Spiritual care is more than just gathering a history. It is especially important when there is not a possibility of physical cure or if the illness is life-altering. How can physicians do this? Heidari et al developed a comprehensive charter listing the components of spiritual care based on patient and physician surveys.^18^ Many components and techniques of spiritual care can be summarized in basic clinical ethics and motivational interviewing. These skills are already being honed by the schooling and life experience inherent in becoming a physician.

**Slide 33-34 – What is Spiritual Care? – Ethics**   **4min (slide);** 62min (total)

*Principles of clinical ethics – beneficence, nonmaleficence, autonomy, justice.^19^*

Ethics case: this case places the physician in a dilemma between the benefit of reducing morbidity with blood transfusion and respecting the patient’s autonomy to refuse the intervention. Most ethicists (and lawyers) would arrive at the conclusion to accept a competent adult’s refusal of blood products. The purposes of the case are to 1) educate physicians that many people who identify as Jehovah’s witness decline blood transfusion due to risk of social ostracization and/or possibly jeopardizing their eternal salvation, 2) show physicians that they are already engaging in spiritual care with ethics and have the tools to continue.

**Slide 35-39 – What is Spiritual Care? – Motivational interviewing 6min (slide);** 68min (total)

*Motivational Interviewing – spirit of partnership, evocation, acceptance, and compassion. Techniques of open questions, affirmation, reflection, summarizing, attending to language of change, exchange of information.^20^*

Doctors most often think of motivational interviewing (MI) when attempting to convince a patient to make different choices surrounding a particular behavior. This, however, is contrary to the very spirit of MI to resist persuasion. Instead, the intent of MI is to motivate commitment and process ambivalence toward a patient’s own goals based on what they value. In a way, MI is healthcare’s attempt to illustrate and teach empathy (understanding the suffering of another) and compassion (transforming the suffering of another). These two virtues need not be limited to just the context of behavior change. When we offer empathy and compassion on a fulcrum of clinical ethics, we build a stable foundation to support the patient as they search for the breath of life, meaning, and purpose in their current situation that only they can find. This is spiritual care.

Motivational interviewing case: this case places the physician with a patient in psychosocial distress related to an incurable illness complicated by chronic pain (opportune time for spiritual care). There are countless appropriate initial responses, many of which will be open-ended, affirmations, or reflections as we practice our empathy. After the initial response, what could come next?

We have the opportunity to be a part of transforming a patient’s suffering through compassion. Instead of discussing a specific behavior, we can wade into the murky waters of ambivalence about meaning and purpose. A simple place to begin is “what gives your life meaning?”. After this opening, the conversation could involve increasingly complex reflections or naming an unstated undertone of a patient’s responses. These are motivational interviewing skills. We could also wield our power to see a need and fill it. A small study surveying advanced cancer patients found that small acts of kindness were key and powerful components of their experience of compassion.^21^ Physicians, however, should be cautious of using these small acts as a substitute for discussing the patient’s suffering. Despite all of these possibilities there are no magic words or techniques that heal. The healing (or change) comes from within oneself in the context of supportive, safe, and authentic relationships. Both suffering and ambivalence are scary, and we often only dare to explore them when someone comes with us.

**Slide 40-43 – What is Spiritual Care? – Vision**   **4min (slide);** 72min (total)

We have the skills to offer spiritual care. We also have the opportunities and may be missing them. Patients suffer before us daily searching for meaning. Therefore, we have the choice of whether to engage more deeply.

Spiritual care is intimidating because it exposes our ignorance. We are called to cultural humility: offering our ignorance unashamed on behalf of the patient we care to know. Spiritual care is intimidating because experiencing the suffering of another is costly and exposes our inadequacies. We are called to courage to engage imperfectly in a healing relationship. We will fail. We will at times respond poorly or not know what to say. We are unable to define a patient’s meaning and purpose for them. This is Spiritual Care 201. But, if we hold tightly to humility and courage, empathy and compassion will come, and our patients will heal in ways that no medication can offer.

**Slide 44 – Self-Reflection**  **10min (slide);** 82min (total)

*Facilitators may choose to defer this slide until after taking final questions if it serves the audience logistically.*

Take time to reflect on the following questions. It is much easier to guide someone on a path you have already walked yourself.

*Reflection: What gives your life meaning and purpose today? What might give your life meaning in suffering?*

**Slide 45 – Learning objectives review**   **1min (slide);** 83min (total)

**Slide 46 – Interest scale**   **1min (slide);** 84min (total)

**Slide 47-50 – References & Diagram Slide for Questions**

**References:**

1. Appleby A, Wilson P, Swinton J. Spiritual Care in General Practice: Rushing in or Fearing to Tread? An Integrative Review of Qualitative Literature. *Journal of Religion and Health*. 2018;57(3):1108-1124. doi:10.1007/s10943-018-0581-7
2. Khan S. Cultural humility vs. competence - and why providers need both. HealthCity. Published March 9, 2021. Accessed November 4, 2022. <https://healthcity.bmc.org/policy-and-industry/cultural-humility-vs-cultural-competence-providers-need-both>
3. Puchalski C, Romer AL. Taking a Spiritual History Allows Clinicians to Understand Patients More Fully. *Journal of Palliative Medicine*. 2000;3(1):129-137. doi:10.1089/jpm.2000.3.129
4. Spirituality. In: *Oxford English Dictionary*. Accessed October 30, 2022. <https://www.oed.com/view/Entry/1417>
5. Puchalski C, Ferrell B, Virani R, et al. Improving the Quality of Spiritual Care as a Dimension of Palliative Care: The Report of the Consensus Conference. *Journal of Palliative Medicine*. 2009;12(10):885-904. doi:10.1089/jpm.2009.0142
6. Where people around the world find meaning in life. Pew Research Center. Published November 18, 2021. Accessed November 4, 2022. <https://www.pewresearch.org/global/interactives/meaning-in-life/>
7. Religious landscape study. Pew Research Center's Religion & Public Life Project. Published June 13, 2022. Accessed November 4, 2022. <https://www.pewresearch.org/religion/religious-landscape-study/>
8. Chow HHE, Chew QH, Sim K. Spirituality and religion in residents and inter-relationships with clinical practice and residency training: a scoping review. *BMJ Open*. 2021;11(5):e044321. doi:10.1136/bmjopen-2020-044321
9. Robinson KA, Cheng MR, Hansen PD, Gray RJ. Religious and Spiritual Beliefs of Physicians. *Journal of Religion and Health*. 2016;56(1):205-225. doi:10.1007/s10943-016-0233-8
10. van Dijk WB, Grobbee DE, de Vries MC, Groenwold RHH, van der Graaf R, Schuit E. A systematic breakdown of the levels of evidence supporting the European Society of Cardiology guidelines. *European Journal of Preventive Cardiology*. 2019;26(18):1944-1952. doi:10.1177/2047487319868540
11. Balboni MJ, Balboni TA. *Hostility to Hospitality : Spirituality and Professional Socialization within Medicine*. Oxford University Press; 2019.
12. Balboni TA, VanderWeele TJ, Doan-Soares SD, et al. Spirituality in Serious Illness and Health. *JAMA*. 2022;328(2):184. doi:10.1001/jama.2022.11086
13. Pembroke NF. Appropriate Spiritual Care by Physicians: A Theological Perspective. *Journal of Religion and Health*. 2008;47(4):549-559. doi:10.1007/s10943-008-9183-0
14. MacLean CD, Susi B, Phifer N, et al. Patient preference for physician discussion and practice of spirituality. *Journal of General Internal Medicine*. 2003;18(1):38-43. doi:10.1046/j.1525-1497.2003.20403.x
15. McCord G. Discussing Spirituality With Patients: A Rational and Ethical Approach. *The Annals of Family Medicine*. 2004;2(4):356-361. doi:10.1370/afm.71
16. Stoller L, Blanchard E, Fowler M. Trigger Topics: Where Religion & Health Care Intersect. *MedEdPORTAL Publications*. Published online 2015. doi:10.15766/mep_2374-8265.10007
17. American Nurses Association, Health Ministries Association. *Faith Community Nursing : Scope and Standards of Practice.* American Nurses Association; 2005.
18. Heidari A, Kazemi A, Abbasi M, et al. Developing a charter of spiritual care for patients. *International Journal for Quality in Health Care*. 2020;33(1). doi:10.1093/intqhc/mzaa172
19. Varkey B. Principles of Clinical Ethics and Their Application to Practice. *Medical Principles and Practice*. 2021;30(1):17-28. doi:10.1159/000509119
20. Understanding Motivational Interviewing. Motivational Interviewing Network of Trainers (MINT). Accessed September 9, 2022. <https://motivationalinterviewing.org/understanding-motivational-interviewing>
21. Sinclair S, Beamer K, Hack TF, et al. Sympathy, empathy, and compassion: A grounded theory study of palliative care patients' understandings, experiences, and preferences. *Palliat Med*. 2017;31(5):437-447. doi:10.1177/0269216316663499
