# Supplementary material for: Enriching Spiritual Care in Medical Residents Through Cultural Humility and Courage
Source: MedEdPORTAL. 2024 Jul 26;20:11423. doi: 10.15766/mep_2374-8265.11423 (PMC11272909; doi:10.15766/mep_2374-8265.11423)
Supplement: Supplementary file 1 — Cultural Humility and Courage in Spiritual Care.pptxFacilitator Guide for Spiritual Care Session.docxSpiritual Care Reflection Questions.docxSpiritual Care Surveys.docx [file mep_2374-8265.11423-s001.zip › C. Spiritual Care Reflection Questions.docx]

**Spiritual Care Personal Reflection Questions**

1. Which attitude fits your current posture toward spiritual care? _____________
2. How do you define spirituality?
3. What is your percentage guess regarding religious prevalence in your region? _____________
4. How much potassium do you give the patient in the presented case? _____________
5. Why do you think doctors do not often ask patients about spirituality?
6. How would you personally respond to the patients in the ethics and motivational interviewing cases?
7. What gives your life meaning and purpose today? What might give your life meaning in suffering?
