# Supplementary material for: Enriching Spiritual Care in Medical Residents Through Cultural Humility and Courage
Source: MedEdPORTAL. 2024 Jul 26;20:11423. doi: 10.15766/mep_2374-8265.11423 (PMC11272909; doi:10.15766/mep_2374-8265.11423)
Supplement: Supplementary file 1 — Cultural Humility and Courage in Spiritual Care.pptxFacilitator Guide for Spiritual Care Session.docxSpiritual Care Reflection Questions.docxSpiritual Care Surveys.docx [file mep_2374-8265.11423-s001.zip › D. Spiritual Care Surveys.docx]

**Spiritual Care Imbedded Pre/Post-Survey**

**Which attitude fits your current posture toward spiritual care?**

1. Embracing
2. Pragmatic
3. Guarded
4. Rejecting

**Spiritual Care Emailed Post-Course Survey**

**Respond to the following statements on a five-point scale**

|  | **Strongly disagree** | **Disagree** | **Neutral** | **Agree** | **Strongly agree** |
| --- | --- | --- | --- | --- | --- |
| **1. The presentation was well organized.** | 1 | 2 | 3 | 4 | 5 |
| **2. The presenter captured my attention.** | 1 | 2 | 3 | 4 | 5 |
| **3. There was ample time for questions.** | 1 | 2 | 3 | 4 | 5 |
| **4. The content of the presentation was relevant to my professional development.** | 1 | 2 | 3 | 4 | 5 |
| **5. The speaker met their objectives.** | 1 | 2 | 3 | 4 | 5 |
| **6. Overall, I was satisfied with the presentation.** | 1 | 2 | 3 | 4 | 5 |

**Please offer any additional comments:**
